# Ligand‐Controlled Diastereoselective Cobalt‐Catalysed Hydroalkynylation of Terminal Alkynes to *E*‐ or *Z*‐1,3‐Enynes

**DOI:** 10.1002/chem.202001697

**Published:** 2020-09-07

**Authors:** Sebastian M. Weber, Jona Queder, Gerhard Hilt

**Affiliations:** ^1^ Institut für Chemie Carl von Ossietzky Universität Oldenburg Carl-von-Ossietzky Straße 9-11 26129 Oldenburg Germany; ^2^ Fachbereich Chemie Philipps-Universität Marburg Hans-Meerwein-Straße 4 35043 Marburg Germany

**Keywords:** alkynes, cobalt, enynes, hydroalkynylation, stereoselectivity

## Abstract

A diastereoselective hydroalkynylation of terminal alkynes to form the head‐to‐head dimerization products by two different cobalt‐phosphine catalyst system is reported. The use of the bidentate ligand dppp and additional triphenylphosphine led to the selective formation of the *(E)*‐1,3‐enynes (*E:Z*>99:1) in good to excellent yields, while the tridentate ligand TriPhos led to the corresponding (*Z*)‐1,3‐enynes in moderate to good yields with excellent stereoselectivities (up to *E:Z=*1:99). Both pre‐catalysts are easy to handle, because of their stability under atmospheric conditions. The optimized reaction conditions were identified by the Design of Experiments (DoE) approach, which has not been used before in cobalt‐catalysed reaction optimisation. DoE decreased the number of required reactions to a minimum.

## Graphical abstract

**Cobalt catalysis at a crossroad**: The choice of the ligands in a cobalt‐catalysed hydroalkynylation of terminal alkynes is decisive to form the dimerization products in a diastereoselective fashion. The *(E)*‐1,3‐enynes are formed in good to excellent yields and outstanding selectivities (*E:Z*>99:1), while the corresponding (*Z*)‐1,3‐enynes were isolated in moderate to good yields with excellent stereoselectivities (up to *E:Z=*1:99).

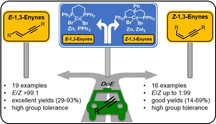

Alkynes are versatile building blocks to construct various types of cyclic and acyclic molecules. The dimerization of alkynes can lead in rare occasions to transition‐metal‐stabilized *anti*‐aromatic cyclobutadiene complexes or to acyclic dimers.^1^ The transition‐metal catalysed addition of an terminal alkyne to itself, the so called hydroalkynylation (dimerization), can be realized to generate three possible products (Scheme 1).

**Scheme 1:**
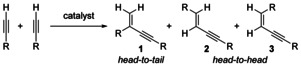
Possible hydroalkynylation products of terminal alkynes.

The transition‐metal‐catalysed Markovnikov‐type addition (head‐to‐tail) leads to the conjugated enyne **1** whereas the *anti*‐Markovnikov addition generates either the *E*‐ or the *Z*‐configured enynes **2** and **3** (head‐to‐head products). For the selective formation of only one of these products the catalyst must control the regio‐ and the stereochemistry of this transformation.^2^ Also, the catalyst must allow the coordination of two alkynes to the transition metal but not the incorporation of a third alkyne which would lead to the well‐known cyclotrimerization^3^, ^4^ towards arenes or an undesired polymerization towards polyacetylenes. In the past, excellent results for the dimerization of terminal alkynes were obtained when precious metal catalysts, for example, Pd, were applied to realize the hydroalkynylation products of type **1** in good to excellent yields.^5^ Recently, an iron(II) catalysed approach towards products of type **1** was investigated by Song.^6^ Cobalt–hydride^7^ and cobalt–phosphine catalysts in combination with visible light^8^ were utilized to generate the products of type **2** whereas the formation of the *Z*‐configured enynes of type **3** has been reported for example, iron‐9 and lanthanide‐based^10^ catalyst systems. An (*E*)‐selective cross‐hydroalkynylation was reported very recently where a cobalt(0/+II) couple was proposed to be the catalytic species in a cross‐dimerization of a terminal alkyne with a bulky group, such as Me_3_Si and another sterically less hindered terminal alkyne (Scheme 2).^11^

**Scheme 2:**
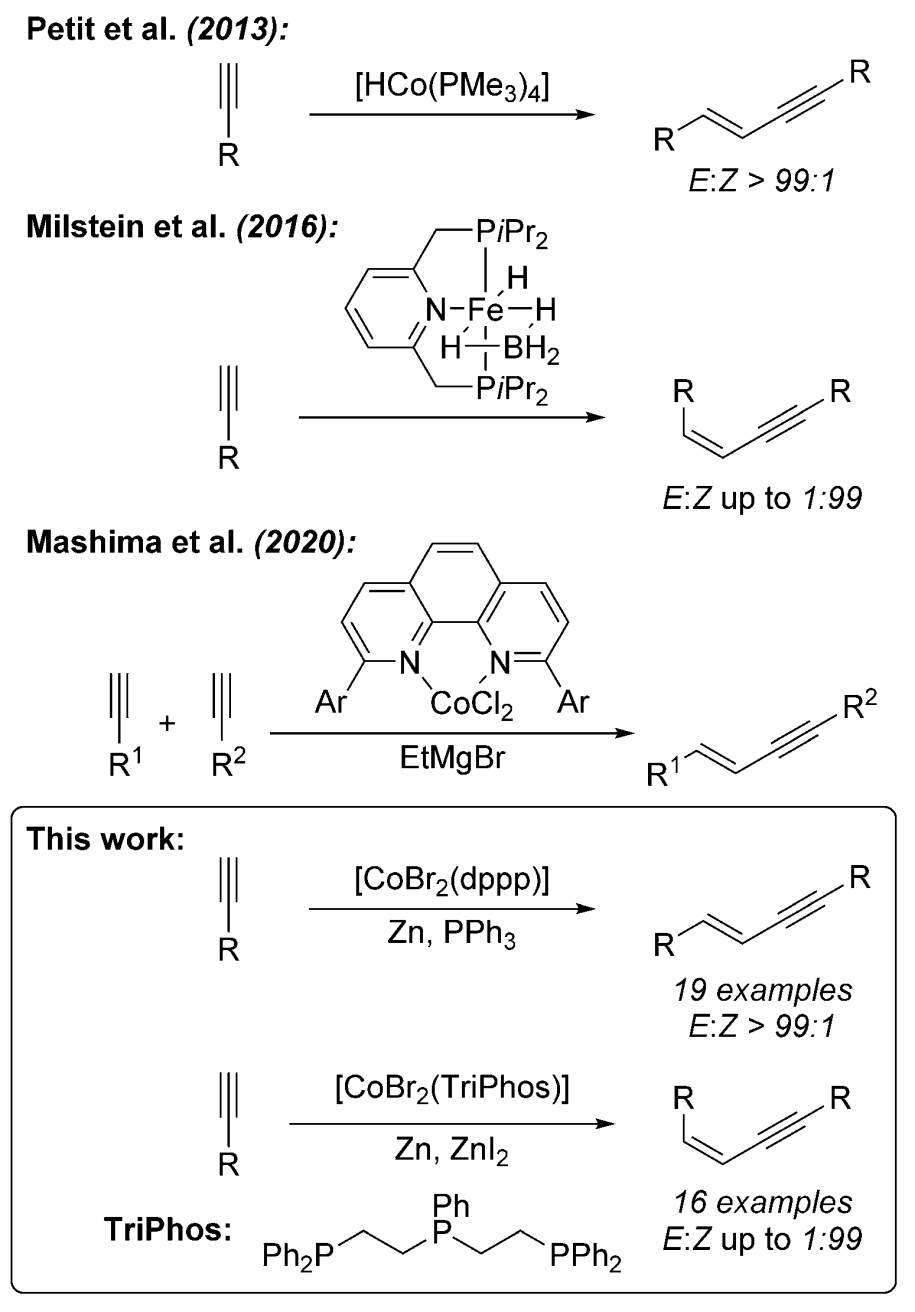
Recent progress on transition metal catalysed (cross)‐hydroalkynylation of terminal alkynes.^7^, ^9^, ^11^

Considering our ongoing interest in cobalt‐catalysed reactions, we experienced in several investigations that the choice of the ligand, and the resulting new coordination sphere, is very decisive for the preference for a single reaction pathway. Rather small changes can have a profound impact on the chemo, regio, or stereoselective outcome of cobalt‐catalysed reactions.^12^, ^13^, ^14^ Accordingly, the herein presented investigation was initiated by the observation of a dimerization product when an additional ligand was added to a simple cobalt catalyst for the desired cyclotrimerization reaction of a terminal alkyne utilizing dppp (1,3‐bis(diphenylphosphino)‐propane as the bidentate ligand. At first, triethylamine was used as an additive, which could also act as a base for a possible diprotonation of the alkyne coordinated to the cobalt centre. Thereafter, we tested a variety of other nitrogen and phosphorous donor ligands as additives to alter the reaction pathway from the original [2+2+2] cycloaddition reaction towards a hydroalkynylation of alkynes. Besides triethylamine, other simple amines resulted already in good results, but the addition of triphenylphosphine as the co‐ligand gave the best results, concerning yield and selectivity towards the *E*‐1,3‐enyne **2**. In the past, we experienced that the use of pyridine‐imine‐type ligands resulted in a highly selective cyclotrimerization of terminal and internal alkynes.^15^ However, the use of additional ligands in cobalt‐pyridine‐imine‐type or pyridine‐diimine pincer‐type catalyst systems did not result in an altered reaction pathway towards the desired dimerization (hydroalkynylation) of alkynes. After identification of the best bidentate ligand (dppp) and additive (PPh_3_) for the products of type **2** we optimized the reaction conditions by the Design of Experiments (DoE) approach^15^ using 4‐fluoro‐1‐ethynylbenzene as test substrate, regarding the following parameters: catalyst loading (2–10 mol %, continuous), PPh_3_ equivalents (1.0–3.0 equiv related to the catalyst loading, continuous), temperature (0 °C−55 °C, continuous), time (0.5 h–6 h, continuous), substrate concentration (0.2 m–1.5 m, continuous) and zinc iodide (yes, no, categorical) were optimized in only 25 experiments. Also, quadratic terms and possible cross interaction between the parameters were considered. All other categorical parameters, such as the solvent (THF, **MeCN**, CH_2_Cl_2_, DMF) or cobalt source (**CoBr_2_**, CoCl_2_, Co(BF_4_)_2_, Co(ClO_4_)_2_), were tested before starting with this D‐optimal design (for further information, see Supporting Information, Table S1–S4).

After running the experiments (for the detailed reaction conditions, see Supporting Information Table S5) three experiments were duplicated for the determination of the lack of fit of the model (Figure 1).

**Figure 1:**
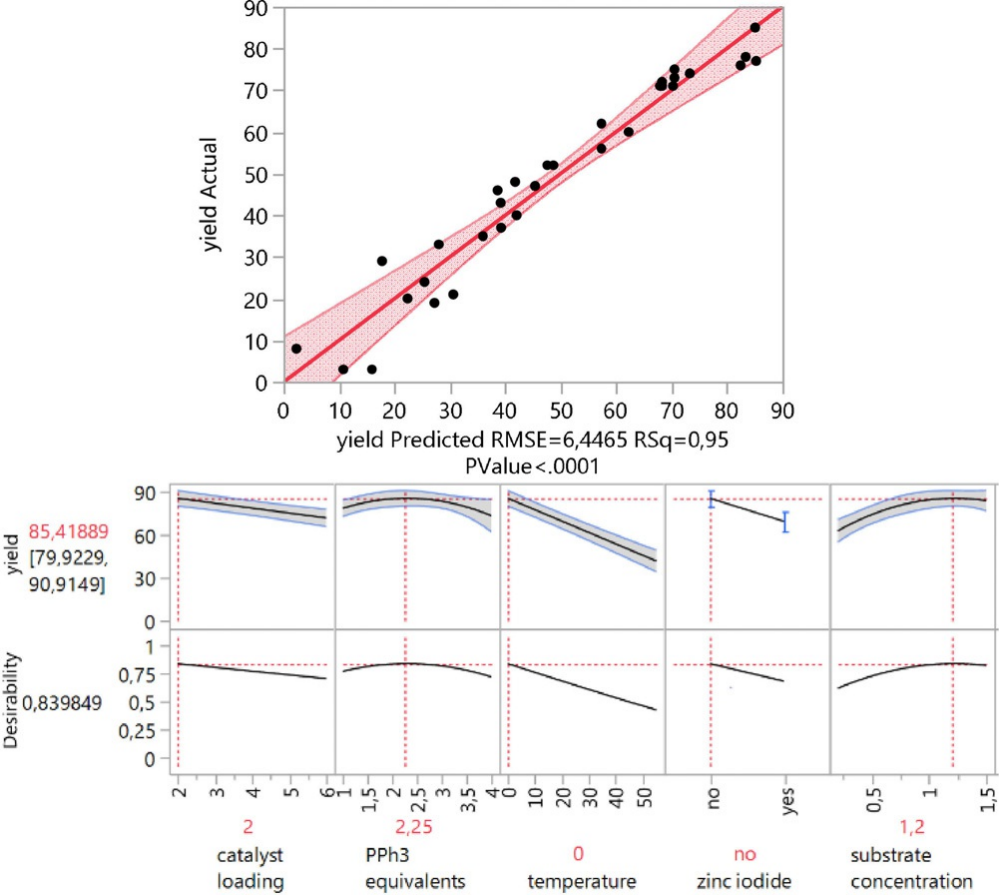
Reaction optimization for the *E*‐selective hydroalkynylation of terminal alkynes. The predicted yields are plotted against the measured yields. Total run of experiments: 31; 25 for the model, three duplicates for the lack of fit, and three additional reactions for the PPh_3_ equivalents. The predicted optimized reaction conditions can be seen in the boxes below. The yields were determined by GC/FID analysis and ^19^F NMR spectroscopy using mesitylene and hexafluorobenzene as internal standard.

The resulting model consisted of eight factors with a R^2^=0.95. The most important factors were the temperature, the use of zinc iodide, the cross‐interaction temperature ^.^ substrate concentration and the catalyst loading (*p*‐values<0.01). Besides, the quadratic terms of the substrate concentration (*p*‐value=0.01) and the triphenylphosphine equivalents (*p*‐value=0.02) had also an impact to the design.

Interestingly, the model shows that a low catalyst loading (2.0 mol %) is optimal for the reaction with 4.5 mol % of triphenylphosphine added to the catalyst. In contrast to our previous reported reactions, containing cobalt catalysts and alkynes, the use of zinc iodide has a counterproductive influence concerning the yield, possibly indicating that one bromine atom could stay at the central atom.

The predicted reaction conditions show some similarities to the described reaction conditions by Petit et al., who used a preformed hydrido‐cobalt(I) complex as active species (compare Scheme 2).^7^ We therefore conclude that cobalt(I) is the active species which is easily generated in situ by reduction of the air stable cobalt(II) pre‐catalyst.

With the optimized conditions in hand, a variety of terminal alkynes were transformed to synthesize the desired products of type **2**. Most strikingly, all the products were obtained in outstanding *E*‐selectivity (Scheme 3) and the products of type **1** or **3** were not observed at all.

**Scheme 3:**
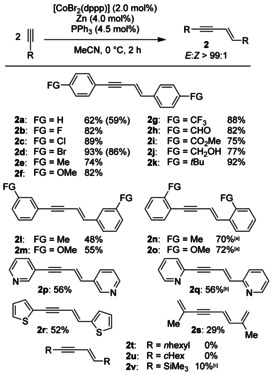
Substrate scope of *E*‐selective hydroalkynylation of terminal alkynes. All reactions were conducted on a 1.00 mmol scale. Yields are given as isolated yields after purification via column chromatography. The substrates **2 a** and **2 c** were also synthesized in a gram‐scale. The yields of the gram‐scale synthesis are given in parenthesis. [a] Reaction time: 18 h. [b] Reaction time: 48 h. [c] The product was obtained as an inseparable mixture of isomers.

The cobalt catalyst system tolerates a broad range of functional groups, for example, aryl halides (**2 b**–**2 d**) in good to excellent yields and excellent regioselectivities. Besides simple alkyl groups (**2 e**) methoxy (**2 f**), unprotected benzylic alcohols (**2 j**), aldehyde (**2 h**), and ester substituents (**2 i**) are well tolerated. The alkynyl arenes with substituents in *meta*‐position gave yields around 48 %. Normally, *ortho*‐substituted alkynyl arenes with donor‐substituents in *ortho*‐position cause more problems, due to their steric demand near to the catalyst.^4^, ^17^ Interestingly, the products **2 n** and **2 o** gave considerably better yields between 70–72 % compared to those with the same substituents in *meta*‐position (**2 l**/**2 m**). Also, pyridinyl substituted alkynes were tolerated and gave the desired products **2 p**/**q** in good yields, as well as the ethynyl thiophene derivative which gave the products **2 r** in a moderate yield. In addition, the rather volatile trieneyne **2 s** could be isolated in 29 % yield from the conjugated enyne and might be of interest for follow‐up reactions. It should be mentioned that the yield also depends on the solubility of the reaction product in acetonitrile. The highest yields were obtained, where the product precipitates out of the reaction mixture (e.g. **2 c** and **2 d**). Unfortunately, alkyl‐substituted alkynes, such as 1‐octyne or cyclohexylacetylene gave no desired product. In case of 1‐octyne only small amounts of the alkyne reacted after a long time, leading only to the cyclotrimerization product. The cyclohexyl moiety led to the formation of oligomers which were not characterized. The use of trimethylsilyl acetylene gave similar results and pure samples of the desired product (**2 v**) could not be obtained.

In the next step of the investigation, the bidentate phosphine ligand was exchanged to the more rigid tridentate linear TriPhos ligand (see Scheme 4) which led surprisingly to the selective formation of the *Z*‐configured head‐to‐head dimerization product of type **3**, which has not been described in the literature before using cobalt(0) or cobalt(I) catalyst systems. All other tested bi‐ or tridentate phosphine ligands with different bite angles neither changed the selectivity towards the *Z*‐configured product, nor to the head‐to‐tail dimerization product of type **1**.

**Scheme 4:**
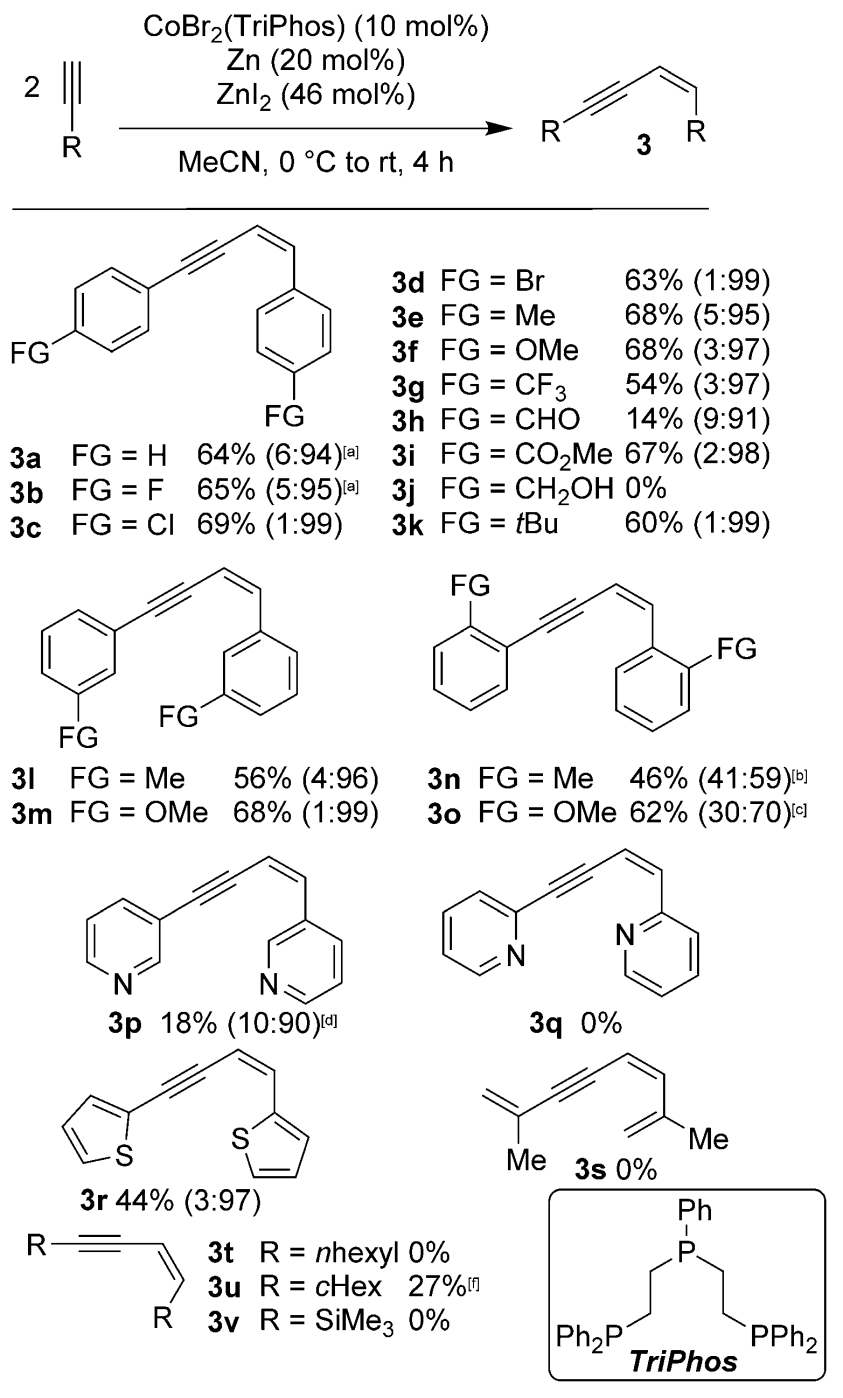
Substrate scope of *Z*‐selective hydroalkynylation of terminal alkynes. All reactions were conducted on a 1.00 mmol scale. Yields are given as isolated yields after purification via column chromatography. *E*/*Z* ratios are given in parenthesis. [a] Temperature: 37 °C, reaction time: 3 h. [b] Reaction time: 24 h. [c] Reaction time: 18 h. [d] Reaction time: 28 h. [e] Zinc iodide (10 mol %) was used, temperature: 37 °C, reaction time: 24 h. [f] The product was obtained as an inseparable mixture consisting of isomers and cyclotrimerization product.

Next, we optimized the reaction conditions for the *Z*‐selective dimerization using the Design of Experiments approach. Before running a d‐optimal design (determinant‐optimal design), acetonitrile as solvent and cobalt dibromide as cobalt source were set as standard conditions. Unfortunately, no amine‐ or phosphine additive as ligand had a positive effect to the reaction (see SI, Tables S6–S8). All additives led to a lower *E*/*Z* selectivity, so that no additive was added to the model. Accordingly, the model consisted of the following continuous parameters: catalyst loading (2–10 mol %), zinc iodide (4–100 %), temperature (−10–60 °C), substrate concentration (0.2 m–1.5 m) and time (3–24 h). The results of the D‐optimal design are illustrated in Figure 2.

**Figure 2:**
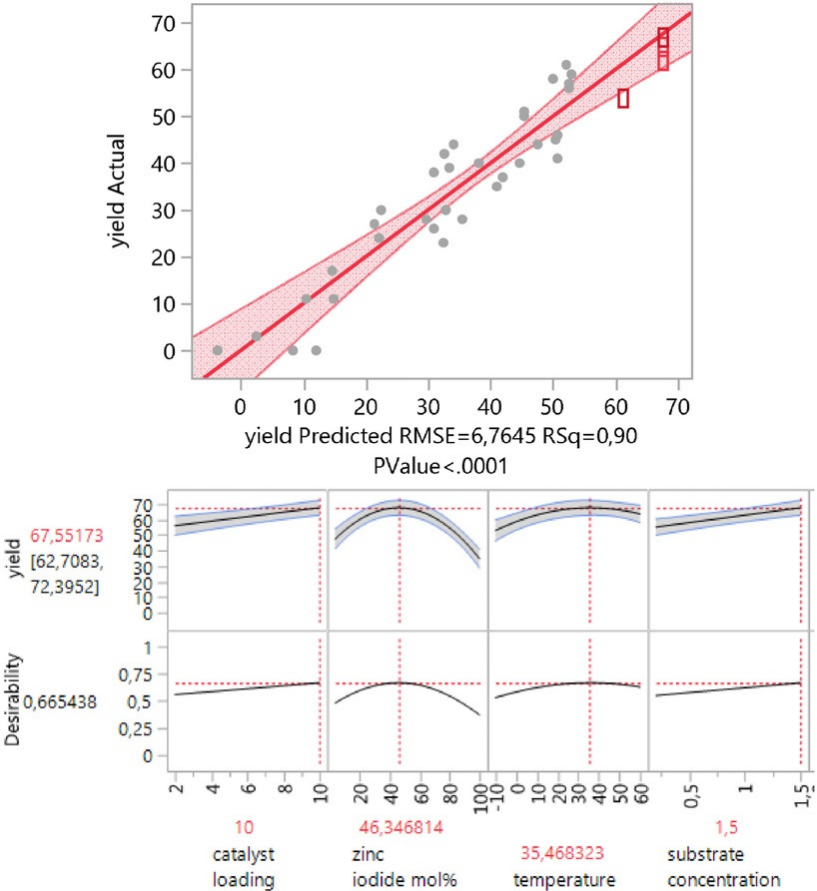
Reaction optimization for the *Z*‐selective hydroalkynylation of terminal alkynes. The predicted yields are plotted against the measured yields. Total run of experiments: 39; 30 for the design, six replicates for the lack of fit, and three additional reactions with the optimized reaction conditions with different length in time. The predicted optimized reaction conditions can be seen in the boxes below. The yields were determined by GC/FID and ^19^F NMR spectroscopy using mesitylene and hexafluorobenzene as internal standard.

The original design consisted of only 16 reactions, where the equivalent of zinc iodide was set between 4–60 mol %. Because of the high significance of the quadratic term of the zinc iodide equivalents, the range was expanded to 100 mol % and the design was extended to consider all quadratic terms of each parameter and possible cross interactions between them. The design consisted of six factors with a R^2^=0.90. All *p*‐values of the six factors were lower than <0.01, showing that each factor is important. The most important factor was the quadratic term of zinc iodide equivalent, followed by the substrate concentration and catalyst loading. The quadratic term of the temperature indicated a slightly elevated temperature of 37 °C.

Under those optimized reaction conditions, the screening substrate (4‐fluoro‐1‐ethynylbenzene) showed full conversion after three hours reaction time and the product **3 b** was isolated in 65 % yield (predicted yield 67 %).

With the optimized reaction conditions in hand, we started our investigation concerning the substrate scope. While products **3 a** and **3 b** were isolated in good yields with high *Z*‐selectivities (up to 95:5), the products **3 c**, **3 d** and **3 f** were formed in lower *E*/*Z* ratios and lower yields at 37 °C. Because of this observation, the temperature was decreased to room temperature, resulting in excellent selectivities and slightly increased yields. Compared with the *E*‐selective hydroalkynylation, the substrates **3 a**–**3 f** gave good yields between 64–69 % and high preference for the Z‐configured products. While the ester substituted substrate **3 i** gave a good yield of 67 %, aldehyde, and alcohol moieties were not tolerated. This could be explained by the need of the Lewis acid ZnI_2_, leading to a full conversion towards polymers of unknown constitution. A decrease of the amount of zinc iodide to 10 mol % and an increased temperature of 37 °C resulted in the formation of **3 h** in 14 % yield and polymers as side products. Similar observations were made for products **3 p** and **3 q**. Only 3‐ethinylpyridine gave a low isolated yield (18 %) of **3 p**.The *ortho*‐substituted products **3 n** and **3 o** needed longer reaction times and showed lower *E*/*Z*‐selectivities, possibly based on steric interactions between the ligand‐sphere of the active species and the substrates. The products **3 t** and **3 v** could not be observed, while **3 u** could be isolated as an *E*/*Z* mixture, accompanied with the cyclotrimerization product. The catalyst system seems to tolerate alkyl moieties and the optimization towards one reaction pathway is under current investigation in our laboratory.

The change of the selectivity and the mechanism is not easy explained. At first, we believe that the active species in both reaction pathways needs a reduction step from cobalt(II) to cobalt(I) by the reducing agent zinc. Stronger reducing agents like magnesium failed in the *Z*‐selective hydroalkynylation, leading to no product formation, while ethyl magnesium bromide (2.0 equiv referred to the catalyst loading) leads to a lower conversion and lower yield of **3** (see SI, Table S9). However, the higher amount of zinc iodide cannot be easily explained. While the *E*‐selective catalyst system could react via a hydrocobaltation pathway as described by Petit et al.,^6^ it is likely that tridentate ligands may enable the formation of a cobalt‐vinylidene species, which leads to the selective formation of the *Z*‐configured product **3**.^2^, ^9^, ^18^

Mechanistic insights to the *Z*‐selective dimerization process are under investigation in our laboratory, concerning oxidation state and intermediates of the active species.

In conclusion, we realized a diastereoselective synthesis of *E*‐ and *Z*‐enynes. To reduce the number of experiments of the optimization, we performed the reaction optimization with the Design of Experiments approach and identified the crucial parameters for both reactions. We were able to generate the active cobalt species in an easy manner without crucial decrease of the yield. Next, we have developed a novel synthesis for *Z*‐enynes, which includes cobalt(I) as the active species for the first time. Both catalyst systems are kept simple considering the possibility to reproduce the reported methods without the need of strictly air‐ and moisture free conditions.

## Conflict of interest

The authors declare no conflict of interest.

## Supporting information

As a service to our authors and readers, this journal provides supporting information supplied by the authors. Such materials are peer reviewed and may be re‐organized for online delivery, but are not copy‐edited or typeset. Technical support issues arising from supporting information (other than missing files) should be addressed to the authors.

Supplementary
