# Supplementary material for: Ligand‐Controlled Diastereoselective Cobalt‐Catalysed Hydroalkynylation of Terminal Alkynes to E‐ or Z‐1,3‐Enynes
Source: Chemistry. 2020 Sep 7;26(53):12129–33. doi: 10.1002/chem.202001697 (PMC7540399; doi:10.1002/chem.202001697)
Supplement: Supplementary file 1 — Supplementary [file CHEM-26-12129-s001.pdf]

Chemistry–A European Journal

Supporting Information

Ligand-Controlled Diastereoselective Cobalt-Catalysed Hydroalkynylation of Terminal Alkynes to *E*- or *Z*-1,3-Enynes

Sebastian M. Weber,[a, b] Jona Queder,[a] and Gerhard Hilt*[a]

Table of Contents

Experimental Procedures S2

General Information S2

Synthesis of (*E*)-But-1-en-3-ynes S3

Synthesis of (*Z*)-But-1-en-3-ynes S11

Reaction Optimization for the Synthesis of *E*-1,3-Enynes S18

Determination of categorical parameters S18

Reaction Optimization for the *E*-selective hydroalkynylation of terminal alkynes using *Design of Experiments* S19

Reaction Optimization for the Synthesis of *Z*-1,3-Enynes S23

Determination of categorical parameters S23

Reaction Optimization for the *Z*-selective hydroalkynylation of terminal alkynes using *Design of Experiments* S24

Literature S29

NMR spectra S30

Experimental Procedures

General Information

All reactions with water- and/or air-sensitive starting materials were carried out in pre-dried glass wares under Argon atmosphere utilizing standard Schlenk techniques. All used solvents were dried over molecular sieves (3 Å) and they were degassed prior to use. Thin layer chromatography (TLC) was carried out on prefabricated plates (silica gel 60, F254 with fluorescence indicator) by *Macherey Nagel*. Column Chromatography was carried out on silica gel 60 (40-63 µm, 230-400 mesh) by *Macherey Nagel*. Commercially available chemicals were used without further purification.

The alkyne substrates 1-ethynyl-4-flurobenzene[1], 1-ethynyl-4-bromobenzene[2], 1-ethynyl-4-methylbenzene[2], 1-ethynyl-4-methoxybenzene[2], 1-ethynyl-4-(trifluromethyl)benzene[3], methyl 4-ethynylbenzoate[4], (4-ethynylpehnyl)methanol[5], 1-*tert*-butyl-4-ethynyl-benzene[5], 1-ethynyl-3-methylbenzene[5], 1-ethynyl-3-methoxybenzene[6], 1-ethynyl-2-methylbenzene[5], 1-ethynyl-2-methoxybenzene[6], 3-ethynylpyridine[4] and 2-ethynylthiophene[3] were synthesized via Sonogashira cross-coupling[7], followed by protodesilylation with potassium carbonate in methanol. The analytical data of each substrate are in accordance with the literature.

Cobalt dibromide was dried at 150 °C *in vacuo* and stored under Argon atmosphere. Zinc iodide was dried at 230 °C *in vacuo* and stored und Argon atmosphere.

^{1}H and ^{13}C NMR were either recorded on a *Bruker Fourier 300HD* or a *Bruker Avance III 500HD* spectrometer at room temperature utilizing preset pulse programs. The residual solvent signal ($CDCl_3$: ^{1}H NMR: 7.26 ppm, ^{13}C NMR: 77.16 ppm, DMSO-d_6: ^{1}H NMR: 2.50 ppm, ^{13}C NMR: 39.52 ppm) was used for calibration referred to tetramethylsilane.[8] The chemical shifts are given in parts per million (ppm). The multiplicity of each signal is reported as singulett (s), doublet (d), triplet (t), quartet (q), multiplet (m) or combinations thereof. Multiplicities are reported as they were measured, and they might disagree with the expected multiplicity of a signal. ^{19}F NMR were recorded on a *Bruker Avance III 500HD* spectrometer at room temperature utilizing preset pulse programs. The chemical shifts are given in ppm. Hexafluorobenzene (^{19}F NMR: −164.9 ppm[9]) was used as an internal standard. Infrared spectra were recorded on a *Shimadzu IR Spirit QATR-S* spectrometer. The absorption bands are given in wave numbers (cm^{-1}). High resolution mass spectra (EI) were recorded on a *Thermo Scientific DFS* spectrometer. The ionization was accomplished by electron ionization (EI) at an energy of 70 eV. Electron spray ionization (ESI) spectra were recorded on a *Waters Q-Top Premier* spectrometer.

GC/MS spectra were recorded on a *Shimadzu GC QP2020* spectrometer, equipped with an Optima 5HT column (length: 30 m, inner diameter: 0.25 mm, film thickness: 0.25 µm), fabricated by *Macherey Nagel*. Ionization was accomplished by electron ionization (EI) at 70 eV. GC/FID spectra were recorded on a *Shimadzu GC 2010 Plus* gas chromatograph, equipped with an Optima 5 MS column (length: 15 m, inner diameter: 0.25 mm, film thickness: 0.25 µm), fabricated by *Macherey Nagel*.

Melting points were measured on a *Gallenkamp MPD 350 BM 2.5* device. All specified melting points are not corrected.

(*E*)/(*Z*) ratios of the pure products were determined by the baseline separated signals in the corresponding ^{1}H NMR spectra.

Design of Experiments was performed using *JMP13* software package by *SAS* (version 13.2.1, *SAS Institute Inc.*, Cary, NC, USA, © 2016).

Synthesis of the cobalt pre-catalysts

Synthesis of [CoBr$_2$(dppp)]

Following a literature-known procedure[10] anhydrous cobalt dibromide (1.04 g, 4.75 mmol, 1.00 equiv.) was added to a pre-dried reaction vessel and dried *in vacuo* for 15 min. The salt was dissolved in dry THF (40.0 mL). To the blue solution 1,3-bis(diphenylphosphino)propane (1.96 g, 4.75 mmol, 1.00 equiv.) was added and the resulting dark green suspension was stirred for 16 h. The mixture was concentrated under reduced pressure and the catalyst was precipitated by the addition of *n*-pentane (20 mL). The residue was filtered and washed with *n*-pentane and diethyl ether. The pre-catalyst was dried *in vacuo* and isolated as a blue-green amorphous solid (2.99 g, 4.75 mmol, quant.). The catalyst was used without further purification.

Synthesis of [CoBr$_2$(TriPhos)]

Under Argon atmosphere anhydrous cobalt dibromide (656 mg, 3.00 mmol, 1.00 equiv.) was added to a pre-dried reaction vessel and dried *in vacuo* for another 15 min. The salt was dissolved in dry THF (60.0 mL). To the blue solution bis(diphenylphosphinoethyl)phenylphosphine (1.60 g, 3.00 mmol, 1.00 equiv.) dissolved in THF (30.0 mL) was added slowly and the resulting dark red suspension was stirred for 16 h. Within the addition the suspension solidified for a short period of time. The solvent was removed under reduced pressure and the residue washed with *n*-pentane and diethyl ether. The pre-catalyst was dried *in vacuo* and isolated as a red-brown amorphous solid (2.25 g, 3.00 mmol, quant.). The catalyst was used without further purification.

Synthesis of (*E*)-But-1-en-3-ynes

General Procedure 1:

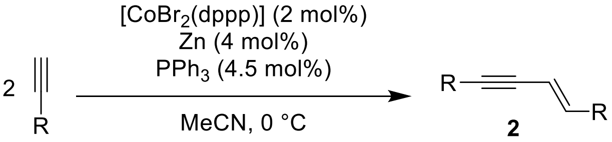

Under Argon atmosphere [CoBr$_2$(dppp)] (2 mol%), zinc dust (4 mol%) and triphenylphosphine (4.5 mol%) were added to a pre-dried reaction vessel. The solids were dried 10 min *in vacuo*. The catalyst system was dissolved in dry acetonitrile (0.8 mL/mmol). The resulting dark suspension was shortly heated to approximately 80 °C, whereas a color change from dark blue/green to green appeared. The resulting solution was cooled to 0 °C and the corresponding alkyne (1.00 equiv.) was added. The reaction mixture was stirred until complete conversion was determined via GC/MS and TLC. The mixture was diluted with CH_2Cl_2 and filtered over a short plug of silica gel (eluent: *n*-pentane:CH_2Cl_2 = 3:1 or CH_2Cl_2, depending on the polarity of the substrate). The solvent was removed under reduced pressure. The crude product was purified via column chromatography (*n*-pentane:CH_2Cl_2 or *n*-pentane:ethyl acetate).

(E)-But-1-en-3-yne-1,4-diylbenzene (2a)

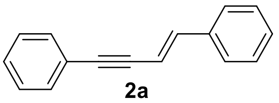

2a

According to General Procedure 1 the title compound was prepared using phenylacetylene (102 mg, 1.00 mmol, 1.00 equiv.), [CoBr$_2$(dppp)] (12.6 mg, 20.0 µmol, 0.02 equiv.), zinc dust (2.6 mg, 40.0 µmol, 0.04 equiv.) and triphenylphosphine (11.8 mg, 45.0 µmol, 0.05 equiv.) in acetonitrile (0.8 mL). The reaction mixture was stirred 2 h at 0 °C. The product was obtained after column chromatography (eluent: *n*-pentane:CH_2Cl_2 = 10:1) as a colorless solid (61.7 mg, 308 µmol, 62%).

melting point: 98 – 101 °C.

^{1}H NMR (300 MHz, $CDCl_3$) δ = 7.47 (ddd, *J* = 15.7, 7.7, 2.6 Hz, 4H), 7.39 – 7.28 (m, 6H), 7.06 (d, *J* = 16.2 Hz, 1H), 6.41 (d, *J* = 16.2 Hz, 1H) ppm.

^{13}C NMR (75 MHz, $CDCl_3$) δ = 141.4, 136.5, 131.7 (2C), 128.9 (2C), 128.8, 128.5 (2C), 128.3, 126.4 (2C), 123.5, 108.3, 91.9, 89.0 ppm.

IR (ATR): $\tilde{\nu}$ = 3032, 1956, 16114, 1596, 1614, 1593, 1486, 1446, 1440, 1336, 1282, 1070, 1024, 947, 916, 747, 687, 534, 514 cm^{-1}.

HRMS $(EI)^+$: *m/z* for $C_{16}H_{12}$ $[M]^+$: calc.: 204.0934, found: 204.0928

The title compound was also synthesized on a 10 mmol scale following General Procedure 1. The product was obtained as a colorless solid (603 mg, 2.95 mmol, 59%).

(E)-4,4'-(But-1-en-3-yne-1,4-diyl)bis(fluorobenzene) (2b)

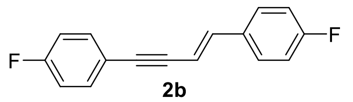
2b

According to General Procedure 1 the title compound was prepared using 1-ethynyl-4-fluorobenzene (120 mg, 1.00 mmol, 1.00 equiv.), [$CoBr_2$(dppp)] (12.6 mg, 20.0 µmol, 0.02 equiv.), zinc dust (2.6 mg, 40.0 µmol, 0.04 equiv.) and triphenylphosphine (11.8 mg, 45.0 µmol, 0.05 equiv.) in acetonitrile (0.8 mL). The reaction mixture was stirred 2 h at 0 °C. The product was obtained after column chromatography (eluent: *n*-pentane:CH_2Cl_2 = 10:1) as a colorless solid (98.0 mg, 408 µmol, 82%).

melting point: 118 – 121 °C.

^{1}H NMR (500 MHz, $CDCl_3$) δ = 7.45 (dd, *J* = 8.7, 5.5 Hz, 2H), 7.39 (dd, *J* = 8.7, 5.4 Hz, 2H), 7.06 – 7.01 (m, 4H), 6.99 (d, *J* = 16.8 Hz, 1H), 6.27 (d, *J* = 16.2 Hz, 1H) ppm.

^{13}C NMR (126 MHz, $CDCl_3$) δ = 163.1 (d, *J* = 249.0 Hz), 162.6 (d, *J* = 249.7 Hz), 140.2, 133.5 (d, *J* = 8.3 Hz, 2C), 132.7 (d, *J* = 3.4 Hz), 128.1 (d, *J* = 8.2 Hz, 2C), 119.6 (d, *J* = 3.6 Hz), 116.0 (d, *J* = 16.5 Hz, 2C), 115.8 (d, *J* = 17.0 Hz, 2C), 107.9, 90.7, 88.5 ppm.

^{19}F NMR (470 MHz, $CDCl_3$) δ = −114.0, −115.5 ppm.

IR (ATR): $\tilde{\nu}$ =3100, 3062, 3037, 1900, 1596, 1584, 1502, 1414, 1306, 1294, 1226, 1159, 1094, 1011, 960, 943, 856, 834, 814, 786, 670, 533 cm^{-1}.

HRMS $(EI)^+$: *m/z* for $C_{16}H_{10}F_2$ $[M]^+$: calc.: 240.0745, found: 240.0744.

(E)-4,4'-(But-1-en-3-yne-1,4-diyl)bis(chlorobenzene) (2c)

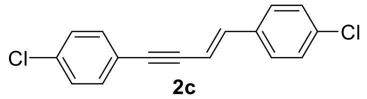
2c

According to General Procedure 1 the title compound was prepared using 1-chloro-4-ethynylbenzene (137 mg, 1.00 mmol, 1.00 equiv.), [$CoBr_2$(dppp)] (12.6 mg, 20.0 µmol, 0.02 equiv.), zinc dust (2.6 mg, 40.0 µmol, 0.04 equiv.) and triphenylphosphine (11.8 mg, 45.0 µmol, 0.05 equiv.) in acetonitrile (0.8 mL). The reaction mixture was stirred 2 h at 0 °C. The product was obtained after column chromatography (eluent: *n*-pentane:CH_2Cl_2 = 10:1) as a light-yellow solid (121 mg, 444 µmol, 89%).

melting point: 175 – 177 °C.

^{1}H NMR (300 MHz, $CDCl_3$) δ = 7.43 – 7.25 (m, 8H), 7.00 (d, *J* = 16.2 Hz, 1H), 6.34 (d, *J* = 16.2 Hz, 1H) ppm.

^{13}C NMR (75 MHz, $CDCl_3$) δ = 140.4, 134.8, 134.6, 134.5, 132.9 (2C), 129.1 (2C), 128.9 (2C), 127.6 (2C), 121.9, 108.6, 91.2, 89.7 ppm.

IR (ATR): $\tilde{\nu}$ = 3084, 3052, 3029, 1902, 1780, 1586, 1486, 1404, 1396, 1297, 1279, 1174, 1090, 1011, 954, 857, 824, 804, 763, 707, 690, 637, 631, 581, 523 cm^{-1}.

HRMS $(EI)^+$: *m/z* for $C_{16}H_{10}Cl_2$ $[M]^+$: calc.: 272.0154, found: 272.0146.

(E)-4,4'-(But-1-en-3-yne-1,4-diyl)bis(bromobenzene) (2d)

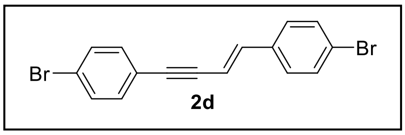

According to General Procedure 1 the title compound was prepared using 1-bromo-4-ethynylbenzene (181 mg, 1.00 mmol, 1.00 equiv.), $[CoBr_2(dppp)]$ (12.6 mg, 20.0 µmol, 0.02 equiv.), zinc dust (2.6 mg, 40.0 µmol, 0.04 equiv.) and triphenylphosphine (11.8 mg, 45.0 µmol, 0.05 equiv.) in acetonitrile (0.8 mL). The reaction mixture was stirred 2 h at 0 °C. The product was obtained after column chromatography (eluent: *n*-pentane:CH_2Cl_2 = 10:1) as a light-yellow solid (168 mg, 464 µmol, 93%).

melting point: 196 – 199 °C.

^{1}H NMR (300 MHz, $CDCl_3$) δ = 7.48 (dd, *J* = 8.5, 1.8 Hz, 4H), 7.40 – 7.23 (m, 4H), 6.98 (d, *J* = 16.2 Hz, 1H), 6.35 (d, *J* = 16.2 Hz, 1H) ppm.

^{13}C NMR (75 MHz, $CDCl_3$) δ = 140.5, 135.2, 133.1 (2C), 132.1 (2C), 131.8 (2C), 127.9 (2C), 122.9, 122.7, 122.3, 108.7, 91.4, 89.8 ppm.

IR (ATR): $\tilde{\nu}$ = 3027, 1909, 1579, 1394, 1320, 1279, 1253, 1176, 1099, 1070, 1009, 961, 859, 823, 806, 753, 706, 664, 636, 541, 527, 516, 501 cm^{-1}.

HRMS $(EI)^+$: *m/z* for $C_{16}H_{10}Br_2$ $[M]^+$: calc.: 359.9144, found: 359.9135.

The title compound was also synthesized on an 8.00 mmol scale following General Procedure 1. The product was obtained as a light-yellow solid (1.25 g, 3.45 mmol, 86%).

(E)-4,4'-(But-1-en-3-yne-1,4-diyl)bis(methylbenzene) (2e)

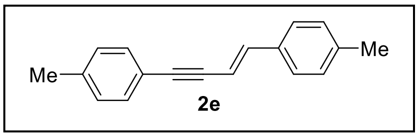

According to General Procedure 1 the title compound was prepared using 1-ethynyl-4-methylbenzene (116 mg, 1.00 mmol, 1.00 equiv.), $[CoBr_2(dppp)]$ (12.6 mg, 20.0 µmol, 0.02 equiv.), zinc dust (2.6 mg, 40.0 µmol, 0.04 equiv.) and triphenylphosphine (11.8 mg, 45.0 µmol, 0.05 equiv.) in acetonitrile (0.8 mL). The reaction mixture was stirred 2 h at 0 °C. The product was obtained after column chromatography (eluent: *n*-pentane:CH_2Cl_2 = 10:1) as a colorless solid (86.2 mg, 371 µmol, 74%).

melting point: 157 – 159 °C.

^{1}H NMR (300 MHz, $CDCl_3$) δ = 7.35 (dd, *J* = 14.8, 7.9 Hz, 4H), 7.15 (dd, *J* = 8.0, 4.1 Hz, 4H), 7.01 (d, *J* = 16.2 Hz, 1H), 6.34 (d, *J* = 16.2 Hz, 1H), 2.37 (s,6H) ppm.

^{13}C NMR (75 MHz, $CDCl_3$) δ = 141.0, 138.7, 138.4, 133.8, 131.5 (2C), 129.6 (2C), 129.2 (2C), 126.3 (2C), 120.6, 107.3, 91.7, 88.6, 21.6, 21.5 ppm.

IR (ATR): $\tilde{\nu}$ = 3029, 2914, 2853, 2190, 1910, 1609, 1580, 1506, 1483, 1283, 1180, 1109, 1072, 1040, 957, 944, 853, 817, 800, 773, 710, 530, 516 cm^{-1}.

HRMS $(EI)^+$: *m/z* for $C_{18}H_{16}$ $[M]^+$: calc.: 232.1247, found: 232.1242.

(E)-4,4'-(But-1-en-3-yne-1,4-diyl)bis(methoxybenzene) (2f)

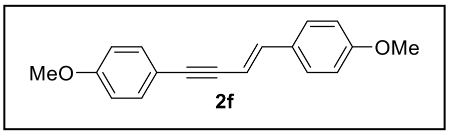

According to General Procedure 1 the title compound was prepared using 1-ethynyl-4-methoxybenzene (132 mg, 1.00 mmol, 1.00 equiv.), $[CoBr_2(dppp)]$ (12.6 mg, 20.0 µmol, 0.02 equiv.), zinc dust (2.6 mg, 40.0 µmol, 0.04 equiv.) and triphenylphosphine (11.8 mg, 45.0 µmol, 0.05 equiv.) in acetonitrile (0.8 mL). The reaction mixture was stirred 2 h at 0 °C. The product was obtained after column chromatography (eluent: *n*-pentane:CH_2Cl_2 = 2:1) as a colorless solid (108 mg, 408 µmol, 82%).

melting point: 153 – 155 °C.

^{1}H NMR (500 MHz, $CDCl_3$) δ = 7.43 – 7.39 (m, 2H), 7.37 – 7.33 (m, 2H), 6.96 (d, *J* = 16.2 Hz, 1H), 6.89 – 6.84 (m, 4H), 6.24 (d, *J* = 16.2 Hz, 1H), 3.82 (s, 3H), 3.82 (s, 3H) ppm.

^{13}C NMR (126 MHz, $CDCl_3$) δ = 160.1, 159.6, 140.2, 133.0 (2C), 129.6, 127.7 (2C), 116.0, 114.3 (2C), 114.2 (2C), 106.2, 91.2, 88.1, 55.5, 55.4 ppm.

IR (ATR): $\tilde{\nu}$ = 2597, 2934, 2834, 2193, 1600, 1566, 1503, 1460, 1439, 1307, 1284, 1246, 1204, 1173, 1107, 1072, 1024, 1009, 964, 936, 851, 833, 821, 807, 769, 670, 640, 563, 539 cm^{-1}.

HRMS $(EI)^+$: *m/z* for $C_{18}H_{16}O_2$ $[M]^+$: calc.: 264.1145, found: 264.1139.

(E)-4,4'-(But-1-en-3-yne-1,4-diyl)bis((trifluoromethyl)benzene) (2g)

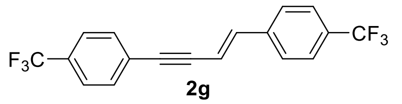

2g

According to General Procedure 1 the title compound was prepared using 1-ethynyl-4-(trifluoromethyl)benzene (170 mg, 1.00 mmol, 1.00 equiv.), $[CoBr_2(dppp)]$ (12.6 mg, 20.0 µmol, 0.02 equiv.), zinc dust (2.6 mg, 40.0 µmol, 0.04 equiv.) and triphenylphosphine (11.8 mg, 45.0 µmol, 0.05 equiv.) in acetonitrile (0.8 mL). The reaction mixture was stirred 2 h at 0 °C. The product was obtained after column chromatography (eluent: *n*-pentane:CH_2Cl_2 = 10:1) as a colorless solid (150 mg, 440 µmol, 88%).

melting point: 138 – 141°C.

^{1}H NMR (500 MHz, $CDCl_3$) δ = 7.63 – 7.56 (m, 6H), 7.53 (d, *J* = 8.2 Hz, 2H), 7.10 (d, *J* = 16.3 Hz, 1H), 6.47 (d, *J* = 16.3 Hz, 1H) ppm.

^{13}C NMR (126 MHz, $CDCl_3$) δ = 140.8, 139.5, 132.0 (2C), 130.8 (q, *J* = 32.8 Hz), 130.3 (q, *J* = 32.7 Hz), 127.1, 126.7 (2C), 126.0 (q, *J* = 3.8 Hz, 2C), 125.5 (q, *J* = 3.6 Hz, 2C), 124.2 (q, *J* = 271.9 Hz), 124.1 (q, *J* = 272.2 Hz), 110.4, 91.7, 90.7 ppm.

^{19}F NMR (470 MHz, $CDCl_3$) δ = □65.8, □66.0 ppm.

IR (ATR): $\tilde{\nu}$ = 2197, 1929, 1612, 1409, 1317, 1174, 1127, 1104, 1064, 1013, 966, 950, 864, 843, 820, 763, 736, 720, 656, 636, 597, 543, 527 cm^{-1}.

HRMS $(EI)^+$: *m/z* for $C_{18}H_{10}F_6$ $[M]^+$: calc.: 340.0681, found: 340.0681.

(E)-4,4'-(But-1-en-3-yne-1,4-diyl)dibenzaldehyde (2h)

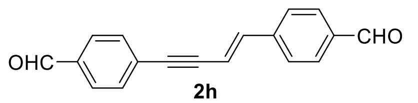

2h

According to General Procedure 1 the title compound was prepared using 4-ethynylbenzaldehyde (130 mg, 1.00 mmol, 1.00 equiv.), $[CoBr_2(dppp)]$ (12.6 mg, 20.0 µmol, 0.02 equiv.), zinc dust (2.6 mg, 40.0 µmol, 0.04 equiv.) and triphenylphosphine (11.8 mg, 45.0 µmol, 0.05 equiv.) in acetonitrile (0.8 mL). The reaction mixture was stirred 2 h at 0 °C. The product was obtained after column chromatography (eluent: CH_2Cl_2) as a light-yellow solid (107 mg, 412 µmol, 82%).

melting point: 204 – 206 °C.

^{1}H NMR (300 MHz, $CDCl_3$) δ = 10.01 (s, 1H), 10.00 (s, 1H), 7.86 (dd, *J* = 8.2, 3.5 Hz, 4H), 7.60 (dd, *J* = 11.4, 8.0 Hz, 4H), 7.13 (d, *J* = 16.2 Hz, 1H), 6.54 (d, *J* = 16.2 Hz, 1H) ppm.

^{13}C NMR (75 MHz, $CDCl_3$) δ = 191.6, 191.5, 141.8, 141.3, 136.4, 135.7, 132.2 (2C), 130.4 (2C), 129.7 (2C), 129.3, 127.0 (2C), 111.2, 92.8, 92.4 ppm.

IR (ATR): $\tilde{\nu}$ = 2849, 2752, 2192, 1926, 1683, 1599, 1560, 1392, 1297, 1286, 107, 1159, 1100, 1009, 947, 866, 857, 827, 801, 721, 640, 570, 531, 514 cm^{-1}.

HRMS $(EI)^+$: *m/z* for $C_{18}H_{18}O_2$ $[M]^+$: calc.: 260.0832, found: 260.0825.

Dimethyl 4,4'-(but-1-en-3-yne-1,4-diyl)(E)-dibenzoate (2i)

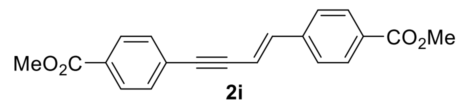

2i

According to General Procedure 1 the title compound was prepared using methyl 4-ethynylbenzoate (160 mg, 1.00 mmol, 1.00 equiv.), [$CoBr_2$(dppp)] (12.6 mg, 20.0 µmol, 0.02 equiv.), zinc dust (2.6 mg, 40.0 µmol, 0.04 equiv.) and triphenylphosphine (11.8 mg, 45.0 µmol, 0.05 equiv.) in acetonitrile (0.8 mL). The reaction mixture was stirred 2 h at 0 °C. The product was obtained after column chromatography (eluent: CH_2Cl_2) as a light-yellow solid (120 mg, 375 µmol, 75%).

melting point: 208 – 210 °C.

^{1}H NMR (300 MHz, $CDCl_3$) δ = 8.01 (dt, *J* = 8.4, 1.9 Hz, 4H), 7.66 – 7.38 (m, 4H), 7.09 (d, *J* = 16.2 Hz, 1H), 6.49 (dd, *J* = 16.2, 1.5 Hz, 1H), 3.92 (s, 6H) ppm.

^{13}C NMR (75 MHz, $CDCl_3$) δ = 166.7, 166.6, 141.2, 140.4, 131.6 (2C), 130.2 (3C), 129.7 (3C), 127.9, 126.4 (2C), 110.4, 92.4, 91.5, 52.4, 52.3 ppm.

IR (ATR): $\tilde{\nu}$ = 2959, 1716, 1603, 1502, 1432, 1410, 1309, 1274, 1190, 1174, 1106, 1016, 949, 861, 830, 814, 763, 696, 560, 531 cm^{-1}.

HRMS $(EI)^+$: *m/z* for $C_{20}H_{16}O_4$ $[M]^+$: calc.: 320.1043, found: 320,1040.

(E)-(But-1-en-3-yne-1,4-diylbis(4,1-phenylene))dimethanol (2j)

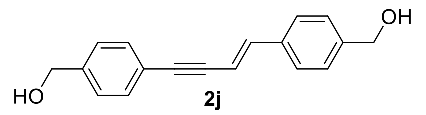

2j

According to General Procedure 1 the title compound was prepared using (4-ethynylphenyl)methanol (132 mg, 1.00 mmol, 1.00 equiv.), [$CoBr_2$(dppp)] (12.6 mg, 20.0 µmol, 0.02 equiv.), zinc dust (2.6 mg, 40.0 µmol, 0.04 equiv.) and triphenylphosphine (11.8 mg, 45.0 µmol, 0.05 equiv.) in acetonitrile (0.8 mL). The reaction mixture was stirred 2 h at 0 °C. The product was obtained after column chromatography (eluent: CH_2Cl_2) as a light-yellow solid (102 mg, 388 µmol, 77%).

melting point: 202 – 205 °C.

^{1}H NMR (300 MHz, DMSO-d_6) δ = 7.53 (d, *J* = 7.8 Hz, 2H), 7.44 (d, *J* = 7.9 Hz, 2H), 7.33 (t, *J* = 8.5 Hz, 4H), 7.08 (d, *J* = 16.3 Hz, 1H), 6.60 (d, *J* = 16.3 Hz, 1H), 5.30 (t, *J* = 5.8 Hz, 1H), 5.24 (t, *J* = 5.8 Hz, 1H), 4.51 (t, *J* = 5.7 Hz, 4H) ppm.

^{13}C NMR (75 MHz, DMSO-d_6) δ = 143.5, 143.3, 141.2, 134.4, 131.0 (2C), 126.8 (2C), 126.7 (2C), 126.3 (2C), 120.8, 107.4, 91.7, 89.1, 62.6, 62.5 ppm.

IR (ATR): $\tilde{\nu}$ = 3262 (bs), 2853, 1694, 1506, 1437, 1410, 1207, 1177, 119, 1036, 1000, 960, 947, 849, 804, 783, 720, 599, 557, 531 cm^{-1}.

HRMS $(EI)^+$: *m/z* for $C_{18}H_{16}O_2$ $[M]^+$: calc.: 264.1145, found: 264.1138.

(E)-4,4'-(But-1-en-3-yne-1,4-diyl)bis(tert-butylbenzene) (2k)

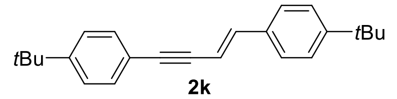

2k

According to General Procedure 1 the title compound was prepared using methyl 1-(*tert*-butyl)-ethynylbenzene (158 mg, 1.00 mmol, 1.00 equiv.), [$CoBr_2$(dppp)] (12.6 mg, 20.0 µmol, 0.02 equiv.), zinc dust (2.6 mg, 40.0 µmol, 0.04 equiv.) and triphenylphosphine (11.8 mg, 45.0 µmol, 0.05 equiv.) in acetonitrile (0.8 mL). The reaction mixture was stirred 2 h at 0 °C. The product was obtained after column chromatography (eluent: *n*-pentane:CH_2Cl_2 = 10:1) as a light yellow solid (146 mg, 462 µmol, 92%).

melting point: 176 – 179°C.

^{1}H NMR (500 MHz, $CDCl_3$) δ = 7.45 – 7.40 (m, 2H), 7.40 – 7.33 (m, 6H), 7.02 (d, *J* = 16.1 Hz, 1H), 6.36 (d, *J* = 16.2 Hz, 1H), 1.34 (s, 9H), 1.33 (s, 9H) ppm.

^{13}C NMR (126 MHz, $CDCl_3$) δ = 152.0, 151.5, 140.9, 133.9, 131.4 (2C), 126.2 (2C), 125.8 (2C), 125.5 (2C), 120.7, 107.7, 91.8, 88.7, 34.9 (2C), 31.4 (3C), 31.3 (3C) ppm.

IR (ATR): $\tilde{\nu}$ = 2953, 2902, 2866, 1502, 1462, 1407, 1392, 1363, 1267, 1203, 1106, 1026, 1014, 957, 943, 853, 834, 814, 760, 836, 697, 559, 523, 513 cm^{-1}.

HRMS $(EI)^+$: *m/z* for $C_{24}H_{28}$ $[M]^+$: calc.: 316.2186, found: 316.2181.

(E)-3,3'-(But-1-en-3-yne-1,4-diyl)bis(methylbenzene) (2l)

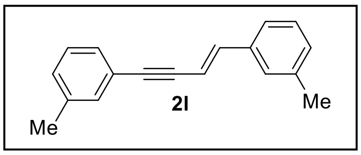

According to General Procedure 1 the title compound was prepared using 1-ethynyl-3-methylbenzene (140 mg, 1.21 mmol, 1.00 equiv.), $[CoBr_2(dppp)]$ (12.6 mg, 20.0 µmol, 0.02 equiv.), zinc dust (2.6 mg, 40.0 µmol, 0.04 equiv.) and triphenylphosphine (11.8 mg, 45.0 µmol, 0.05 equiv.) in acetonitrile (0.8 mL). The reaction mixture was stirred 2 h at 0 °C. The product was obtained after column chromatography (eluent: *n*-pentane:CH_2Cl_2 = 15:1) as a colorless oil (67.5 mg, 291 µmol, 48%).

^{1}H NMR (500 MHz, $CDCl_3$) δ = 7.37 (s, 1H), 7.35 (dd, *J* = 7.6, 1.6 Hz, 1H), 7.32 – 7.27 (m, 4H), 7.21 – 7.14 (m, 2H), 7.06 (d, *J* = 16.2 Hz, 1H), 6.43 (d, *J* = 16.2 Hz, 1H), 2.42 (s, 3H), 2.40 (s, 3H) ppm.

^{13}C NMR (126 MHz, $CDCl_3$) δ = 141.4, 138.5, 138.1, 136.5, 132.2, 129.6, 129.2, 128.8 (2C), 128.4, 127.1, 123.6, 123.5, 108.2, 92.0, 88.8, 21.5, 21.4 ppm.

IR (ATR): $\tilde{\nu}$ = 3024, 2919, 2193, 1942, 1599, 1579, 1482, 1452, 1377, 1297, 1167, 1089, 1040, 949, 904, 876, 774, 687, 521 cm^{-1}.

HRMS $(EI)^+$: *m/z* for $C_{18}H_{16}$ $[M]^+$: calc.: 232.1247 found: 232.1252.

(E)-3,3'-(But-1-en-3-yne-1,4-diyl)bis(methoxybenzene) (2m)

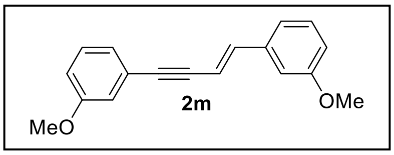

According to General Procedure 1 the title compound was prepared using 1-ethynyl-3-methoxybenzene (132 mg, 1.00 mmol, 1.00 equiv.), $[CoBr_2(dppp)]$ (12.6 mg, 20.0 µmol, 0.02 equiv.), zinc dust (2.6 mg, 40.0 µmol, 0.04 equiv.) and triphenylphosphine (11.8 mg, 45.0 µmol, 0.05 equiv.) in acetonitrile (0.8 mL). The reaction mixture was stirred 2 h at 0 °C. The product was obtained after column chromatography (eluent: *n*-pentane:CH_2Cl_2 = 2:1) as a light-yellow waxy oil (72.2 mg, 273 µmol, 55%).

^{1}H NMR (300 MHz, $CDCl_3$) δ = 7.26 (dd, *J* = 7.7 Hz, 2H), 7.12 – 6.94 (m, 5H), 6.87 (ddd, *J* = 8.8, 7.7, 2.6 Hz, 2H), 6.38 (d, *J* = 16.2 Hz, 1H), 3.83 (s, 3H), 3.82 (s, 3H) ppm.

^{13}C NMR (75 MHz, $CDCl_3$) δ = 160.0, 159.5, 141.4, 137.8, 129.9, 129.5, 124.5, 124.2, 119.2, 116.4, 115.1, 114.4, 111.7, 108.5, 92.0, 88.8, 55.4 (2C) ppm.

IR (ATR): $\tilde{\nu}$ = 2956, 2937, 2833, 2193, 1593, 1573, 1463, 1427, 1320, 1283, 1269, 1243, 1206, 1153, 1080, 1044, 993, 950, 851, 773, 684, 564, 521 cm^{-1}.

HRMS $(EI)^+$: *m/z* for $C_{18}H_{16}O_2$ $[M]^+$: calc.: 264.1145, found: 264.1137.

(E)-3,3'-(But-1-en-3-yne-1,4-diyl)bis(methylbenzene) (2n)

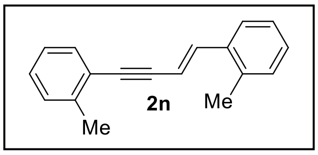

According to General Procedure 1 the title compound was prepared using 1-ethynyl-2-methylbenzene (116 mg, 1.00 mmol, 1.00 equiv.), $[CoBr_2(dppp)]$ (12.6 mg, 20.0 µmol, 0.02 equiv.), zinc dust (2.6 mg, 40.0 µmol, 0.04 equiv.) and triphenylphosphine (11.8 mg, 45.0 µmol, 0.05 equiv.) in acetonitrile (0.8 mL). The reaction mixture was stirred 2 h at 0 °C and

afterwards for 14 h at ambient temperature. The product was obtained after column chromatography (eluent: *n*-pentane:CH_2Cl_2 = 10:1) as a colorless solid (81.4 mg, 350 µmol, 70%).

melting point: 54 – 57 °C.

^{1}H NMR (500 MHz, $CDCl_3$) δ = 7.52 (dd, J = 6.7, 2.3 Hz, 1H), 7.47 (dt, J = 7.5, 1.1 Hz, 1H), 7.29 (d, J = 16.1 Hz, 1H), 7.24 – 7.15 (m, 6H), 6.36 (d, J = 16.1 Hz, 1H), 2.51 (s, 3H), 2.42 (s, 3H) ppm.

^{13}C NMR (126 MHz, $CDCl_3$) δ = 140.2, 138.8, 135.9, 135.5, 132.0, 130.7, 129.6, 128.6, 128.4, 126.4, 125.7, 125.1, 123.4, 109.5, 93.2, 90.5, 20.9, 19.9 ppm.

IR (ATR): $\tilde{\nu}$ = 3062, 3016, 2944, 1953, 1614, 1597, 1476, 1457, 1379, 1290, 1272, 1116, 1102, 1046, 950, 868, 811, 743, 713, 553, 544 cm^{-1}.

HRMS $(EI)^+$: *m/z* for $C_{18}H_{16}$ $[M]^+$: calc.: 232.1247, found: 232.1241.

(E)-2,2'-(But-1-en-3-yne-1,4-diyl)bis(methoxybenzene) (2o)

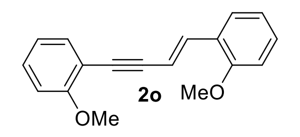

According to General Procedure 1 the title compound was prepared using 1-ethynyl-2-methoxybenzene (132 mg, 1.00 mmol, 1.00 equiv.), $[CoBr_2(dppp)]$ (12.6 mg, 20.0 µmol, 0.02 equiv.), zinc dust (2.6 mg, 40.0 µmol, 0.04 equiv.) and triphenylphosphine (11.8 mg, 45.0 µmol, 0.05 equiv.) in acetonitrile (0.8 mL). The reaction mixture was stirred 2 h at 0 °C and afterwards for 14 h at ambient temperature. The product was obtained after column chromatography (eluent: *n*-pentane:CH_2Cl_2 = 2:1) as a light-yellow waxy oil (94.5 mg, 357 µmol, 72%).

^{1}H NMR (300 MHz, $CDCl_3$) δ = 7.47 (dd, *J* = 7.5, 1.6 Hz, 2H), 7.38 (d, *J* = 16.4 Hz, 1H), 7.33 – 7.23 (m, 2H), 6.99 – 6.85 (m, 4H), 6.55 (d, *J* = 16.4 Hz, 1H), 3.93 (s, 3H), 3.88 (s, 3H) ppm.

^{13}C NMR (75 MHz, $CDCl_3$) δ = 159.9, 157.1, 136.5, 133.6, 129.6 (2C), 127.0, 125.7, 120.8, 120.6, 113.0, 111.1, 110.7, 109.2, 93.9, 87.7, 56.0, 55.6 ppm.

IR (ATR): $\tilde{\nu}$ = 3003, 2936, 2834, 1593, 1572, 1492, 1484, 1463, 1433, 1291, 1272, 1240, 1177, 1162, 1119, 1106, 1049, 1023, 956, 803, 779, 744, 711, 616, 581, 524 cm^{-1}.

HRMS $(EI)^+$: *m/z* for $C_{18}H_{16}O_2$ $[M]^+$: calc.: 264.1145, found: 264.1136.

(E)-3,3'-(But-1-en-3-yne-1,4-diyl)dipyridine (2p)

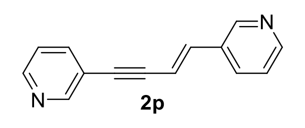

According to General Procedure 1 the title compound was prepared using 3-ethynylpyridine (103 mg, 1.00 mmol, 1.00 equiv.), $[CoBr_2(dppp)]$ (12.6 mg, 20.0 µmol, 0.02 equiv.), zinc dust (2.6 mg, 40.0 µmol, 0.04 equiv.) and triphenylphosphine (11.8 mg, 45.0 µmol, 0.05 equiv.) in acetonitrile (0.8 mL). The reaction mixture was stirred 2 h at 0 °C and then 46 h at ambient temperature. The product was obtained after column chromatography (eluent: ethyl acetate) as a light-brown solid (58.5 mg, 284 µmol, 56%).

melting point: 92 – 95 °C.

^{1}H NMR (300 MHz, $CDCl_3$) δ = 8.71 (d, *J* = 16.6 Hz, 2H), 8.56 (d, *J* = 4.1 Hz, 2H), 7.77 (d, *J* = 7.9 Hz, 2H), 7.30 (dt, *J* = 7.9, 4.8 Hz, 2H), 7.08 (d, *J* = 16.3 Hz, 1H), 6.47 (d, *J* = 16.3 Hz, 1H) ppm.

^{13}C NMR (75 MHz, $CDCl_3$) δ = 152.3, 149.9, 148.8, 148.4, 138.6, 138.5, 132.6, 132.0, 123.9, 123.3, 120.5, 109.9, 91.5, 89.4 ppm.

IR (ATR): $\tilde{\nu}$ = 3056, 3009, 2922, 2852, 1619, 1567, 1559, 1474, 1439, 1420, 1409, 1314, 1269, 1186, 1120, 1020, 963, 951, 855, 847, 814, 800, 754, 720, 699, 653, 626, 539 cm^{-1}.

HRMS $(ESI)^+$: *m/z* for $C_{14}H_{10}N_2H$ [M+H]: calc.: 207.0922, found: 207.0920.

(E)-2,2'-(But-1-en-3-yne-1,4-diyl)dipyridine (2q)

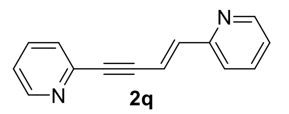

2q

According to General Procedure 1 the title compound was prepared using 2-ethynylpyridine (103 mg, 1.00 mmol, 1.00 equiv.), [$CoBr_2$(dppp)] (12.6 mg, 20.0 µmol, 0.02 equiv.), zinc dust (2.6 mg, 40.0 µmol, 0.04 equiv.) and triphenylphosphine (11.8 mg, 45.0 µmol, 0.05 equiv.) in acetonitrile (0.8 mL). The reaction mixture was stirred 2 h at 0 °C and then 46 h at ambient temperature. The product was obtained after column chromatography (eluent: ethyl acetate) as a light-brown solid (57.4 mg, 278 µmol, 56%).

melting point: 57 – 60 °C.

^{1}H NMR (300 MHz, $CDCl_3$) δ = 8.69 – 8.61 (m, 2H), 7.71 (tt, *J* = 7.7, 2.0 Hz, 2H), 7.53 (dt, *J* = 7.8, 1.1 Hz, 1H), 7.40 – 7.17 (m, 4H), 7.03 (d, *J* = 15.9 Hz, 1H) ppm.

^{13}C NMR (75 MHz, $CDCl_3$) δ = 153.9, 150.2, 150.0, 143.5, 141.9, 136.8, 136.3, 127.4, 123.4, 122.9, 122.8, 111.8, 92.5, 88.5 ppm.

IR (ATR): $\tilde{\nu}$ = 3074, 3052, 3003, 2923, 2852, 2197, 1619, 1577, 1562, 1470, 1460, 1427, 1303, 1260, 1150, 1120, 1094, 1050, 1029, 986, 963, 900, 767, 737, 696, 627, 617, 539 cm^{-1}.

HRMS $(ESI)^+$: *m/z* for $C_{14}H_{10}N_2H$ [M+H]: calc.: 207.0922, found: 207.0922.

(E)-4,4'-(But-1-en-3-yne-1,4-diyl)dithiophene (2r)

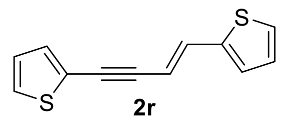

2r

According to General Procedure 1 the title compound was prepared using 2-ethynylthiophene (108 mg, 1.00 mmol, 1.00 equiv.), [$CoBr_2$(dppp)] (12.6 mg, 20.0 µmol, 0.02 equiv.), zinc dust (2.6 mg, 40.0 µmol, 0.04 equiv.) and triphenylphosphine (11.8 mg, 45.0 µmol, 0.05 equiv.) in acetonitrile (0.8 mL). The reaction mixture was stirred 2 h at 0 °C. The product was obtained after column chromatography (eluent: *n*-pentane:CH_2Cl_2 = 10:1) as a yellow solid (56.2 mg, 260 µmol, 52%).

melting point: 102 – 105 °C.

^{1}H NMR (500 MHz, $CDCl_3$) δ = 7.27 (dd, *J* = 5.2, 1.2 Hz, 1H), 7.22 (td, *J* = 4.4, 3.7, 1.1 Hz, 2H), 7.12 (d, *J* = 15.9 Hz, 1H), 7.06 (d, *J* = 3.4 Hz, 1H), 7.00 (td, *J* = 5.2, 3.6 Hz, 2H), 6.19 (d, *J* = 15.9 Hz, 1H) ppm.

^{13}C NMR (126 MHz, $CDCl_3$) δ = 141.5, 134.1, 131.9, 127.9, 127.4, 127.4, 127.3, 125.8, 123.6, 107.0, 92.7, 85.4 ppm.

IR (ATR): $\tilde{\nu}$ = 3102, 3074, 3040, 2184, 1800, 1652, 1602, 1503, 1419, 1359, 1277, 1489, 1217, 1202, 1194, 1041, 907, 934, 846, 830, 809, 757, 694, 577, 540, 531, 524, 514, 507 cm^{-1}.

HRMS $(EI)^+$: *m/z* for $C_{12}H_8S_2$ $[M]^+$: calc.: 216.0062, found: 216.0058.

(E)-2,7-Dimethylocta-1,3,7-trien-5-yne (2s)

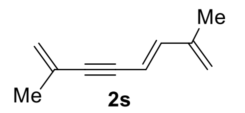

2s

According to General Procedure 1 the title compound was prepared using 2-methylbut-1-en-3-yne (95.2 µL, 1.00 mmol, 1.00 equiv.), [$CoBr_2$(dppp)] (12.6 mg, 20.0 µmol, 0.02 equiv.), zinc dust (2.6 mg, 40.0 µmol, 0.04 equiv.) and triphenylphosphine (11.8 mg, 45.0 µmol, 0.05 equiv.) in acetonitrile (0.8 mL). The reaction mixture was stirred 2 h at 0 °C. The product was obtained after column chromatography (eluent: *n*-pentane) as a colorless liquid (19.0 mg, 143 µmol, 29%).

^{1}H NMR (500 MHz, $CDCl_3$) δ = 6.69 (d, *J* = 16.1 Hz, 1H), 5.71 (d, *J* = 16.0 Hz, 1H), 5.32 – 5.27 (m, 1H), 5.23 (p, *J* = 1.6 Hz, 1H), 5.09 – 5.04 (m, 2H), 1.95 – 1.90 (m, 3H), 1.85 – 1.85 (m, 3H) ppm.

^{13}C NMR (126 MHz, $CDCl_3$) δ = 144.2, 141.6, 127.2, 121.7, 119.0, 108.4, 93.1, 88.0, 23.6, 18.0 ppm.

IR (ATR): $\tilde{\nu}$ = 2976, 2952, 2923, 1793, 1610, 18586, 1454, 1373, 1297, 1215, 954, 907, 840, 757, 733, 669, 650, 530 cm^{-1}.

HRMS $(EI)^+$: *m/z* for $C_{10}H_{12}$ $[M]^+$: calc.: 132.0934, found: 132.0934.

Synthesis of (*Z*)-But-1-en-3-ynes

General Procedure 2:

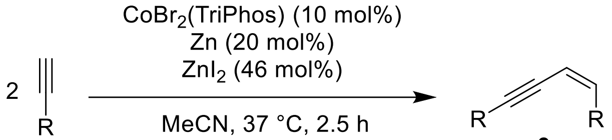

3

Under Argon atmosphere [$CoBr_2$(TriPhos)] (10 mol%), zinc dust (20 mol%) and zinc iodide (46 mol%) were added to a pre-dried reaction vessel. The solids were dried 10 min *in vacuo*. The catalyst system was dissolved in dry acetonitrile (0.67 mL·mmol^{-1}) and the resulting dark red suspension was heated to 37 °C and stirred for 15 min. Then the corresponding alkyne (1.00 equiv.) was added at 37 °C. The reaction mixture was stirred until complete conversion was determined via GC/MS and TLC. The mixture was diluted with CH_2Cl_2 and filtered over a short plug of silica gel (eluent: *n*-pentane:CH_2Cl_2 = 3:1 or CH_2Cl_2, depending on the polarity of the substrate). The solvent was removed under reduced pressure. The crude product was purified via column chromatography (*n*-pentane:CH_2Cl_2 or *n*-pentane:ethyl acetate).

(Z)-But-1-en-3-yne-1,4-diylbenzene (3a)

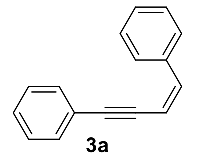

3a

According to General Procedure 2 the title compound was prepared using phenylacetylene (102 mg, 1.00 mmol, 1.00 equiv.), [$CoBr_2$(TriPhos)] (75.3 mg, 100 µmol, 0.10 equiv.), zinc dust (13.1 mg, 200 µmol, 0.20 equiv.) and zinc iodide (147 mg, 460 µmol, 0.46 equiv.) in acetonitrile (0.67 mL). The reaction mixture was stirred 2.5 h at 37 °C. The product was obtained after column chromatography (eluent: *n*-pentane:CH_2Cl_2 = 10:1) as a colorless liquid (65.6 mg, 321 µmol, 64%, *E:Z* = 6:94).

^{1}H NMR (500 MHz, $CDCl_3$) δ = 7.95 (dd, *J* = 8.3, 1.4 Hz, 2H), 7.54 – 7.49 (m, 2H), 7.43 – 7.39 (m, 2H), 7.38 – 7.31 (m, 4H), 6.72 (d, *J* = 11.9 Hz, 1H), 5.94 (d, *J* = 11.9 Hz, 1H) ppm.

^{13}C NMR (126 MHz, $CDCl_3$) δ =138.8, 136.7, 131.6 (2C), 128.9 (2C), 128.6 (3C), 128.5, 128.4 (2C), 123.7, 107.6, 96.0, 88.4 ppm.

IR (ATR): $\tilde{\nu}$ = 3060, 3020, 2190, 1949, 1880, 1594, 1569, 1489, 1442, 1410, 1184, 1157, 1070, 1027, 953, 914, 834, 780, 686, 563, 523 cm^{-1}.

HRMS (EI)$^+$: *m/z* for $C_{16}H_{12}$ [M]$^+$: calc.: 204.0934, found: 204.0933.

(Z)-4,4'-(But-1-en-3-yne-1,4-diyl)bis(fluorobenzene) (3b)

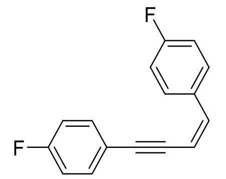

3b

According to General Procedure 2 the title compound was prepared using 4-fluorophenylacetylene (120 mg, 1.00 mmol, 1.00 equiv.), [$CoBr_2$(TriPhos)] (75.3 mg, 100 µmol, 0.10 equiv.), zinc dust (13.1 mg, 200 µmol, 0.20 equiv.) and zinc iodide (147 mg, 460 µmol, 0.46 equiv.) in acetonitrile (0.67 mL). The reaction mixture was stirred 2.5 h at 37 °C. The product was obtained after column chromatography (eluent: *n*-pentane:CH_2Cl_2 = 10:1) as a colorless liquid (78.0 mg, 325 µmol, 65%, *E:Z* = 5:95).

^{1}H NMR (500 MHz, $CDCl_3$) δ = 7.93 – 7.85 (m, 2H), 7.45 (ddd, *J* = 8.3, 5.3, 2.6 Hz, 2H), 7.09 – 7.03 (m, 4H), 6.67 (d, *J* = 11.9 Hz, 1H), 5.88 (d, *J* = 11.9 Hz, 1H) ppm.

^{13}C NMR (126 MHz, $CDCl_3$) δ = 162.8 (d, *J* = 250.3 Hz), 162.7 (d, *J* = 249.3 Hz), 137.6, 133.5 (d, *J* = 8.3 Hz, 2C), 132.9 (d, *J* = 3.4 Hz, 2C), 130.6 (d, *J* = 8.1 Hz, 2C), 119.6 (d, *J* = 3.6 Hz, 2C), 116.0 (d, *J* = 22.2 Hz), 115.4 (d, *J* = 21.6 Hz), 107.0 (d, *J* = 1.8 Hz), 94.8, 87.8 ppm.

IR (ATR): $\tilde{\nu}$ = 3023, 2360, 2332, 2192, 1890, 1599, 1582, 1503, 1422, 1402, 1313, 1296, 1229, 1173, 1154, 1093, 1013, 977, 954, 937, 830, 814, 787, 754, 669, 640, 609, 560, 526 cm^{-1}.

HRMS (EI)$^+$: *m/z* for $C_{16}H_{10}F_2$ [M]$^+$: calc.: 240.0745, found: 240.0752.

General Procedure 3:

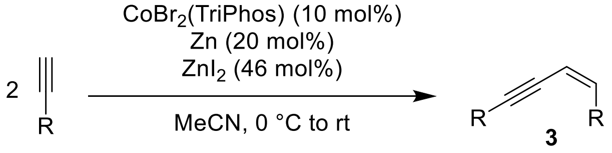

Under Argon atmosphere [$CoBr_2$(TriPhos)] (10 mol%), zinc dust (20 mol%) and zinc iodide (46 mol%) were added to a pre-dried reaction vessel. The solids were dried 10 min *in vacuo*. The catalyst system was dissolved in dry acetonitrile (0.67 mL·mmol^{-1}) and the resulting dark red suspension was heated to 37 °C and stirred for 15 min. Then the corresponding alkyne (1.00 equiv.) was added at 0 °C. The reaction mixture was stirred 1 h at 0 °C. Afterwards the mixture was stirred at ambient temperature until complete conversion was determined via GC/MS and TLC. The mixture was diluted with CH_2Cl_2 and filtered over a short plug of silica gel (eluent: *n*-pentane:CH_2Cl_2 = 3:1 or CH_2Cl_2, depending on the polarity of the substrate). The solvent was removed under reduced pressure. The crude product was purified via column chromatography (*n*-pentane:CH_2Cl_2 or *n*-pentane:ethyl acetate).

(Z)-4,4'-(But-1-en-3-yne-1,4-diyl)bis(chlorobenzene) (3c)

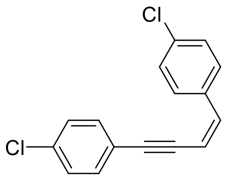

3c

According to General Procedure 3 the title compound was prepared using 4-chlorophenylacetylene (136 mg, 1.00 mmol, 1.00 equiv.), [$CoBr_2$(TriPhos)] (75.3 mg, 100 µmol, 0.10 equiv.), zinc dust (13.1 mg, 200 µmol, 0.20 equiv.) and zinc iodide (147 mg, 460 µmol, 0.46 equiv.) in acetonitrile (0.67 mL). The alkyne was added at 0 °C, the mixture was warmed to room temperature and stirred for 4 h. The product was obtained after column chromatography (eluent: *n*-pentane:CH_2Cl_2 = 10:1) as a light-yellow solid (94.1 mg, 345 µmol, 69%, *E:Z* < 1:99).

melting point: 99 – 101 °C.

^{1}H NMR (500 MHz, $CDCl_3$) δ = 7.83 (d, *J* = 8.4 Hz, 2H), 7.46 – 7.29 (m, 6H), 6.67 (d, *J* = 11.9 Hz, 1H), 5.92 (d, *J* = 11.9 Hz, 1H) ppm.

^{13}C NMR (126 MHz, $CDCl_3$) δ = 137.8, 135.0, 134.8, 134.3, 132.8 (2C), 130.1 (2C), 129.0 (2C), 128.7 (2C), 121.8, 107.9, 95.4, 88.9 ppm.

IR (ATR): $\tilde{\nu}$ = 3017, 2187, 1896, 1587, 1559, 1486, 1422, 1343, 1313, 1269, 1223, 1113, 1090, 1013, 967, 956, 834, 823, 813, 759, 851, 726, 703, 686, 637, 604, 556, 534, 519 cm^{-1}.

HRMS (EI)$^+$: *m/z* for $C_{16}H_{10}Cl_2$ [M]$^+$: calc.: 272.0154, found: 272.0154.

(Z)-4,4'-(But-1-en-3-yne-1,4-diyl)bis(bromobenzene) (3d)

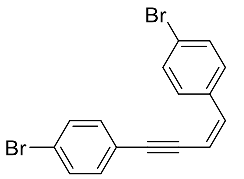

3d

According to General Procedure 3 the title compound was prepared using 4-bromophenylacetylene (136 mg, 1.00 mmol, 1.00 equiv.), [$CoBr_2$(TriPhos)] (75.3 mg, 100 µmol, 0.10 equiv.), zinc dust (13.1 mg, 200 µmol, 0.20 equiv.) and zinc iodide (147 mg, 460 µmol, 0.46 equiv.) in acetonitrile (0.67 mL). The alkyne was added at 0 °C, the mixture was warmed to room temperature and stirred for 4 h. The product was obtained after column chromatography (eluent: *n*-pentane:CH_2Cl_2 = 10:1) as a light-yellow solid (114 mg, 315 µmol, 63%, *E:Z* = 1:99).

melting point: 106 – 109 °C.

^{1}H NMR (500 MHz, $CDCl_3$) δ = 8.80 – 8.72 (m, 2H), 8.54 – 8.46 (m, 4H), 8.36 – 8.30 (m, 2H), 7.66 (d, *J* = 11.9 Hz, 1H), 6.93 (d, *J* = 11.9 Hz, 1H) ppm.

^{13}C NMR (126 MHz, $CDCl_3$) δ = 137.9, 135.4, 133.0 (2C), 131.9 (2C), 131.6 (2C), 130.3 (2C), 123.0, 122.6, 122.3, 108.1, 95.5, 89.1 ppm.

IR (ATR): $\tilde{\nu}$ = 3014, 2186, 1894, 1582, 1557, 1482, 1420, 1392, 1313, 1269, 1176, 1110, 1094, 1070, 1009, 977, 967, 956, 834, 823, 811, 747, 701, 693, 636, 627, 556, 517 cm^{-1}.

HRMS $(EI)^+$: *m/z* for $C_{16}H_{10}Br_2$ $[M]^+$: calc.: 359.9144, found: 359.9139.

(Z)-4,4'-(But-1-en-3-yne-1,4-diyl)bis(methylbenzene) (3e)

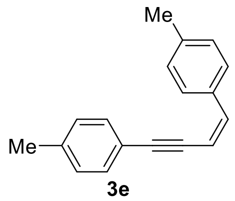

3e

According to General Procedure 3 the title compound was prepared using 4-methylphenylacetylene (116 mg, 1.00 mmol, 1.00 equiv.), [$CoBr_2$(TriPhos)] (75.3 mg, 100 µmol, 0.10 equiv.), zinc dust (13.1 mg, 200 µmol, 0.20 equiv.) and zinc iodide (147 mg, 460 µmol, 0.46 equiv.) in acetonitrile (0.67 mL). The alkyne was added at 0 °C, the mixture was warmed to room temperature and stirred for 4 h. The product was obtained after column chromatography (eluent: *n*-pentane:CH_2Cl_2 = 10:1) as a colorless solid (78.8 mg, 339 µmol, 68%, *E:Z* = 5:95).

melting point: 65 – 67 °C.

^{1}H NMR (500 MHz, $CDCl_3$) δ = 7.85 (d, *J* = 8.2 Hz, 2H), 7.40 (d, *J* = 8.1 Hz, 2H), 7.21 (d, *J* = 8.0 Hz, 2H), 7.17 (d, *J* = 7.8 Hz, 2H), 6.66 (d, *J* = 11.8 Hz, 1H), 5.87 (d, *J* = 11.9 Hz, 1H), 2.39 (s, 6H) ppm.

^{13}C NMR (126 MHz, $CDCl_3$) δ = 138.6, 138.4, 134.1, 131.5 (2C), 129.3, 129.2 (2C), 129.1 (2C), 128.9 (2C), 120.7, 106.7, 96.1, 88.1, 21.7, 21.5 ppm.

IR (ATR): $\tilde{\nu}$ = 3016, 2914, 2184, 1896, 1623, 1582, 1556, 1506, 1480, 1392, 1269, 1172, 1107, 1070, 1037, 1009, 964, 834, 823, 811, 749, 706, 646, 560, 517 cm^{-1}.

HRMS $(EI)^+$: *m/z* for $C_{18}H_{16}$ $[M]^+$: calc.: 232.1247, found: 232.1244.

(Z)-4,4'-(But-1-en-3-yne-1,4-diyl)bis(methoxybenzene) (3f)

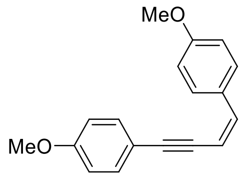

3f

According to General Procedure 3 the title compound was prepared using 4-methoxyphenylacetylene (132 mg, 1.00 mmol, 1.00 equiv.), [$CoBr_2$(TriPhos)] (75.3 mg, 100 µmol, 0.10 equiv.), zinc dust (13.1 mg, 200 µmol, 0.20 equiv.) and zinc iodide (147 mg, 460 µmol, 0.46 equiv.) in acetonitrile (0.67 mL). The alkyne was added at 0 °C, the mixture was warmed to room temperature and stirred for 4 h. The product was obtained after column chromatography (eluent: *n*-pentane:CH_2Cl_2 = 2:1) as a light-yellow waxy oil (89.0 mg, 336 µmol, 68%, *E:Z* = 3:97).

^{1}H NMR (500 MHz, $CDCl_3$) δ = 7.93 – 7.86 (m, 2H), 7.46 – 7.40 (m, 2H), 6.93 – 6.87 (m, 4H), 6.60 (d, *J* = 11.9 Hz, 1H), 5.79 (d, *J* = 11.9 Hz, 1H), 3.84 (s, 3H), 3.83 (s, 3H) ppm.

^{13}C NMR (126 MHz, $CDCl_3$) δ = 159.8 (2C), 137.5, 133.0 (2C), 130.3 (2C), 130.0, 116.0, 114.3 (2C), 113.8 (2C), 105.4, 95.6, 87.6, 55.5 (2C) ppm.

IR (ATR): $\tilde{\nu}$ = 3007, 2967, 2930, 2836, 2183, 2049, 1892, 1717, 1603, 1563, 1503, 1460, 1437, 1409, 1306, 1289, 1259, 1243, 1106, 1027, 959, 853, 830, 813, 781, 760, 714, 686, 647, 636, 610, 561, 533, 516 cm^{-1}.

HRMS $(EI)^+$: *m/z* for $C_{18}H_{16}O_2$ $[M]^+$: calc.: 264.1145, found: 264.1144.

(Z)-4,4'-(But-1-en-3-yne-1,4-diyl)bis(trifluoromethylbenzene) (3g)

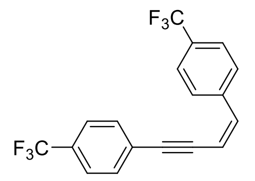
3g

According to General Procedure 3 the title compound was prepared using 4-trifluoromethylphenyl-acetylene (132 mg, 1.00 mmol, 1.00 equiv.), [$CoBr_2$(TriPhos)] (75.3 mg, 100 µmol, 0.10 equiv.), zinc dust (13.1 mg, 200 µmol, 0.20 equiv.) and zinc iodide (147 mg, 460 µmol, 0.46 equiv.) in acetonitrile (0.67 mL). The alkyne was added at 0 °C and the mixture was warmed to room temperature and stirred for 4 h. The product was obtained after column chromatography (eluent: *n*-pentane:CH_2Cl_2 = 10:1) as a colorless oil (91.2 mg, 268 µmol, 54%, *E:Z* = 3:97).

^{1}H NMR (500 MHz, $CDCl_3$) δ = 7.99 (d, *J* = 8.1 Hz, 2H), 7.68 – 7.61 (m, 4H), 7.58 (d, *J* = 8.2 Hz, 2H), 6.80 (d, *J* = 12.0 Hz, 1H), 6.06 (d, *J* = 11.9 Hz, 1H) ppm.

^{13}C NMR (126 MHz, $CDCl_3$) δ = 139.7, 138.3, 131.9, 130.6 (q, *J* = 32.9 Hz, 2C), 130.51 (q, *J* = 32.6 Hz, 2C), 129.0, 126.9, 125.6 (q, *J* = 3.6 Hz, 2C), 125.1 (q, *J* = 272.1 Hz), 125.5 (q, *J* = 3.6 Hz, 2C), 124.2 (q, *J* = 272.2 Hz), 109.6, 95.3, 89.8.ppm.

^{19}F NMR (470 MHz, $CDCl_3$) δ = −62.7, −62.9 ppm.

IR (ATR): $\tilde{\nu}$ = 2306, 1922, 1799, 1682, 1612, 1569, 1427, 1404, 1314, 1157, 1103, 1061, 1013, 837, 766, 721, 691, 596, 570, 520 cm^{-1}.

HRMS $(EI)^+$: *m/z* for $C_{18}H_{10}F_6$ $[M]^+$: calc.: 340.0681, found: 340.0675.

(Z)-4,4'-(But-1-en-3-yne-1,4-diyl)bis(methylbenzene) (3h)

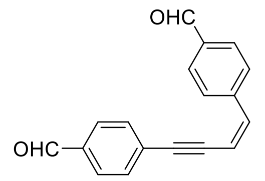
3h

According to General Procedure 3 the title compound was prepared using 4-ethynylbenzaldehyde (130 mg, 1.00 mmol, 1.00 equiv.), [$CoBr_2$(TriPhos)] (75.3 mg, 100 µmol, 0.10 equiv.), zinc dust (13.1 mg, 200 µmol, 0.20 equiv.) and zinc iodide (147 mg, 460 µmol, 0.46 equiv.) in acetonitrile (0.67 mL). The alkyne was added at 0 °C, the mixture was warmed to 37 °C and stirred for 24 h. The product was obtained after column chromatography (eluent: CH_2Cl_2) as a light-yellow solid (17.9 mg, 68.7 µmol, 14%, *E:Z* = 9:91).

melting point: 106 – 110 °C.

^{1}H NMR (300 MHz, $CDCl_3$) δ = 10.03 (s, 2H), 8.04 (d, *J* = 8.2 Hz, 2H), 7.93 – 7.86 (m, 4H), 7.62 (d, *J* = 8.1 Hz, 2H), 6.84 (d, *J* = 12.0 Hz, 1H), 6.11 (d, *J* = 11.9 Hz, 1H) ppm.

^{13}C NMR (75 MHz, $CDCl_3$) δ = 191.7, 191.4, 142.1, 138.7, 136.1, 135.9, 132.2 (2C), 129.9 (2C), 129.8 (2C), 129.4 (2C), 129.2, 110.4, 96.3, 91.5 ppm.

IR (ATR): $\tilde{\nu}$ = 3359, 2722, 1924, 1804, 1684, 1597, 1559, 1503, 1429, 1389, 1302, 1207, 1162, 1103, 1011, 949, 824, 807, 724, 710, 686, 639, 579, 561, 547, 531, 523 cm^{-1}.

HRMS $(EI)^+$: *m/z* for $C_{18}H_{12}O_2$ $[M]^+$: calc.: 260.0832, found: 260.0831.

(Z)-4,4'-(But-1-en-3-yne-1,4-diyl)bis(tert-butylbenzene) (3i)

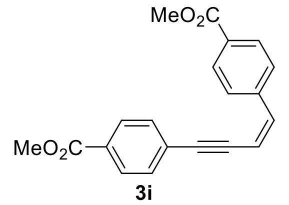
3i

According to General Procedure 3 the title compound was prepared using methyl 4-ethynyl benzoate (160 mg, 1.00 mmol, 1.00 equiv.), [$CoBr_2$(TriPhos)] (75.3 mg, 100 µmol, 0.10 equiv.), zinc dust (13.1 mg, 200 µmol, 0.20 equiv.) and zinc iodide (147 mg, 460 µmol, 0.46 equiv.) in acetonitrile (0.67 mL). The alkyne was added at 0 °C, the mixture was warmed to room temperature and stirred for 4 h. The product was obtained after column chromatography (eluent: CH_2Cl_2) as a yellow waxy oil (108 mg, 337 µmol, 67%, *E:Z* = 2:98).

^{1}H NMR (500 MHz, $CDCl_3$) δ = 8.08 – 8.04 (m, 2H), 8.04 – 8.02 (m, 2H), 7.95 (d, *J* = 8.4 Hz, 2H), 7.57 – 7.50 (m, 2H), 6.79 (d, *J* = 11.9 Hz, 1H), 6.05 (d, *J* = 11.9 Hz, 1H), 3.93 (s, 3H), 3.93 (s, 3H) ppm.

^{13}C NMR (126 MHz, $CDCl_3$) δ = 166.8, 166.6, 140.7, 138.7 (2C), 131.6 (2C), 130.0, 129.8 (4C), 128.8 (2C), 127.8, 109.6, 96.1, 90.7, 52.4, 52.3 ppm.

IR (ATR): $\tilde{\nu}$ = 3039, 3019, 2964, 1719, 1604, 1559, 1504, 1467, 1436, 1404, 1312, 1273, 1183, 1104, 1014, 954, 854, 840, 810, 781, 763, 739, 689, 519 cm^{-1}.

HRMS $(EI)^+$: *m/z* for $C_{20}H_{16}O_4$ $[M]^+$: calc.: 320.1043, found: 320.1034.

(Z)-4,4'-(But-1-en-3-yne-1,4-diyl)bis(tert-butylbenzene) (3k)

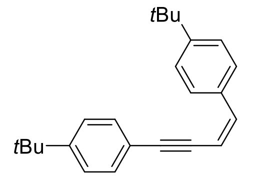

3k

According to General Procedure 3 the title compound was prepared using 4-*tert*-butylphenylacetylene (158 mg, 1.00 mmol, 1.00 equiv.), $[CoBr_2(TriPhos)]$ (75.3 mg, 100 µmol, 0.10 equiv.), zinc dust (13.1 mg, 200 µmol, 0.20 equiv.) and zinc iodide (147 mg, 460 µmol, 0.46 equiv.) in acetonitrile (0.67 mL). The alkyne was added at 0 °C, the mixture was warmed to room temperature and stirred for 4 h. The product was obtained after column chromatography (eluent: *n*-pentane:CH_2Cl_2 = 10:1) as a yellow waxy oil (94.6 mg, 299 µmol, 60%, *E:Z* = 1:99).

^{1}H NMR (500 MHz, $CDCl_3$) δ = 7.94 – 7.87 (m, 2H), 7.48 – 7.44 (m, 2H), 7.45 – 7.37 (m, 4H), 6.66 (d, *J* = 11.9 Hz, 1H), 5.88 (d, *J* = 11.9 Hz, 1H), 1.36 (s, 9H), 1.35 (s, 9H) ppm.

^{13}C NMR (126 MHz, $CDCl_3$) δ = 151.8, 151.7, 138.2, 134.1, 131.3 (2C), 128.7 (2C), 125.6 (2C), 125.4 (2C), 120.8, 106.8, 96.1, 88.1, 35.0, 34.9, 31.4 (3C), 31.3 (3C) ppm.

IR (ATR): $\tilde{\nu}$ = 3020, 2960, 2903, 2867, 2190, 1909, 1787, 1669, 1606, 1503, 1462, 1420, 1394, 1363, 1322, 1267, 1230, 1200, 1107, 1017, 923, 833, 763, 726, 577, 559, 523, 513 cm^{-1}.

HRMS $(EI)^+$: *m/z* for $C_{24}H_{28}$ $[M]^+$: calc.: 316.2186, found: 316.2181.

(Z)-3,3'-(But-1-en-3-yne-1,4-diyl)bis(methylbenzene) (3l)

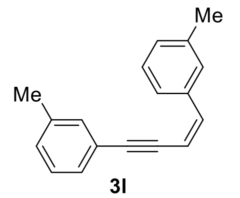

3l

According to General Procedure 3 the title compound was prepared using 3-methylphenylacetylene (116 mg, 1.00 mmol, 1.00 equiv.), $[CoBr_2(TriPhos)]$ (75.3 mg, 100 µmol, 0.10 equiv.), zinc dust (13.1 mg, 200 µmol, 0.20 equiv.) and zinc iodide (147 mg, 460 µmol, 0.46 equiv.) in acetonitrile (0.67 mL). The alkyne was added at 0 °C, the mixture was warmed to room temperature and stirred for 24 h. The product was obtained after column chromatography (eluent: *n*-pentane:CH_2Cl_2 = 15:1) as a yellow oil (64.7 mg, 279 µmol, 56%, *E:Z* = 4:96).

^{1}H NMR (500 MHz, $CDCl_3$) δ =7.83 (d, *J* = 2.0 Hz, 1H), 7.72 (dt, *J* = 7.7, 1.3 Hz, 1H), 7.35 – 7.27 (m, 3H), 7.25 (d, *J* = 7.6 Hz, 1H), 7.19 – 7.13 (m, 2H), 6.68 (d, *J* = 11.9 Hz, 1H), 5.91 (d, *J* = 11.9 Hz, 1H), 2.41 (s, 3H), 2.37 (s, 3H) ppm.

^{13}C NMR (126 MHz, $CDCl_3$) δ = 138.8, 138.2, 137.9, 136.7, 132.2, 129.6, 129.4 (2C), 128.7, 128.5, 128.3, 126.2, 123.6, 107.4, 96.2, 88.3, 21.6, 21.4 ppm.

IR (ATR): $\tilde{\nu}$ = 3019, 2919, 2860, 2732, 2187, 1942, 1697, 1599, 1577, 1483, 1447, 1377, 1300, 1283, 1144, 1090, 1039, 953, 904, 879, 797, 780, 687, 586, 564, 523 cm^{-1}.

HRMS $(EI)^+$: *m/z* for $C_{18}H_{16}$ $[M]^+$: calc.: 232.1247, found: 232.1242.

(Z)-3,3'-(But-1-en-3-yne-1,4-diyl)bis(methoxybenzene) (3m)

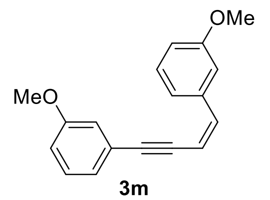

3m

According to General Procedure 3 the title compound was prepared using 3-methoxyphenyl-acetylene (132 mg, 1.00 mmol, 1.00 equiv.), [$CoBr_2$(TriPhos)] (75.3 mg, 100 µmol, 0.10 equiv.), zinc dust (13.1 mg, 200 µmol, 0.20 equiv.) and zinc iodide (147 mg, 460 µmol, 0.46 equiv.) in acetonitrile (0.67 mL). The alkyne was added at 0 °C, the mixture was warmed to room temperature and stirred for 4 h. The product was obtained after column chromatography (eluent: *n*-pentane:CH_2Cl_2 = 2:1) as a colorless oil (89.5 mg, 339 µmol, 68%, *E:Z* = 1:99).

^{1}H NMR (500 MHz, $CDCl_3$) δ = 7.68 (t, *J* = 2.0 Hz, 1H), 7.39 (dt, *J* = 7.5, 1.2 Hz, 1H), 7.30 (t, *J* = 7.9 Hz, 1H), 7.26 (t, *J* = 7.9 Hz, 1H), 7.10 (dt, *J* = 7.6, 1.2 Hz, 1H), 7.04 (dd, *J* = 2.6, 1.3 Hz, 1H), 6.93 – 6.86 (m, 2H), 6.69 (d, *J* = 12.0 Hz, 1H), 5.93 (d, *J* = 11.9 Hz, 1H), 3.82 (s, 6H) ppm.

^{13}C NMR (126 MHz, $CDCl_3$) δ = 159.6, 159.5, 138.8, 137.9, 129.6, 129.4, 124.5, 124.1, 121.9, 116.3, 115.2, 114.9, 113.4, 107.7, 96.3, 88.2, 55.4 (2C) ppm.

IR (ATR): $\tilde{\nu}$ = 3002, 2956, 2937, 2833, 2189, 1929, 1843, 1706, 1594, 1572, 1484, 1460, 1429, 1317, 1289, 1257, 1236, 1203, 1162, 1082, 1040, 991, 854, 779, 706, 684, 564, 523 cm^{-1}.

HRMS $(EI)^+$: *m/z* for $C_{18}H_{16}O_2$ $[M]^+$: calc.: 264.1145, found: 264.1145.

(Z)-2,2'-(But-1-en-3-yne-1,4-diyl)bis(methylbenzene) (3n)

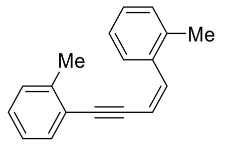

3n

According to General Procedure 3 the title compound was prepared using 2-methylphenylacetylene (116 mg, 1.00 mmol, 1.00 equiv.), [$CoBr_2$(TriPhos)] (75.3 mg, 100 µmol, 0.10 equiv.), zinc dust (13.1 mg, 200 µmol, 0.20 equiv.) and zinc iodide (147 mg, 460 µmol, 0.46 equiv.) in acetonitrile (0.67 mL). The alkyne was added at 0 °C, the mixture was warmed to room temperature and stirred for 24 h. The product was obtained after column chromatography (eluent: *n*-pentane:CH_2Cl_2 = 10:1) as a yellow oil (53.0 mg, 228 µmol, 46%, *E:Z* = 41:59).

^{1}H NMR (500 MHz, $CDCl_3$) δ = 8.32 (dd, *J* = 5.0, 3.8 Hz, 1H), 7.45 (dd, *J* = 7.5, 1.2 Hz, 1H), 7.29 – 7.23 (m, 6H), 6.96 (d, *J* = 11.7 Hz, 1H), 6.09 (d, *J* = 11.9 Hz, 1H), 2.47 (s, 3H), 2.43 (s, 3H) ppm.

^{13}C NMR (126 MHz, $CDCl_3$) δ = 140.3, 136.7, 136.5, 135.5, 132.2, 130.2, 129.6, 128.5, 128.4 (2C), 125.7 (2C), 123.5, 108.7, 94.1, 91.8, 20.9, 19.9 ppm.

IR (ATR): $\tilde{\nu}$ = 3019, 2946, 2920, 2735, 2322, 2187, 1952, 1916, 1804, 1694, 1599, 1483, 1456, 1379, 1197, 1159, 1114, 1044, 987, 950, 866, 839, 811, 790, 750, 711, 667, 623, 514 cm^{-1}.

HRMS $(EI)^+$: *m/z* for $C_{18}H_{16}$ $[M]^+$: calc.: 232.1247, found: 232.1245.

(Z)-2,2'-(But-1-en-3-yne-1,4-diyl)bis(methoxybenzene) (3o)

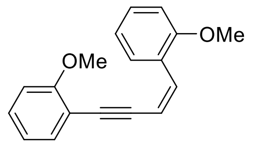

3o

According to General Procedure 3 the title compound was prepared using 2-methoxyphenyl-acetylene (132 mg, 1.00 mmol, 1.00 equiv.), [$CoBr_2$(TriPhos)] (75.3 mg, 100 µmol, 0.10 equiv.), zinc dust (13.1 mg, 200 µmol, 0.20 equiv.) and zinc iodide (147 mg, 460 µmol, 0.46 equiv.) in acetonitrile (0.67 mL). The alkyne was added at 0 °C, the mixture was warmed to room temperature and stirred for 18 h. The product was obtained after column chromatography (eluent: *n*-pentane:CH_2Cl_2 = 2:1) as a light-yellow waxy oil (81.4 mg, 308 µmol, 62%, *E:Z* = 30:70).

^{1}H NMR (500 MHz, $CDCl_3$) δ = 8.70 (dd, *J* = 7.8, 1.7 Hz, 1H), 7.42 (dd, *J* = 7.6, 1.7 Hz, 1H), 7.33 – 7.25 (m, 2H), 7.13 (d, *J* = 12.2 Hz, 1H), 7.05 – 6.97 (m, 1H), 6.96 – 6.89 (m, 1H), 6.89 (d, *J* = 8.3 Hz, 2H), 5.99 (d, *J* = 12.1 Hz, 1H), 3.93 (s, 3H), 3.86 (s, 3H) ppm.

^{13}C NMR (126 MHz, $CDCl_3$) δ = 160.3, 157.1, 133.4, 132.3, 129.8, 129.7, 129.0, 125.8, 120.6, 120.3, 113.1, 110.8, 110.6, 107.2, 92.8, 92.3, 55.9, 55.7 ppm.

IR (ATR): $\tilde{\nu}$ = 3003, 2960, 2936, 2834, 2186, 1594, 1572, 1487, 1462, 1433, 1316, 1292, 1269, 1240, 1179, 1162, 1119, 1107, 1047, 1023, 957, 934, 853, 831, 801, 746, 671, 580 cm^{-1}.

HRMS (EI)$^+$: *m/z* for $C_{18}H_{16}O_2$ $[M]^+$: calc.: 264.1145, found: 264.1143.

(Z)-3,3'-(But-1-en-3-yne-1,4-diyl)dipyridine (3p)

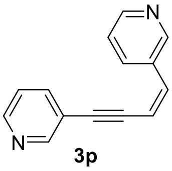

3p

According to General Procedure 3 the title compound was prepared using 3-ethynylpyridine (103 mg, 1.00 mmol, 1.00 equiv.), $[CoBr_2(TriPhos)]$ (75.3 mg, 100 µmol, 0.10 equiv.), zinc dust (13.1 mg, 200 µmol, 0.20 equiv.) and zinc iodide (147 mg, 460 µmol, 0.46 equiv.) in acetonitrile (0.67 mL). The alkyne was added at 0 °C, the mixture was warmed to room temperature and stirred for 28 h. The product was obtained after column chromatography (eluent: ethyl acetate) as a light-brown oil (18.8 mg, 91.2 µmol, 18%, *E:Z* = 10:90).

^{1}H NMR (300 MHz, $CDCl_3$) δ = 8.95 (d, *J* = 2.3 Hz, 1H), 8.72 (d, *J* = 2.1 Hz, 1H), 8.59 – 8.51 (m, 2H), 8.34 (dt, *J* = 8.1, 1.9 Hz, 1H), 7.80 – 7.70 (m, 1H), 7.31 (td, *J* = 7.7, 3.9 Hz, 2H), 6.74 (d, *J* = 11.9 Hz, 1H), 6.06 (d, *J* = 11.9 Hz, 1H) ppm.

^{13}C NMR (75 MHz, $CDCl_3$) δ = 152.2, 150.5, 149.6, 149.1, 138.5, 136.1, 135.1, 132.2, 123.4, 123.3, 120.2, 109.5, 93.4, 90.6 ppm.

IR (ATR): $\tilde{\nu}$ = 3023, 2963, 1586, 1563, 1479, 1426, 1407, 1216, 1024, 907, 804, 753, 731, 704, 669, 650, 626 cm$^{-□}$.

HRMS (EI)$^+$: *m/z* for $C_{14}H_{10}N_2$ $[M]^+$: calc.: 206.0838, found: 206.0832.

(Z)-2,2'-(But-1-en-3-yne-1,4-diyl)dithiophene (3r)

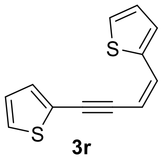

3r

According to General Procedure 3 the title compound was prepared using 3-ethynylthiophene (108 mg, 1.00 mmol, 1.00 equiv.), $[CoBr_2(TriPhos)]$ (75.3 mg, 100 µmol, 0.10 equiv.), zinc dust (13.1 mg, 200 µmol, 0.20 equiv.) and zinc iodide (147 mg, 460 µmol, 0.46 equiv.) in acetonitrile (0.67 mL). The alkyne was added at 0 °C, the mixture was warmed to room temperature and stirred for 4 h. The product was obtained after column chromatography (eluent: *n*-pentane:CH_2Cl_2 = 10:1) as a yellow solid (47.5 mg, 220 µmol, 44%, *E:Z* = 3:97).

melting point: 52 – 55 °C.

^{1}H NMR (500 MHz, $CDCl_3$) δ = 7.37 (dt, *J* = 5.1, 1.0 Hz, 1H), 7.33 (td, *J* = 4.9, 1.1 Hz, 2H), 7.30 – 7.28 (m, 1H), 7.05 (ddd, *J* = 5.2, 3.6, 2.1 Hz, 2H), 6.96 (dt, *J* = 11.5, 0.7 Hz, 1H), 5.77 (d, *J* = 11.3 Hz, 1H) ppm.

^{13}C NMR (126 MHz, $CDCl_3$) δ = 141.0, 132.4, 131.9, 129.8, 127.9, 127.4, 127.3, 126.6, 123.7, 104.4, 92.6, 92.3 ppm.

IR (ATR): $\tilde{\nu}$ = 3100, 3076, 2182, 1900, 1794, 1730, 166, 1660, 1582, 1537, 1516, 1499, 1433, 1419, 1393, 1350, 1334, 1267, 1212, 1197, 1116, 1077, 1043, 980, 941, 899, 847, 829, 764, 746, 727, 707, 693, 590, 569, 544, 526, 504 cm^{-1}.

HRMS (EI)$^+$: *m/z* for $C_{12}H_8S_2$ $[M]^+$: calc.: 216.0062, found: 216.0054.

Reaction Optimization for the Synthesis of *E*-1,3-Enynes

Determination of categorical parameters

Table S1. Optimization of the cobalt source for the *E*-selective dimerization of terminal alkynes.

Entry	Cobalt salt	Zinc iodide	Conversion	Yield[a]
**1**	**$CoBr_2$**	**no**	**100%**	**52%**
2	$CoBr_2$	yes	100%	49%
3	$Co(OAc)_2$	no	100%	50%
4	$CoCl_2$	no	0%	0%
5	$Co(ClO_4)_2 \cdot 6H_2O$	no	0%	0%
6	$Co(BF_4)_2 \cdot 6H_2O$	no	0%	0%

[a] All reactions were carried out on a 0.5 mmol scale using phenylacetylene as test substrate in 0.5 mL MeCN. For all reactions 5 mol% cobalt salt, 5 mol% dppp as ligand, 10 mol% zinc dust and 0.55 equiv. triethylamine were used. The yields were determined via GC/FID using mesitylene (1.0 M in CH_2Cl_2, 0.5 mL, 0.5 mmol, 1.00 equiv.) as internal standard. The internal standard was added after 16 h reaction time.

Table S2. Optimization of the solvent for the *E*-selective dimerization of terminal alkynes.

Entry	Solvent	Conversion	Yield[a]
1	THF	100%	40%
**2**	**MeCN**	**100%**	**52%**
3	DMF	100%	50%
4	DMA	100%	51%
5	CH_2Cl_2	10%	3%

[a] All reactions were carried out on a 0.5 mmol scale using phenylacetylene as test substrate in 0.5 mL corresponding solvent. For all reactions 5 mol% cobalt salt, 5 mol% ligand, 10 mol% zinc dust and 0.55 equiv. triethylamine were used. The yields were determined via GC/FID using mesitylene (1.0 M in CH_2Cl_2, 0.5 mL, 0.5 mmol, 1.00 equiv.).) as internal standard. The internal standard was added after 16 h reaction time. DMA: Dimethylacetamide

Table S3. Optimization of the bidentate ligand for the *E*-selective Dimerization of terminal alkynes.

Entry	Ligand	Conversion	Yield[a]
1	dppm	0%	0%
2	dppe	44%	22%
**3**	**dppp**	**100%**	**52%**
4	dppb	0%	0%
5	dppf	0%	0%
6	PyImine	100%	0%[b]
7	$(IPrIm)_2Py$	100%	0%[b]

[a] All reactions were carried out on a 0.5 mmol scale using phenylacetylene as test substrate in 0.5 mL MeCN. For all reactions 5 mol% cobalt salt, 5 mol% ligand, 10 mol% zinc dust and 0.55 equiv. triethylamine were used. The yields were determined via GC/FID using mesitylene (1.0 M in CH_2Cl_2, 0.5 mL, 0.5 mmol, 1.00 equiv.) as internal standard. The internal standard was added after 16 h reaction time. dppm: Bis(diphenylphosphino)methane; dppe: 1,2-Bis(diphenylphosphino)ethane; dppp: 1,3-Bis(diphenylphosphino)propane; dppb: 1,4-Bis(diphenylphosphino)butane; dppf: 1,1'-Bis(diphenylphosphino)ferrocene, PyImine: *N*-mesityl-1-(pyridin-2-yl)methanimine; $(IPrIm)_2Py$: 1,1'-(pyridine-2,6-diyl)bis(N-(2,6-diisopropylphenyl)ethan-1-imine). [b] Only cyclotrimerization product could be observed.

Table S4. Optimization of the additive for the *E*-selective dimerization of terminal alkynes.

Entry	Additive	Conversion	Yield[a]
1	NEt_3	100%	52%
2	Pyrrolidine	100%	45%
3	Pyridine	7%	2%
4	K_2CO_3	100%	43%
5	KOPiv	0%	0%
6	TMEDA	85%	38%
7	Glycine	78%	34%
**6**	**PPh_3**	**100%**	**70%[b]**

[a] All reactions were carried out on a 0.5 mmol scale using phenylacetylene as test substrate in 0.5 mL MeCN. For all reactions 5 mol% $CoBr_2$(dppp), 10 mol% zinc dust and 0.55 equiv. additive were used. The yields were determined via GC/FID using (1.0 M in CH_2Cl_2, 0.5 mL, 0.5 mmol, 1.00 equiv.) as internal standard. The internal standard was added after 16 h reaction time. [b] 4-Fluorophenylacetylene was used. The reaction mixture was quenched after 1 h reaction time.

Reaction Optimization for the *E*-selective hydroalkynylation of terminal alkynes using *Design of Experiments*

A D-optimal screening design was generated by using *JMP 13* software package by SAS (version 13.2.1, SAS Institute Inc, Cary, NC, © 2016). The generated design considered all linear and quadratic terms of the numerical values. After running all initial experiments, the screening design was extended to consider possible cross interactions (temperature · substrate concentration, temperature · time, substrate concentration · time, catalyst loading · time, catalyst loading · zinc iodide). In this case only one cross interaction had a low p-value (temperature · substrate concentration). All other tested cross interactions did not have a big significance on the screening design. In total the design consisted of 25 reactions, excluding one verified outlier. For the *lack of fit* value three experiments were duplicated. At last three additional experiments were conducted for to gain a higher significance of the quadratic term of the triphenyl phosphine equivalents.

General Procedure 4:

Under Argon atmosphere all solids were added into a pre-dried reaction vessel. The solids were dried *in vacuo* for 15 min. Afterwards the catalyst system was dissolved in acetonitrile (0.33 - 2.50 mL) using standard single use syringes. The mixture was shortly heated up to approximately 80 °C. whereas a color-change from dark blue/green to dark green appeared. The resulting solution was cooled to the desired temperature and the test substrate (4-fluorophenylacetylene) was added (60.0 µL, 500 µmol, 1.00 equiv., via Eppendorf™ pipette) to the catalyst system. After the desired reaction time, mesitylene (1.0 M in CH_2Cl_2, 0.5 mL, 500 µmol, 1.00 equiv., via syringe) and hexafluorobenzene (57.7 µL, 500 µmol, 1.00 equiv., via Eppendorf™ pipette) were added. The yield of the product was determined via GC/FID and ^{19}F NMR spectroscopy.

Table S5. Optimization reactions of *DoE* for the *E*-selective hydroalkynylation of terminal alkynes. All reactions were carried out on a 0.5 mmol scale, according to General Procedure 4. The predicted optimal reaction conditions were verified. Predicted yield: 85%, isolated yield: 82%.

Entry	catalyst loading [mol%]	Equiv. PPh_3	Temperature [°C]	Time [h]	zinc iodide	substrate concentration [M]	Yield [%]
1	6	1	55	6	No	1.50	3
2	2	3	27.5	0.5	No	1.43	62
3	6	2	55	6	Yes	0.20	3
4	2	3	55	6	Yes	0.20	33
5	4	3	55	3.25	No	0.85	37
6	2	1	27.5	6	Yes	0.85	40
7	6	1	27.5	3.25	Yes	1.43	20
8	6	1	0	6	Yes	0.85	47
9	4	1	0	0.5	No	1.43	75
10	2	1	55	0.5	No	0.20	43
11	2	2	55	0.5	Yes	1.43	29
12	2	2	0	3.25	No	0.20	60
13	6	1	55	0.5	No	0.20	24
14	6	3	0	0.5	Yes	0.20	21
15	2	1	55	6	No	1.43	19
16	4	1	0	6	No	0.20	52
17	6	2	0	0.5	No	0.85	71
18	6	3	0	6	No	1.50	71
19	6	3	27.5	6	No	0.20	46
20	6	3	55	0.5	Yes	1.43	8
21	2	3	0	6	No	1.43	76
22	4	2	27.5	6	Yes	1.43	35
23	6	2	27.5	2	No	0.25	48
24	3	2	0	1	No	0.50	71
25	2	1	15	3.25	Yes	1.43	52
**Replication**	2	3	27.5	0.5	No	1,43	56
**Replication**	4	1	0	0.5	No	1,43	73
**Replication**	6	2	0	0.5	No	0,85	72
PPh_3-1	2	2	0	2	No	1.25	85
PPh_3-2	2	2,5	0	2	No	1.25	77
PPh_3-3	2	3	0	2	No	1.25	78

Response yield

Actual by Predicted Plot

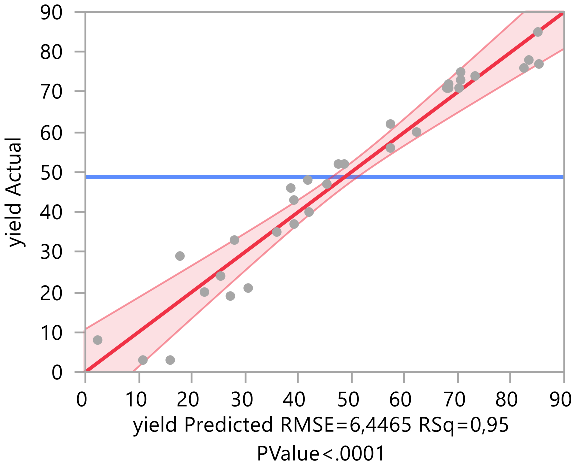

Effect Summary

Source	LogWorth	PValue
temperature (0,55)	10,239	0,00000
zinc iodide	5,597	0,00000
temperature*substrate concentration	4,635	0,00002
catalyst loading (2,6)	4,535	0,00003
substrate concentration*substrate concentration	1,873	0,01338
PPh_3 equivalents*PPh_3 equivalents	1,695	0,02019
substrate concentration (0,2,1,5)	0,542	0,28719 ^
PPh_3 equivalents (1,4)	0,430	0,37123 ^

Lack of Fit

Source	DF	Sum of Squares	Mean Square	F Ratio
Lack of Fit	20	935,32117	46,7661	6,8438
Pure Error	3	20,50000	6,8333	**Prob > F**
Total Error	23	955,82117		0,0690
				**Max RSq**
				0,9989

Residual by Predicted Plot

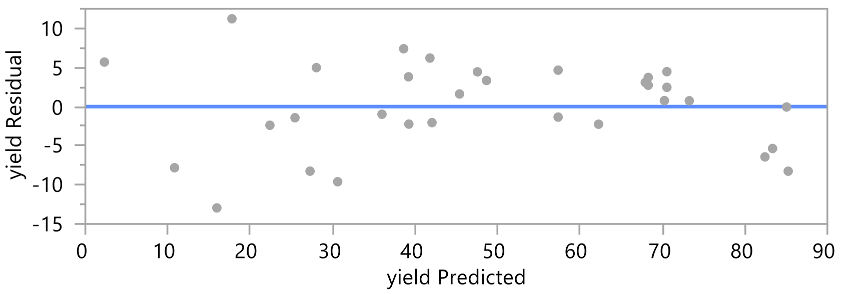

Studentized Residuals

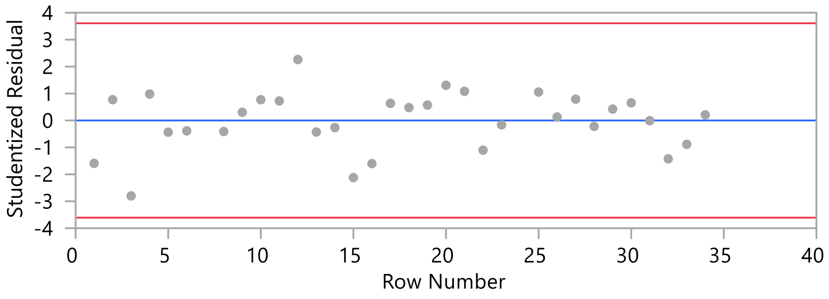

Externally Studentized Residuals with 95% Simultaneous Limits (Bonferroni)

Box-Cox Transformations

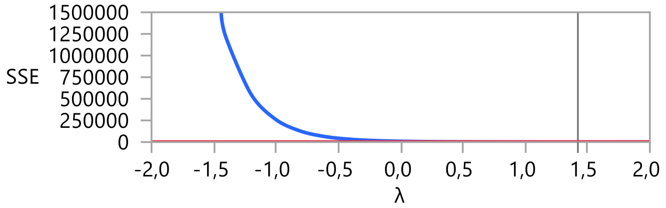

Best λ=1,416

Parameter Estimates

Term	Estimate	Std Error	t Ratio	Prob>|t|	VIF
Intercept	50,156483	2,905661	17,26	<,0001*	.
catalyst loading (2,6)	-6,884957	1,326701	-5,19	<,0001*	1,1035278
PPh_3 equivalents (1,4)	-2,546204	2,791892	-0,91	0,3712	2,0990186
temperature (0,55)	-17,11118	1,496527	-11,43	<,0001*	1,2343686
zinc iodide [no]	8,0900787	1,305293	6,20	<,0001*	1,1274561
substrate concentration (0,2,1,5)	1,6308368	1,496768	1,09	0,2872	1,1154021
PPh_3 equivalents*PPh_3 equivalents	-9,452752	3,787714	-2,50	0,0202*	2,0754947
substrate concentration*substrate concentration	-9,225459	3,442815	-2,68	0,0134*	1,1482148
temperature*substrate concentration	-8,934884	1,691195	-5,28	<,0001*	1,0599042

Prediction Profiler

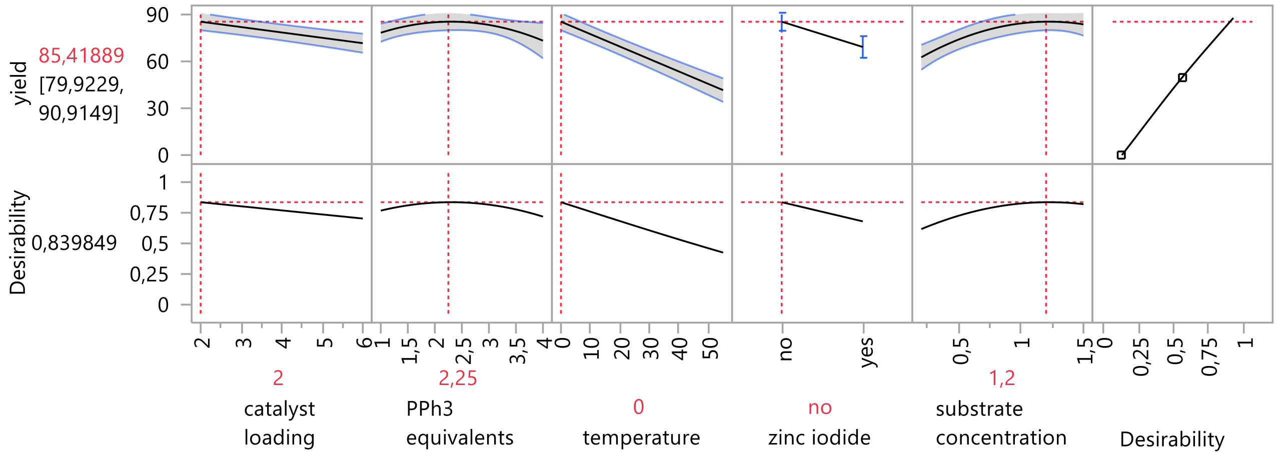

The results of the screening design were verified by *k*-fold cross validation (*k* = 5).

The higher p-value of the linear terms (substrate concentration and triphenylphosphine equivalents) could be a result of inaccuracies of the scale weighing small amounts of triphenylphosphine and inexact voluminal of the solvent using single-use syringes.

Reaction Optimization for the Synthesis of *Z*-1,3-Enynes

Determination of categorical parameters

Table S6. Optimization of the solvent for the *Z*-selective dimerization of terminal alkynes.

Entry	Solvent	Yield[a]
1	THF	26%
**2**	**MeCN**	**41%**
3	EtOAc	12%
4	Acetone	19%
5	DMF	22%
5	CH_2Cl_2	3%

[a] All reactions were carried out on a 0.5 mmol scale using phenylacetylene as test substrate in 0.5 mL corresponding solvent. For all reactions 5 mol% [$CoBr_2$(TriPhos)], 10 mol% zinc dust and 1.00 equiv. Triethylamine were used. The yields were determined via GC/FID using (1.0 M in CH_2Cl_2, 0.5 mL, 0.5 mmol, 1.00 equiv.) as internal standard. The internal standard was added after 16 h reaction time.

Table S7: Optimization of the Lewis acid for the *Z*-selective dimerization of terminal alkynes.

Entry	Lewis Acid	Conversion	Yield[a]
**1**	**ZnI_2[b]**	**95%**	**52%**
2	$ZnBr_2$	87%	44%
3	$ZnCl_2$	84%	36%
4	$AlCl_3$	54%	14%
5	$BF_3{\cdot}OEt_2$	18%	0%
6	B(O*i*Pr)$_3$	69%	26%

[a] All reactions were carried out on a 0.5 mmol scale using (4-fluorophenyl)acetylene as test substrate in 0.5 mL acetonitrile. For all reactions 5 mol% [$CoBr_2$(TriPhos)], 10 mol% zinc dust and 10 mol% Lewis acid were used. The yields were determined via GC/FID using (1.0 M in CH_2Cl_2, 0.5 mL, 0.5 mmol, 1.00 equiv.) as internal standard. The internal standard was added after 16 h reaction time. [b] ZnI_2 (50 mol%) were used.

Table S8: Optimization of the counter ion and additives for the *Z*-selective dimerization of terminal alkynes.

Entry	Cobalt salt	Zinc iodide	Additive	Conversion	Yield[a]	*E*/*Z ratio*
1	$CoBr_2$	Yes	PPh_3 (5 mol%)	76%	41%	5:95
2	$CoBr_2$	Yes	PPh_3 (4 mol%)	76%	42%	5:95
3	$CoBr_2$	Yes	PPh_3 (10 mol%)	71%	26%	50:50
**4**	**$CoBr_2$**	**Yes**	**None**	**80%**	**44%**	**5:95**
5	$CoBr_2$	No	PPh_3 (5 mol%)	87%	9%	12:88
6	$Co(OAc)_2$	No	None	40%	16%	12:88
7	$CoCl_2$	No	None	76%	30%%	5:95
8	$CoCl_2$	Yes	None	65%	23%	17:83
9	$Co(ClO_4)_2{\cdot}6H_2O$	No	NEt_3	60%	23%	6:94
10	$Co(BF_4)_2{\cdot}6H_2O$	No	None	54%	7%	29:71

Entry	Cobalt salt	Zinc iodide	Additive	Conversion	Yield[a]	*E*/*Z ratio*
11	$Co(BF_4)_2{\cdot}6H_2O$	No	PPh_3 (5 mol%)	71%	14%	29:71
12	$CoBr_2$	Yes	Pyridine (10 mol%)	89%	40%	7:93
13	$CoBr_2$	Yes	2,6-Lutidine (10 mol%)	90%	28%	7:93
14	$CoBr_2$	Yes	4-Chlorostyrene (10 mol%)	86%	37%	5:95
15	$CoBr_2$	Yes	Pyridine (10 mol%)	89%	40%	7:93
16	$CoBr_2$	Yes	Pcy_3 (5 mol%)	81%	36%	3:97
17	$CoBr_2$	Yes	*t*Bu_3P (5 mol%)	62%	10%	10:90
18	$CoBr_2$	Yes	$(2,6\text{-}MeOC_6H_3)_3P$ (5 mol%)	72%	29%	3:97

[a] All reactions were carried out on a 0.5 mmol scale using (4-fluorophenyl)acetylene as test substrate in 0.5 mL acetonitrile. For all reactions 5 mol% [$CoBr_2$(TriPhos)], 10 mol% zinc dust, 10 mol% zinc iodide and the corresponding additive were used. The yields were determined via GC/FID using (1.0 M in CH_2Cl_2, 0.5 mL, 0.5 mmol, 1.00 equiv.) as internal standard. The internal standard was added after 16 h reaction time. PPh_3: Triphenylphosphine, Pcy_3: Tricyclohexylphosphine, *t*Bu_3P: Tri-*tert*-butylphosphine, $(2,6\text{-}MeOC_6H_3)_3P$: Tri-(2,6-dimethoxyphenyl)-phosphine.

Table S9: Investigations concerning the oxidation state of the cobalt central atom, the influence of additives and the relevance of each component.

Entry	Reducing agent	ZnI_2	Cobalt salt	Yield[a]
1	---	---	$CoBr_2$	10%
2	---	Yes	$CoBr_2$	5%
3	---	Yes	---	0%
4	Zn	Yes	---	0%
5	Zn	Yes	$CoBr_2$	50%
6	---	Yes	$CoBr_2$	0%[b]
6	Mg	No	$CoBr_2$	0%
7	EtMgBr	No	$CoBr_2$	20%

[a] All reactions were carried out on a 0.5 mmol scale using (4-fluorophenyl)acetylene as test substrate in 0.5 mL acetonitrile. The yields were determined via GC/FID using (1.0 M in CH_2Cl_2, 0.5 mL, 0.5 mmol, 1.00 equiv.) as internal standard. The internal standard was added after 16 h reaction. [b] The reaction was performed under O_2-atomsphere.

Reaction Optimization for the *Z*-selective hydroalkynylation of terminal alkynes using *Design of Experiments*

A D-optimal screening design was generated by using *JMP 13* software package by SAS (version 13.2.1, SAS Institute Inc, Cary, NC, © 2016). The generated design considered all linear and quadratic terms of the numerical parameters. After running all initial experiments, the screening design was extended to consider possible cross interactions and to expand the zinc iodide equivalents from 4 – 60 mol to 4 – 100 mol%. In this case no cross interaction had a low p-value. In total the design consisted of 30 reactions, excluding two verified outliers. For the *lack of fit* value six experiments were replicated. At last three experiments were run to verify the predicted optimum and reaction time.

General Procedure 5:

Under Argon atmosphere all solids were added into a pre-dried reaction vessel. The solids were dried *in vacuo* for 15 min. Afterwards the catalyst system was dissolved in acetonitrile (0.33 - 2.50 mL) using standard single use syringes. The mixture was stirred 15 min at 37 °C. The suspension was cooled or heated to the desired temperature and the test substrate (4-fluorophenylacetylene) was added via Eppendorf™ pipette (60.0 µL, 500 µmol, 1.00 equiv.) to the catalyst system. After the corresponding reaction time mesitylene (1.0 M in CH_2Cl_2, 0.5 mL, 500 µmol, 1.00 equiv., via syringe) and hexafluorobenzene (57.7 µL, 500 µmol, 1.00 equiv., via Eppendorf™ pipette) were added. The yield of the product was determined via GC/FID and ^{19}F NMR spectroscopy.

Table S10: Optimization reactions of *DoE* for the *Z*-selective hydroalkynylation of terminal alkynes. All reactions were carried out on a 0.5 mmol scale, according to General Procedure 5. Predicted yield: 67%, isolated yield: 65%.

Entry	catalyst loading [mol%]	Zinc iodide [mol%]	Temperature [°C]	Time [h]	substrate concentration [M]	Yield [%]
1	10	60	25	13.5	0.20	61
2	2	22	60	24	1.50	51
3	10	100	60	13.5	1.50	26
4	2	6.94	60	13.5	0.20	24
5	10	103	-10	24	0.64	0
6	10	26.5	60	3	0.85	59
7	2	12	25	3	1.50	37
8	2	12.7	-10	13.5	0.85	30
9	6	60	25	13.5	0.85	56
10	6	38.4	60	24	0.20	40
11	2	4.88	25	24	0.85	28
12	10	62.3	-10	24	1.50	58
13	6	11.28	-10	13.5	1.50	39
14	6	36	60	3	0.85	45
15	6	12.22	25	3	0.20	28
16	2	100	-10	24	0.20	0
17	10	4	-10	3	1.50	30
18	2	106	25	13.5	1.50	11
19	10	5	25	24	1.50	44
20	2	97	60	3	0.20	11
21	10	100	25	3	1.50	44
22	2	52	-10	24	1.50	35
23	10	4.9	60	24	0.85	40
24	2	4.6	60	3	1.50	23
25	10	52	60	24	0.20	41
26	10	5.7	60	3	0.20	42
27	6	103	60	24	1.50	27

Entry	catalyst loading [mol%]	Zinc iodide [mol%]	Temperature [°C]	Time [h]	substrate concentration [M]	Yield [%]
28	6	4	-10	24	0.20	17
29	10	106	-10	3	0.20	11
30	2	100	-10	3	0.85	3
Replication	10	60	25	13.5	0.20	61
Replication	2	22	60	24	1.50	50
Replication	10	100	60	13.5	1.50	38
Replication	10	100	-10	24	0.64	0
Replication	6	60	25	13.5	0.85	57
Replication	10	52	60	24	0.20	46
Optimum-1	10	46	37	6	1.50	67
Optimum-2	10	46	37	3.5	1.50	65
Optimum-3	10	46	37	4	1.50	62

Response yield

Actual by Predicted Plot

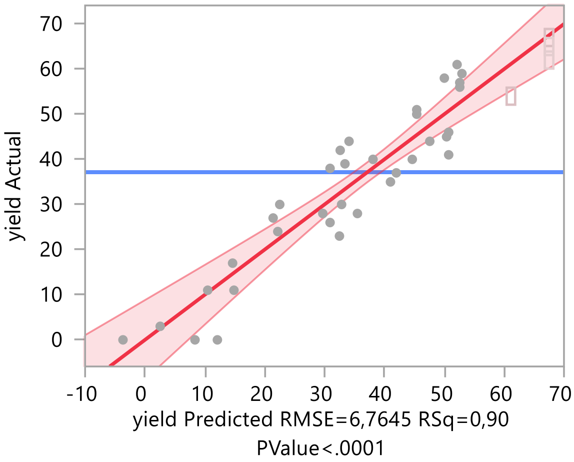

Effect Summary

Source	LogWorth	PValue
zinc iodide mol%*zinc iodide mol%	10,514	0,00000
substrate concentration(0,2,1,5)	4,382	0,00004
catalyst loading(2,10)	3,990	0,00010
zinc iodide mol%(4,100)	3,752	0,00018 ^
temperature (-10,60)	3,190	0,00065
temperature*temperature	2,801	0,00158

Lack of Fit

Source	DF	Sum of Squares	Mean Square	F Ratio
Lack Of Fit	26	1366,1139	52,5428	3,2114
Pure Error	6	98,1667	16,3611	**Prob > F**
Total Error	32	1464,2806		0,0748
				**Max RSq**
				0,9930

Residual by Predicted Plot

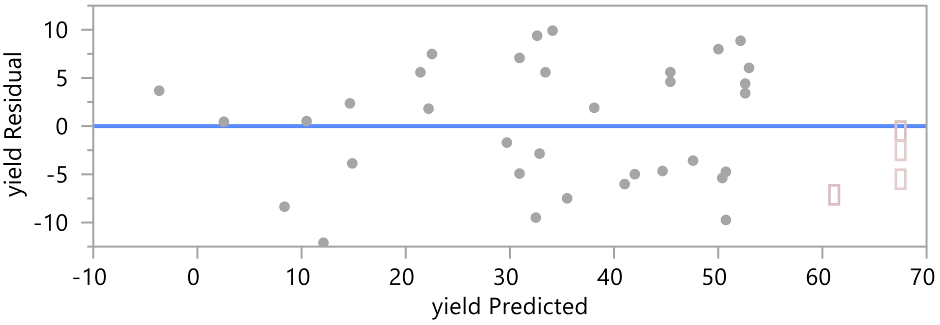

Studentized Residuals

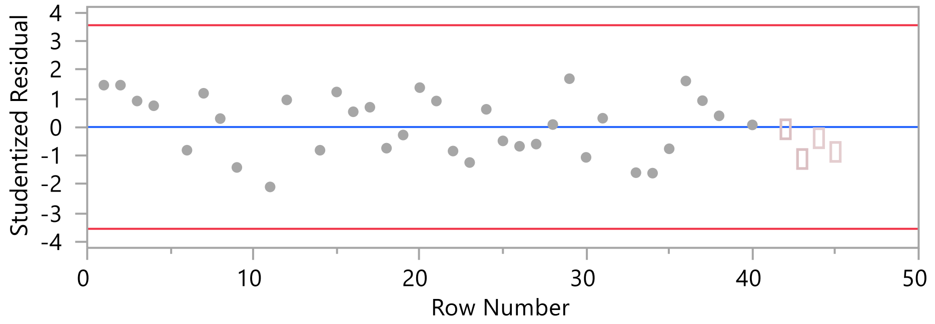

Externally Studentized Residuals with 95% Simultaneous Limits (Bonferroni)

Parameter Estimates

Term	Estimate	Std Error	t Ratio	Prob>|t|	VIF
Intercept	54,354785	2,292617	23,71	<,0001*	.
catalyst loading(2,10)	5,7715943	1,301911	4,43	0,0001*	1,1017458
zinc iodide mol%(4,100)	-6,271534	1,47858	-4,24	0,0002*	1,0549539
temperature(-10,60)	5,3083966	1,404013	3,78	0,0006*	1,052395
substrate concentration(0,2,1,5)	6,2179735	1,310094	4,75	<,0001*	1,0305792
zinc iodide mol%*zinc iodide mol%	-25,97876	2,630114	-9,88	<,0001*	1,1923214
temperature*temperature	-8,480181	2,455669	-3,45	0,0016*	1,1097782

Effect Tests

Source	Nparm	DF	Sum of Squares	F Ratio	Prob > F
catalyst loading(2,10)	1	1	899,2979	19,6530	0,0001*
zinc iodide mol%(4,100)	1	1	823,2513	17,9911	0,0002*
temperature(-10,60)	1	1	654,1224	14,2950	0,0006*
substrate concentration(0,2,1,5)	1	1	1030,7835	22,5265	<,0001*
zinc iodide mol%*zinc iodide mol%	1	1	4464,3882	97,5636	<,0001*
temperature*temperature	1	1	545,6884	11,9253	0,0016*

Prediction Profiler

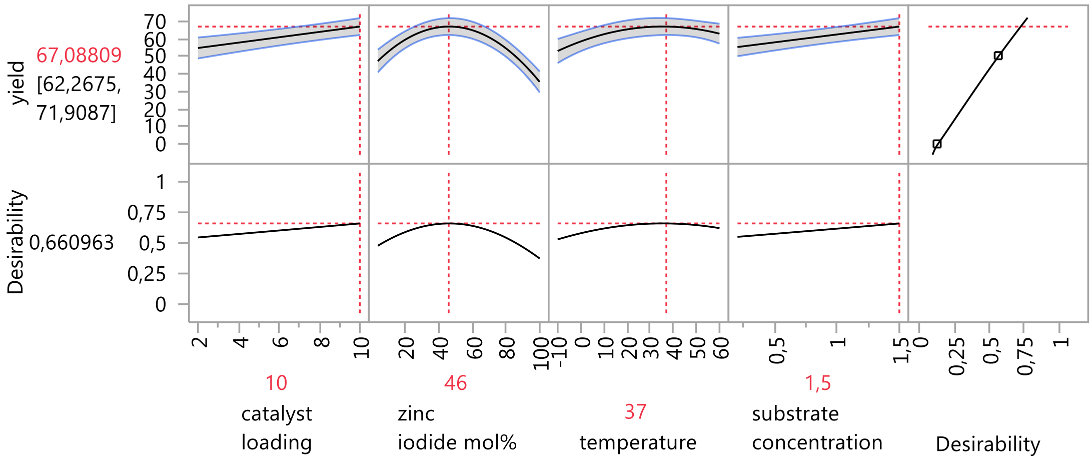

The results of the Screening design were verified by *k*-fold cross validation ($k = 5$).

Literature

[1] G. T. Crisp, B. L. Flynn, *J. Org. Chem.* **1993**, *58*, 6614-6619.

[2] M. I. Pichler, B. Plietker, *Org. Lett.* **2020**, *22*, 340-344.

[3] V. Weingand, T. Wurm, V. Vethacke, M. C. Dietl., D. Ehjeij, M. Rudolph, F. Rominger, J. Xie, A. S. K. Hashmi, *Chem. Eur. J.* **2018**, *24*, 3725-3728.

[4] Y. S. Feng, C. Q. Xie, W. L. Qiao, H. J. Xu, *Org. Lett.* **2013**, *15*, 936-939.

[5] C. Xu, W. Du, Y. Zeng, B. Dai, H. Guo, *Org. Lett.* **2014**, *16*, 948-951.

[6] H. Ueda, M. Yamaguchi, H. Kameya, K. Sugimoto, H. Tokuyama, *Org. Lett*. **2014**, *16*, 4948-4951.

[7] K. Sonogashira, Y. Tohda, N. Hagihara, *Tetrahedron Lett.* **1975**, *50*, 4467-4470.

[8] G. R. Fulmer, A. J. M. Miller, N. H. Sherden, H. E. Gottlieb, A. Nudelman, B. M. Stoltz, J. E. Bercaw, K. I. Goldberg, *Organometallics* **2010**, *29*, 2176-2179.

[9] P. A. W. Dean, D. G. Ibbot, *Can. J. Chem.* **1976**, *54*, 177-187.

[10] G. Hilt, S. Lüers, *Synthesis* **2002**, 609-618.

NMR spectra

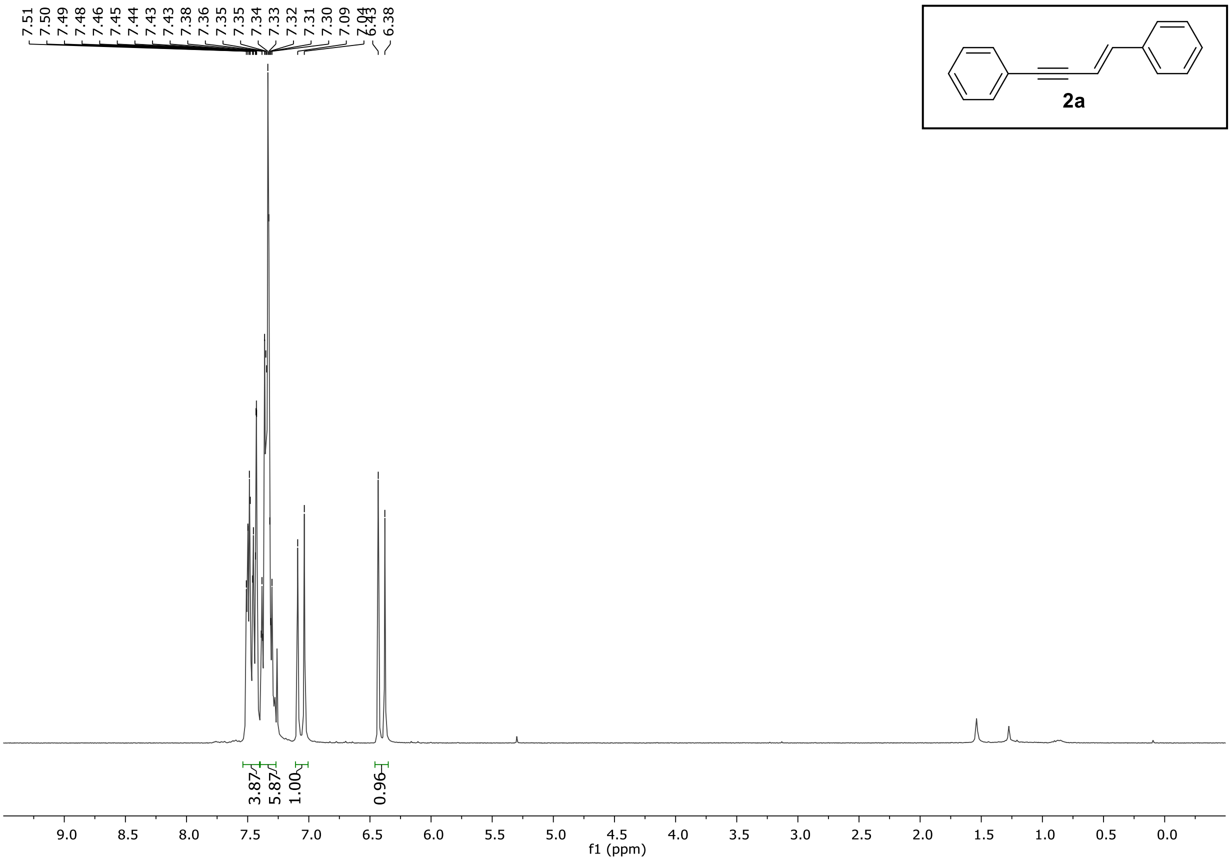
7.51
7.50
7.49
7.48
7.46
7.45
7.44
7.43
7.43
7.38
7.36
7.35
7.35
7.34
7.33
7.33
7.32
7.31
7.30
7.09
7.04
6.43
6.38
3.87
5.87
1.00
0.96
f1 (ppm)
2a

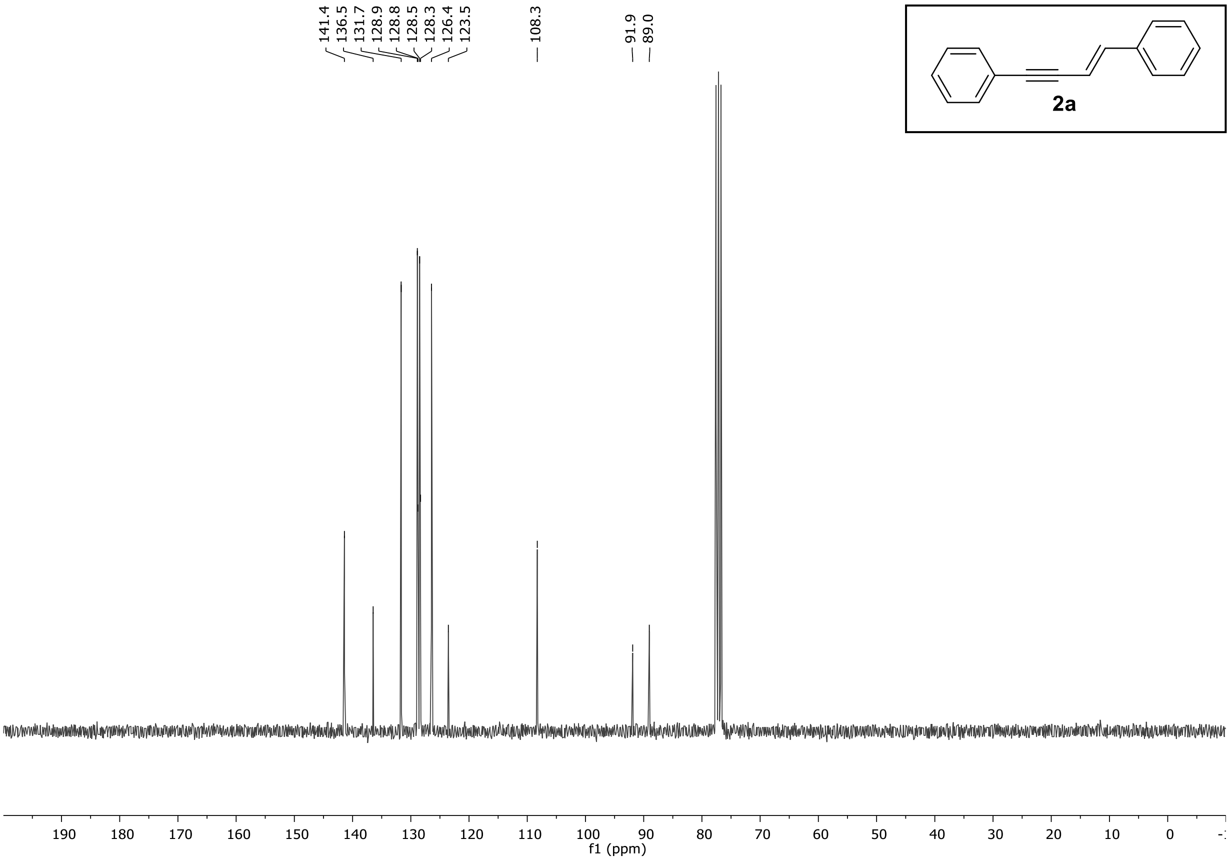
141.4
136.5
131.7
128.9
128.8
128.5
128.3
126.4
123.5
108.3
91.9
89.0
f1 (ppm)
2a

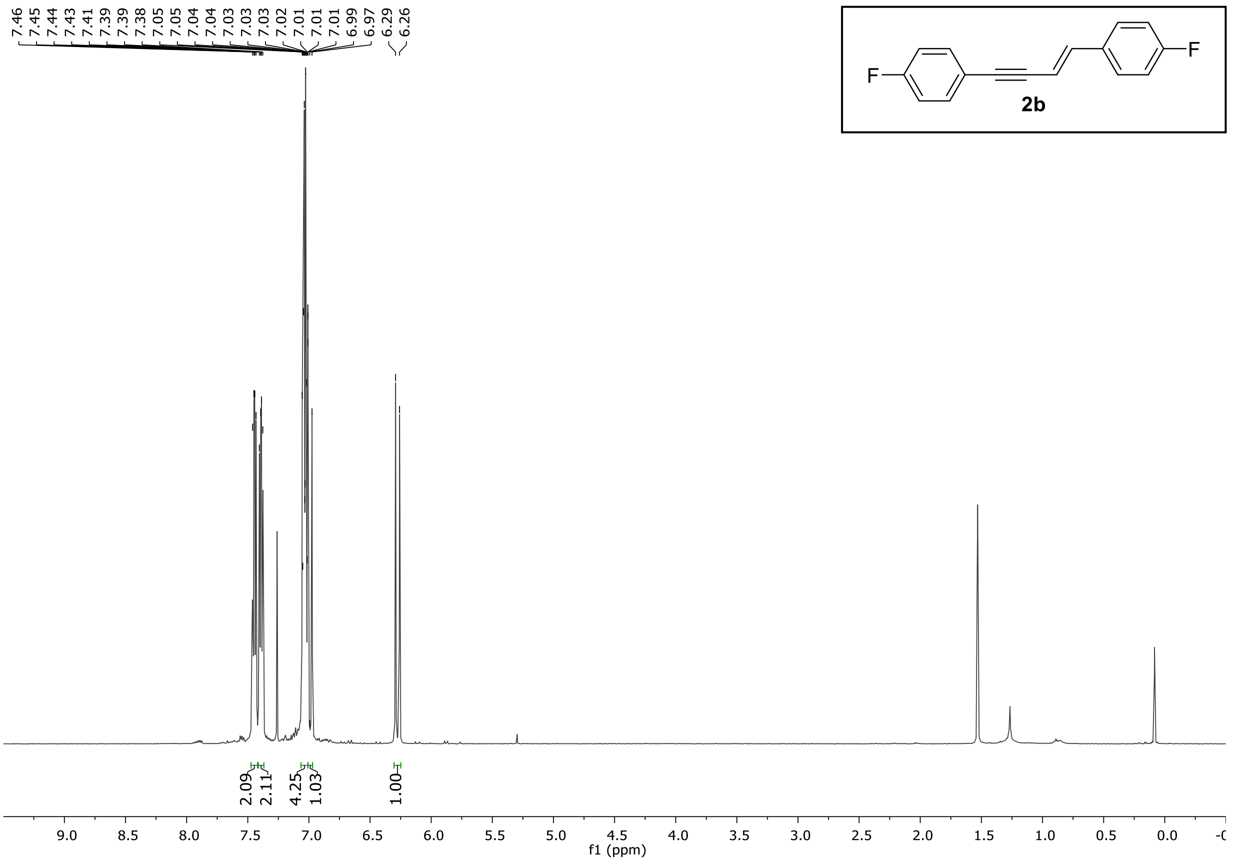
7.46
7.45
7.44
7.43
7.41
7.39
7.39
7.38
7.05
7.05
7.04
7.04
7.03
7.03
7.03
7.02
7.01
7.01
7.01
6.99
6.97
6.29
6.26
2.09
2.11
4.25
1.03
1.00
f1 (ppm)
F
F

2b

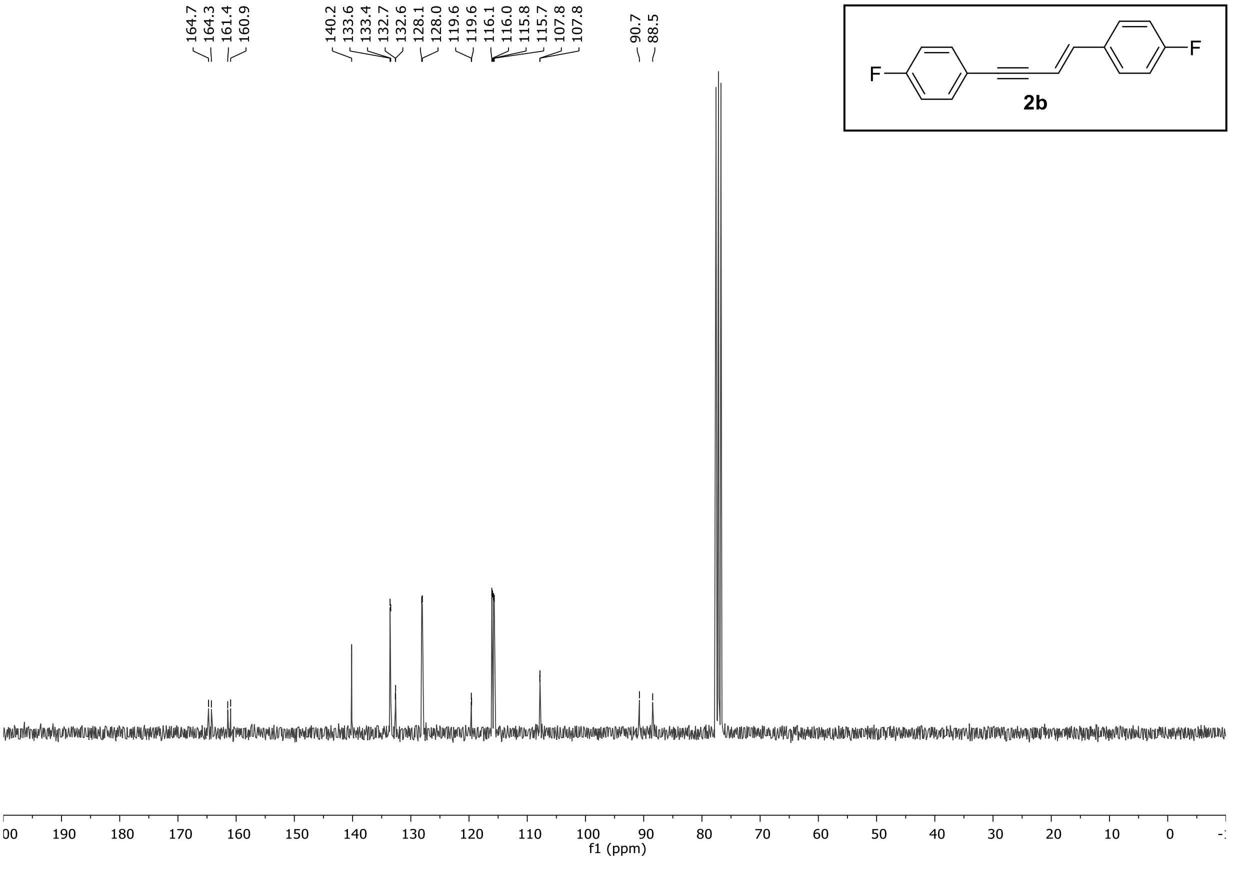
164.7
164.3
161.4
160.9
140.2
133.6
133.4
132.7
132.6
128.1
128.0
119.6
119.6
116.1
116.0
115.8
115.7
107.8
107.8
90.7
88.5
f1 (ppm)
F
F

2b

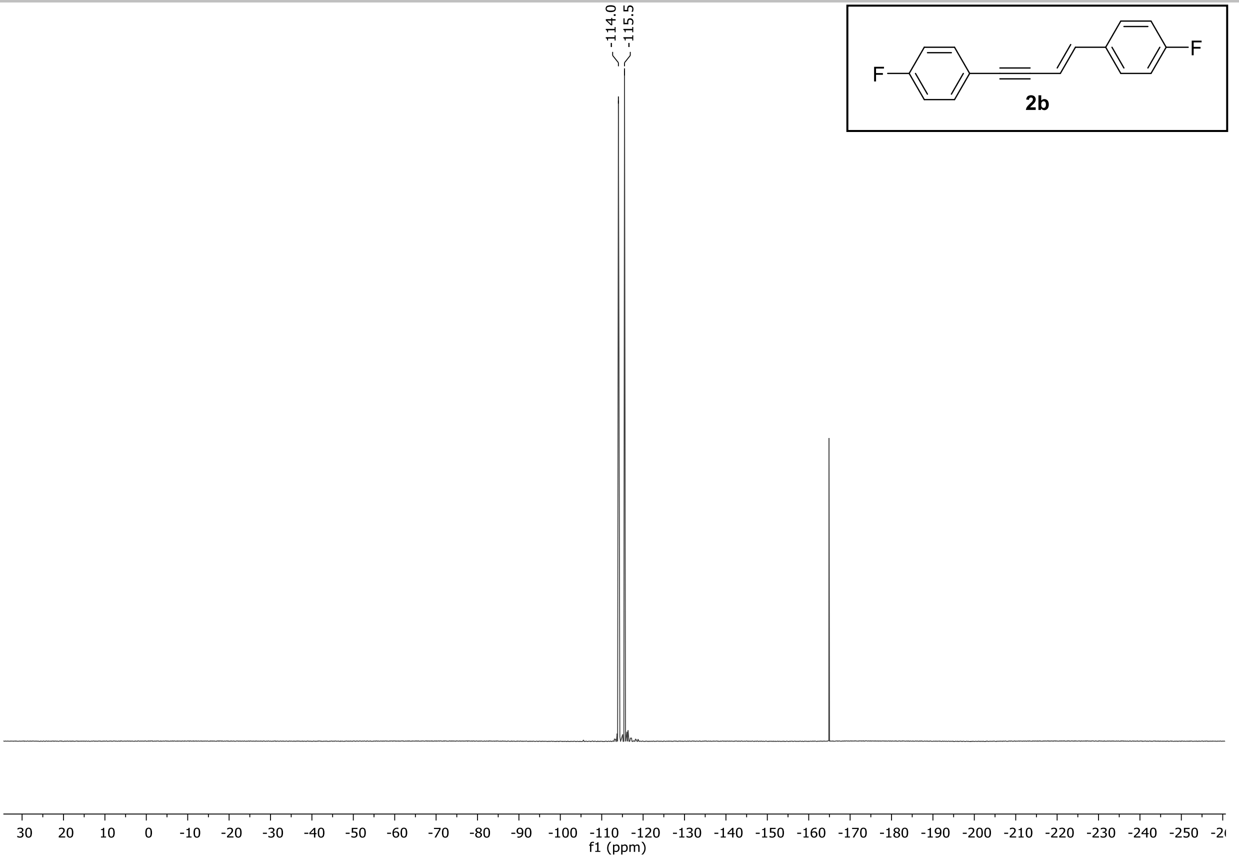
-114.0
-115.5
30
20
10
0
-10
-20
-30
-40
-50
-60
-70
-80
-90
-100
-110
-120
-130
-140
-150
-160
-170
-180
-190
-200
-210
-220
-230
-240
-250
f1 (ppm)
F
F

2b

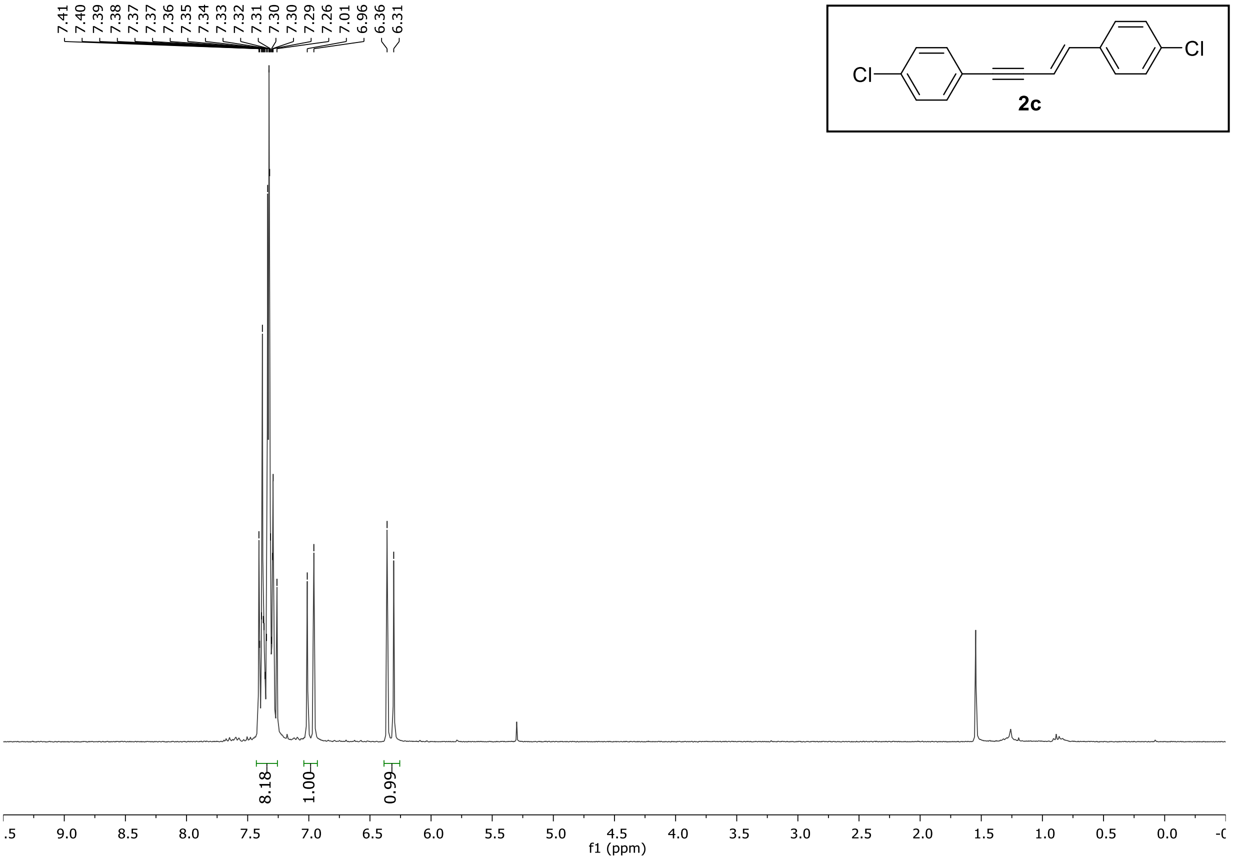
7.41
7.40
7.39
7.38
7.37
7.37
7.36
7.35
7.34
7.33
7.32
7.31
7.30
7.30
7.29
7.26
7.01
6.96
6.36
6.31
8.18
1.00
0.99
f1 (ppm)
Cl
Cl
2c

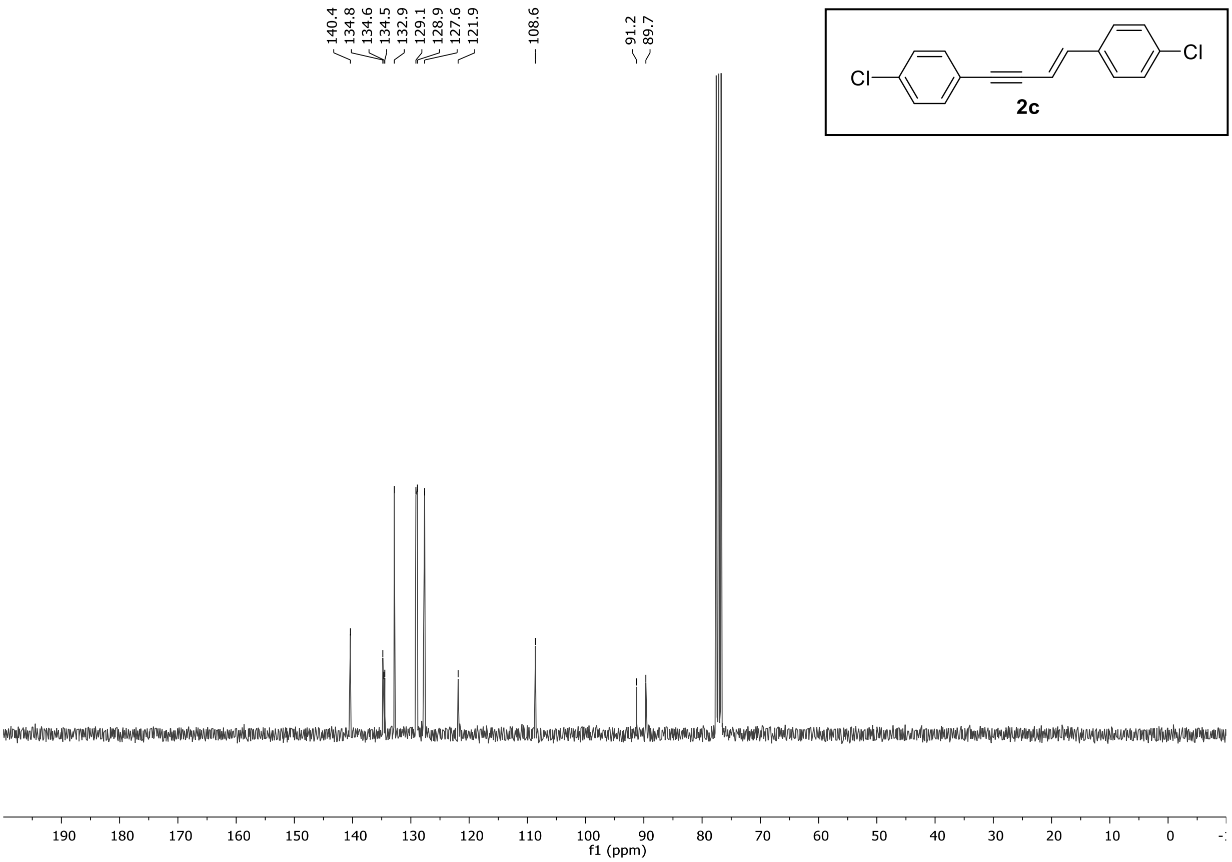
140.4
134.8
134.6
134.5
132.9
129.1
128.9
127.6
121.9
108.6
91.2
89.7
f1 (ppm)
Cl
Cl
2c

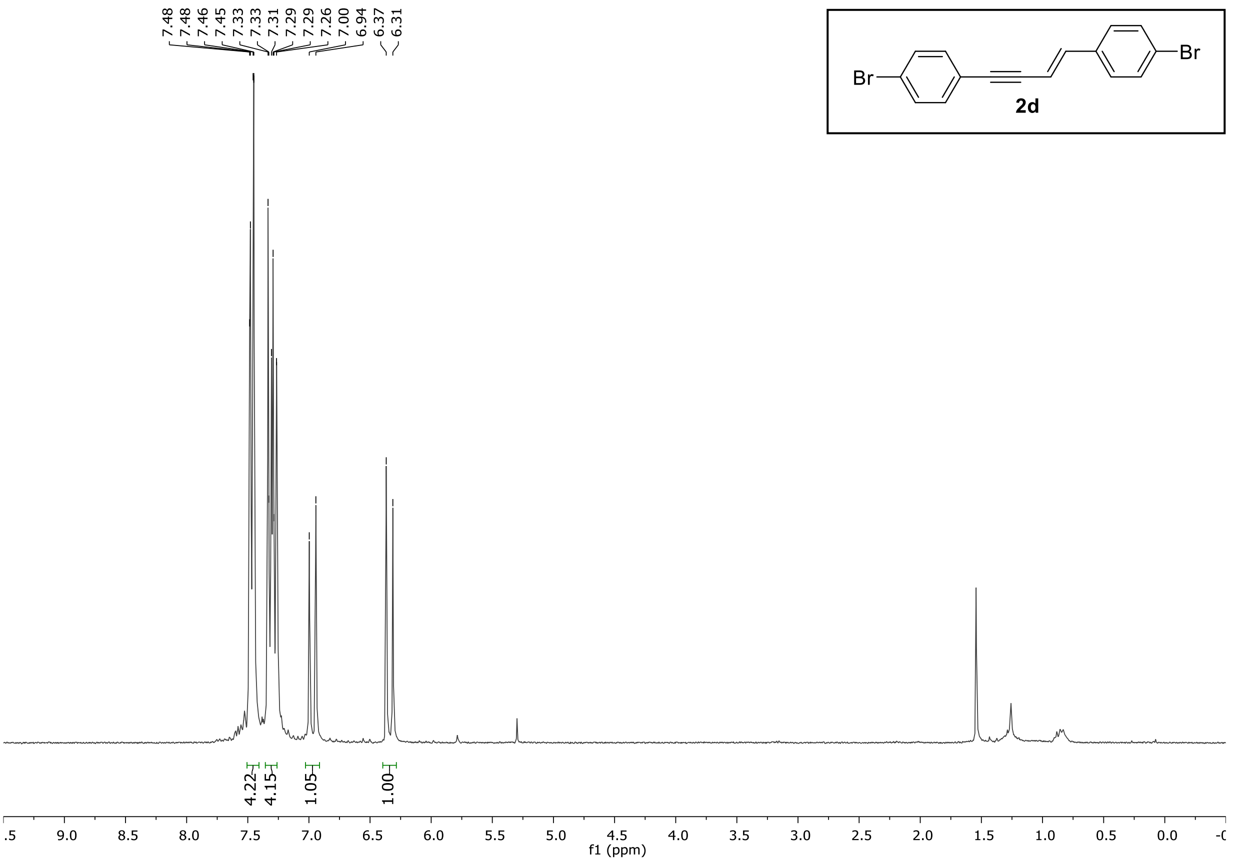
7.48
7.48
7.46
7.45
7.33
7.33
7.31
7.29
7.29
7.26
7.00
6.94
6.37
6.31
Br
Br
2d
4.22
4.15
1.05
1.00
f1 (ppm)

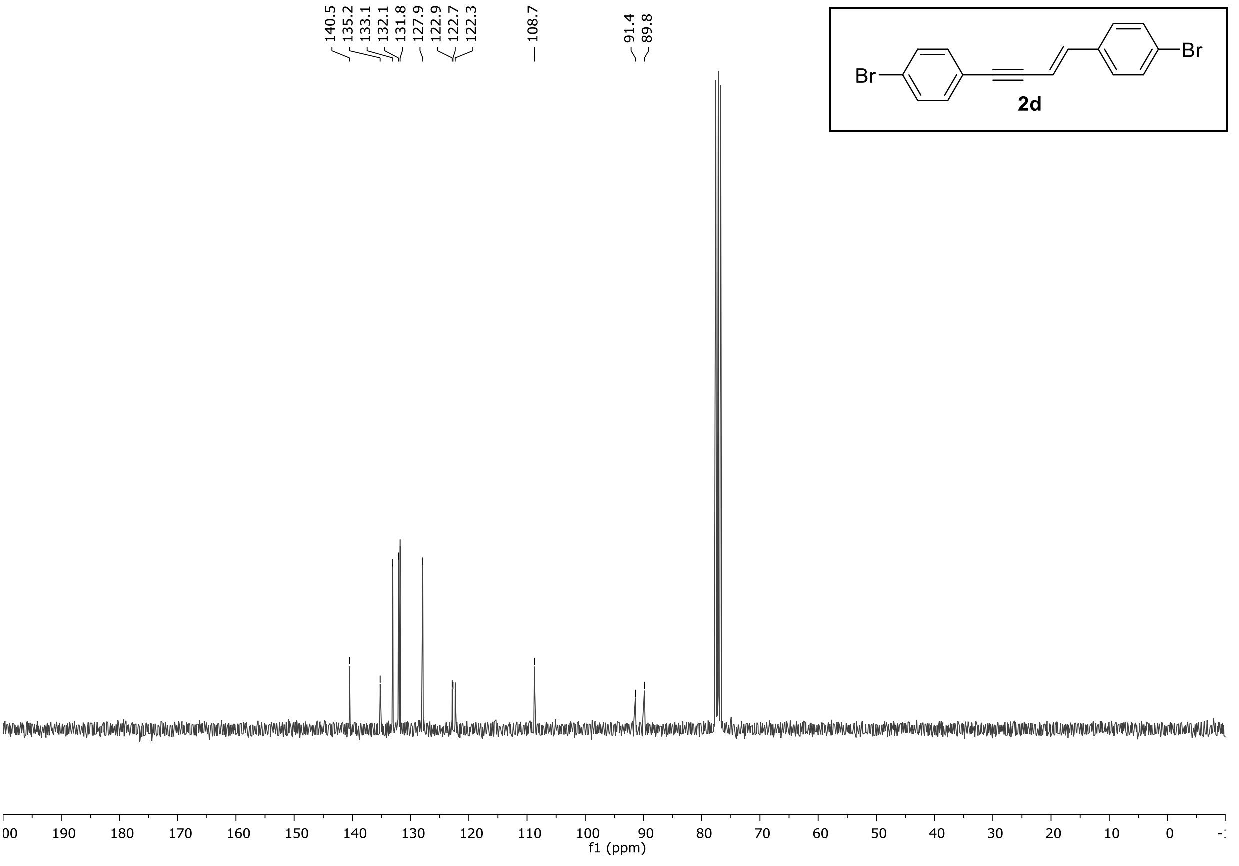
140.5
135.2
133.1
132.1
131.8
127.9
122.9
122.7
122.3
108.7
91.4
89.8
Br
Br
2d
f1 (ppm)

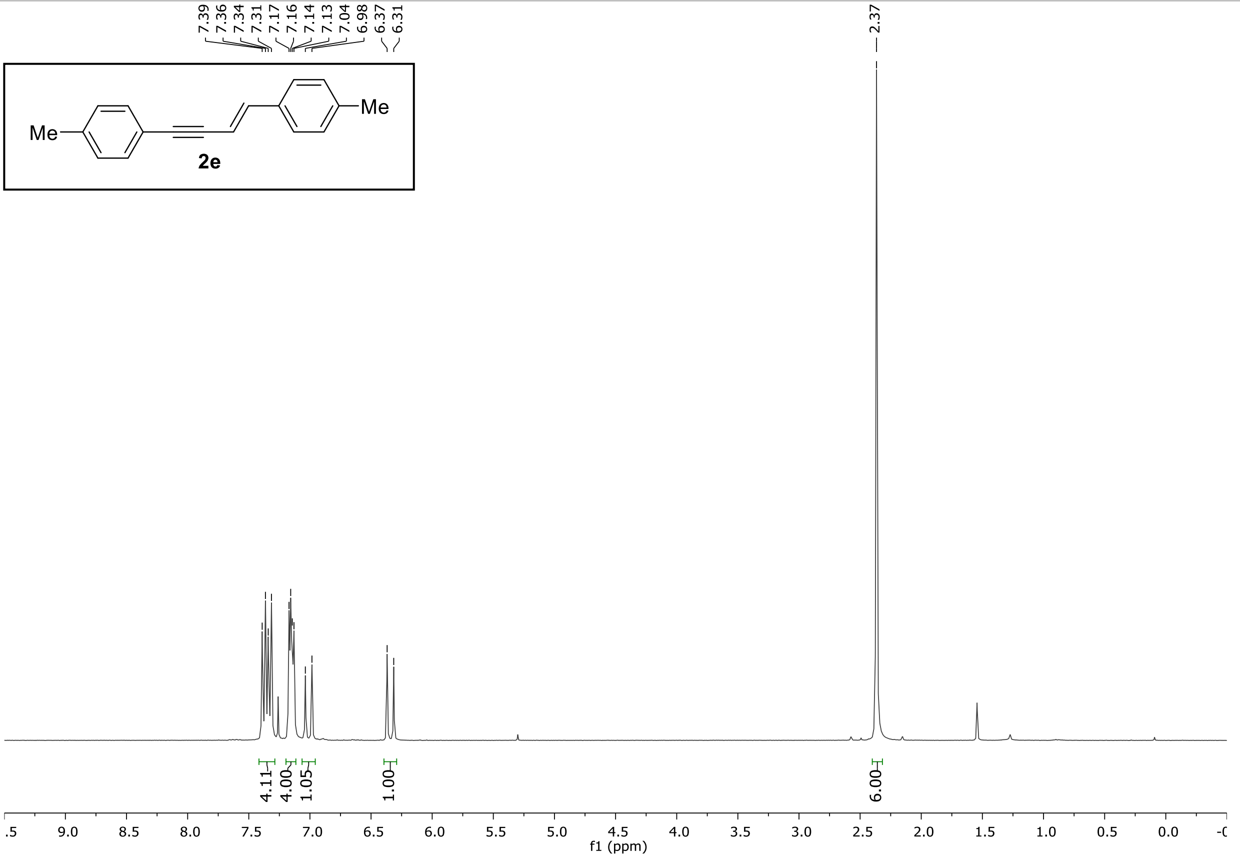
7.39
7.36
7.34
7.31
7.17
7.16
7.14
7.13
7.04
6.98
6.37
6.31
2.37
Me
Me
2e
4.11
4.00
1.05
1.00
6.00
f1 (ppm)

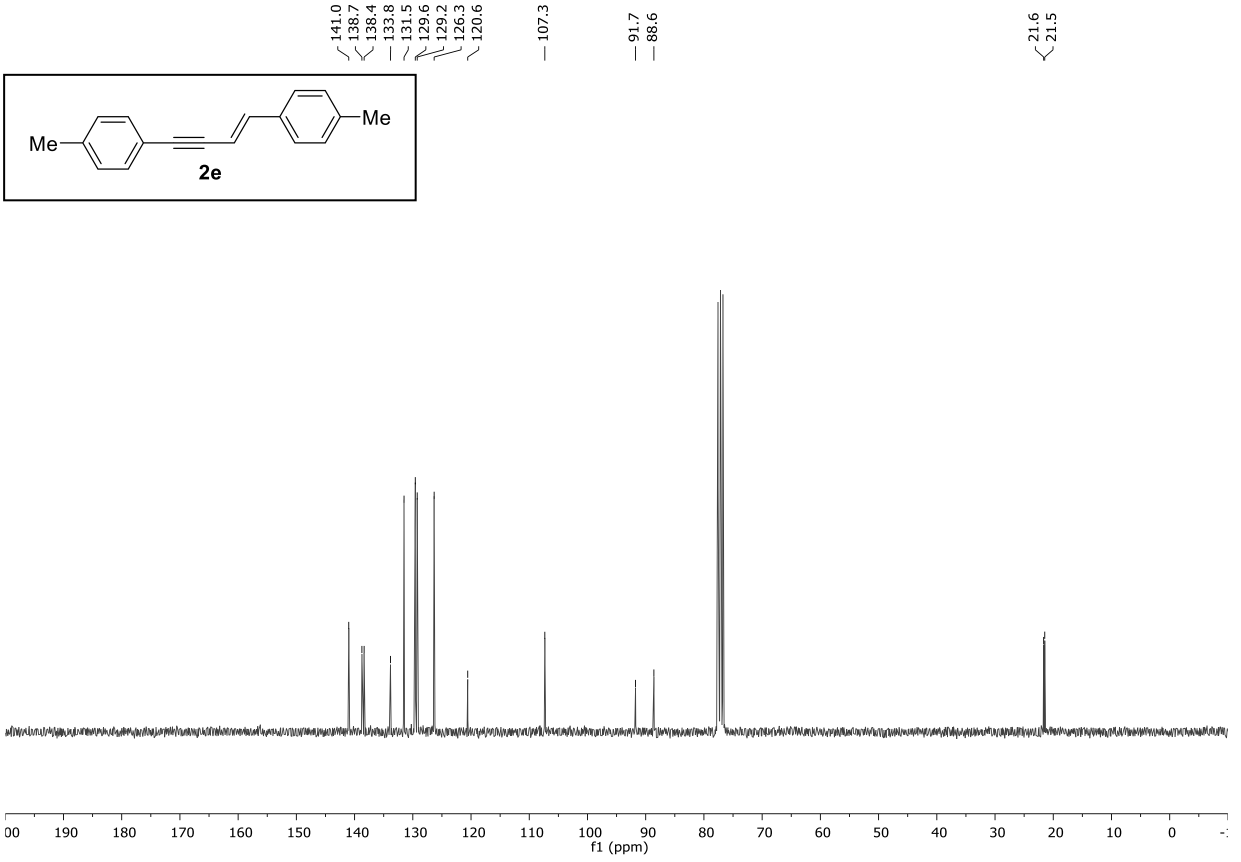
141.0
138.7
138.4
133.8
131.5
129.6
129.2
126.3
120.6
107.3
91.7
88.6
21.6
21.5
Me
Me
2e
f1 (ppm)

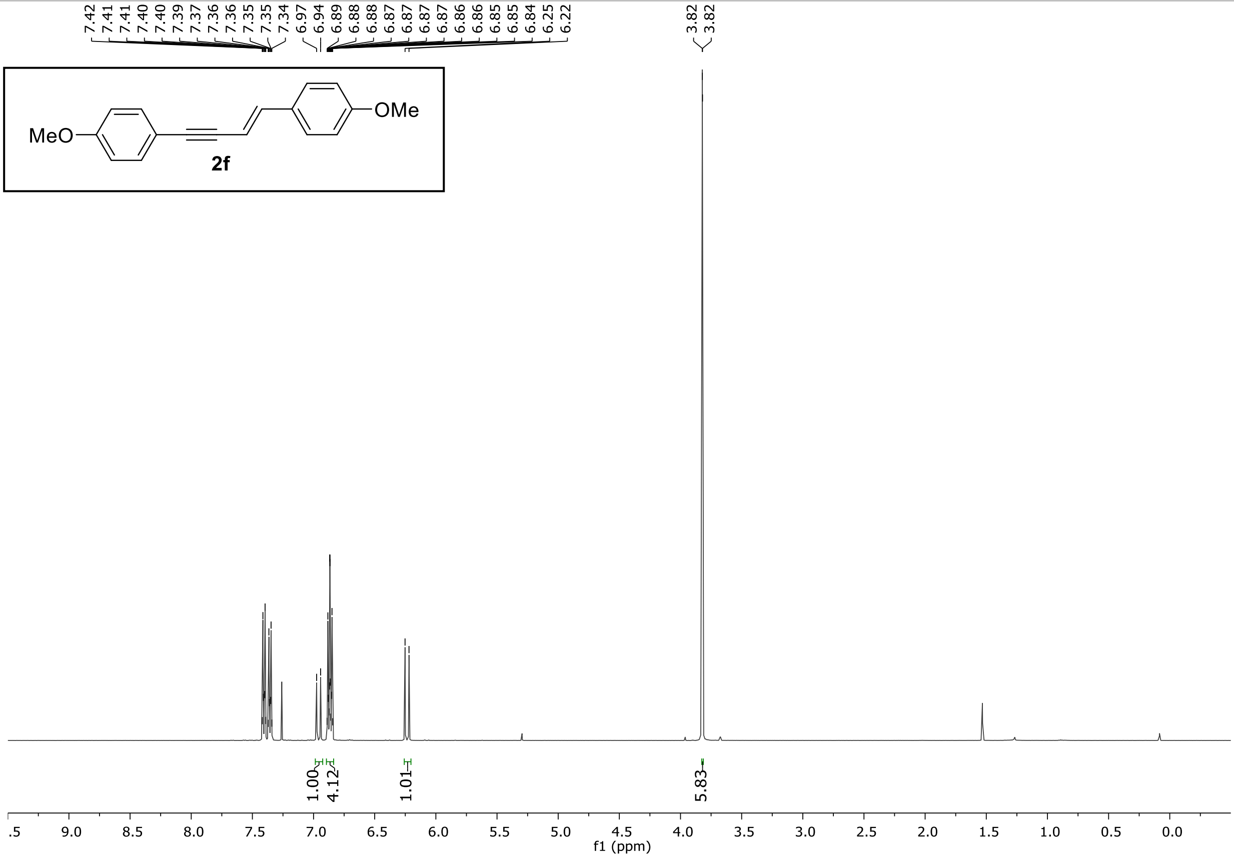
7.42
7.41
7.41
7.40
7.40
7.39
7.37
7.36
7.36
7.35
7.35
7.34
6.97
6.94
6.89
6.88
6.88
6.87
6.87
6.87
6.87
6.86
6.86
6.85
6.85
6.84
6.25
6.22
3.82
3.82
MeO
OMe
2f
1.00
4.12
1.01
5.83
f1 (ppm)

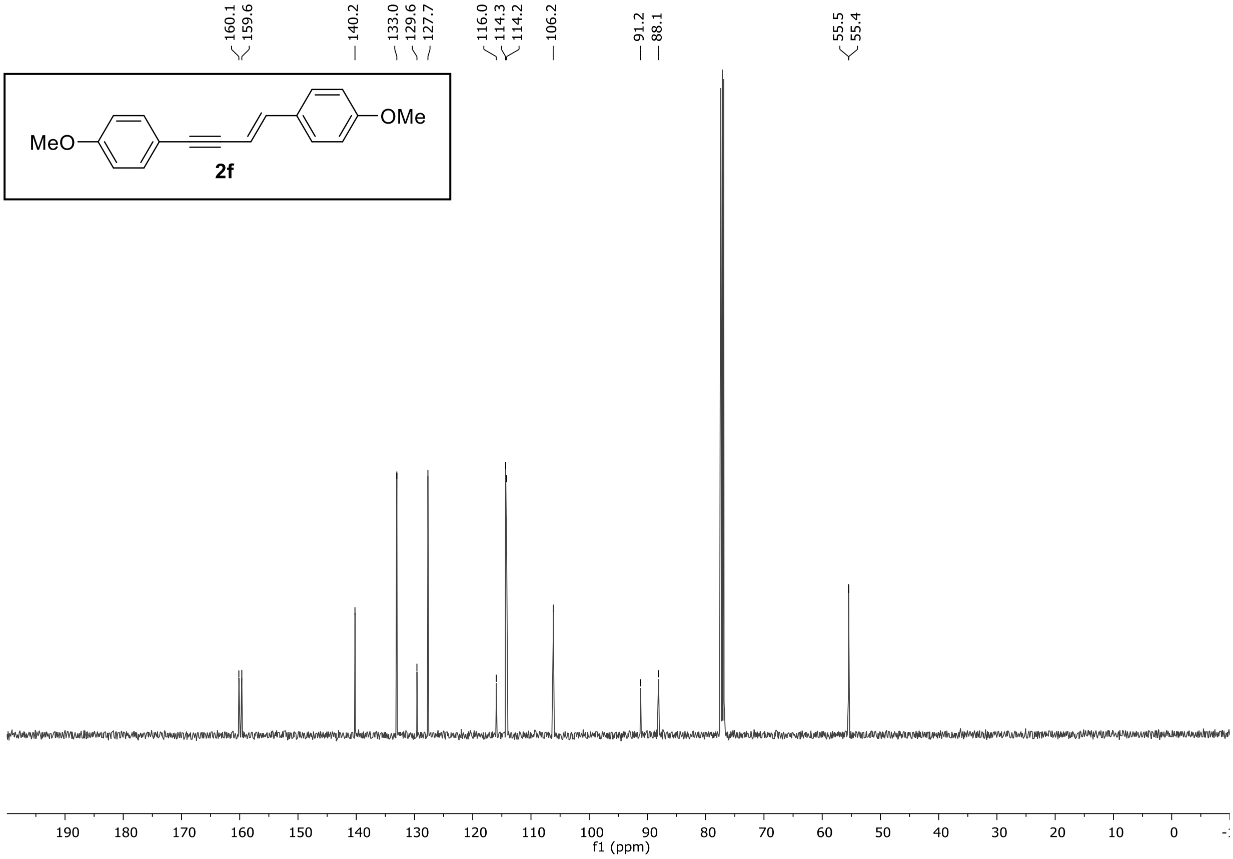
160.1
159.6
140.2
133.0
129.6
127.7
116.0
114.3
114.2
106.2
91.2
88.1
55.5
55.4
MeO
OMe
2f
f1 (ppm)

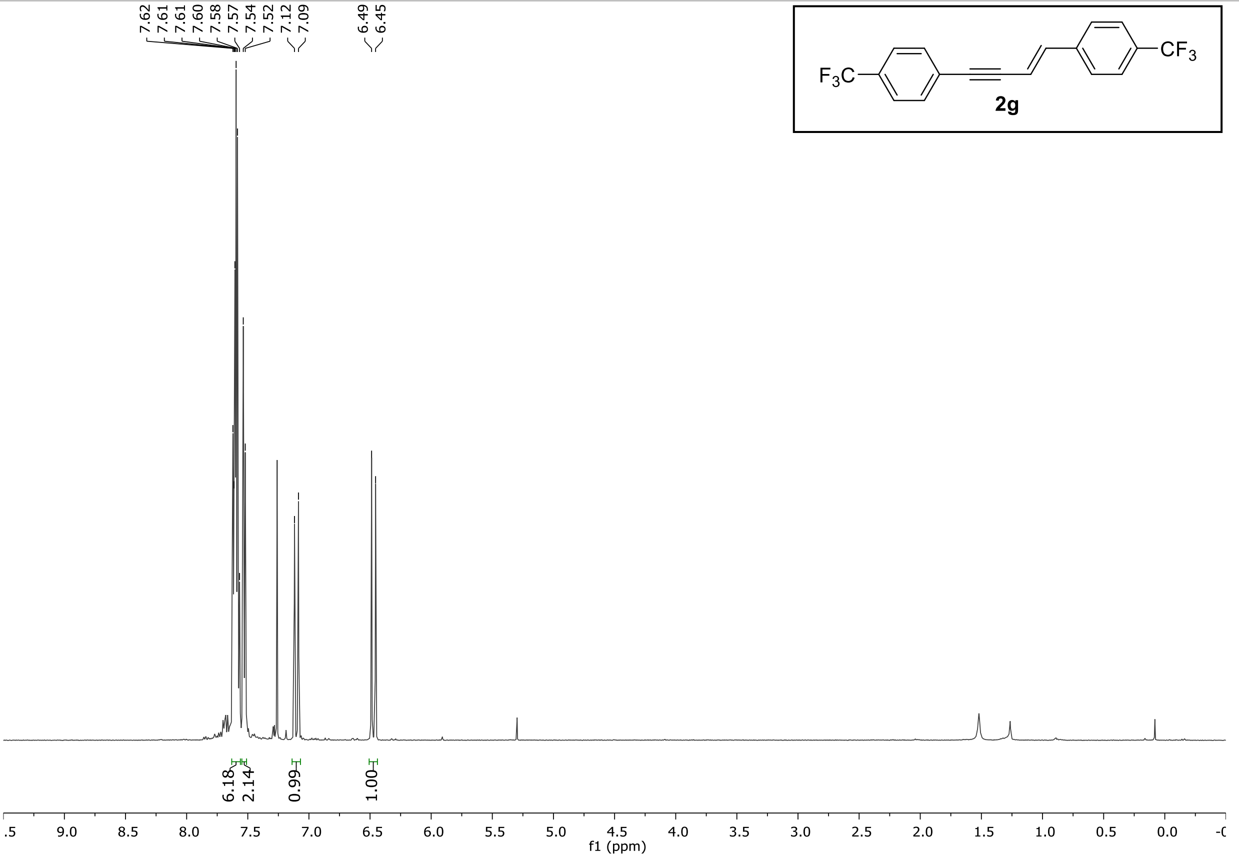
7.62
7.61
7.61
7.60
7.58
7.57
7.54
7.52
7.12
7.09
6.49
6.45
F_3C
CF_3
2g
6.18
2.14
0.99
1.00
f1 (ppm)

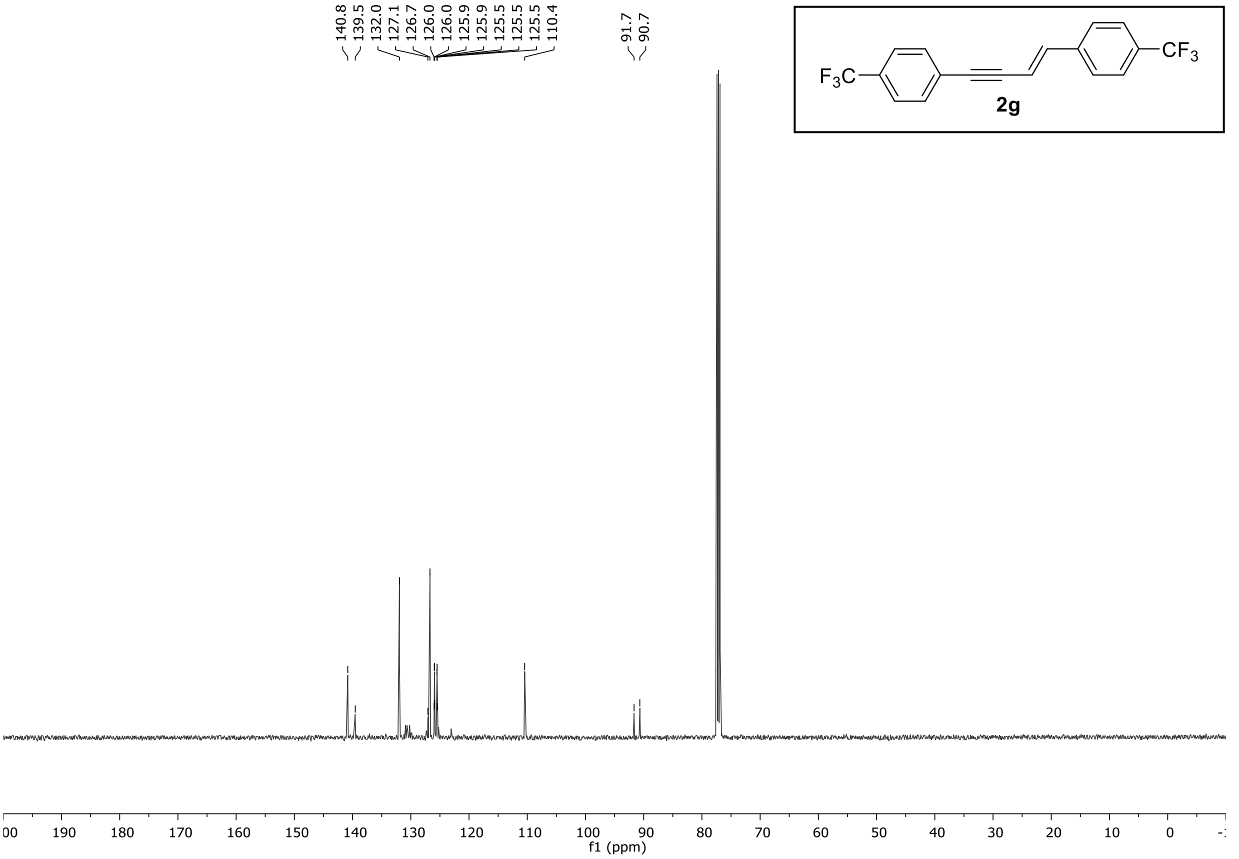
140.8
139.5
132.0
127.1
126.7
126.0
126.0
125.9
125.9
125.5
125.5
125.5
110.4
91.7
90.7
F_3C
CF_3
2g
f1 (ppm)

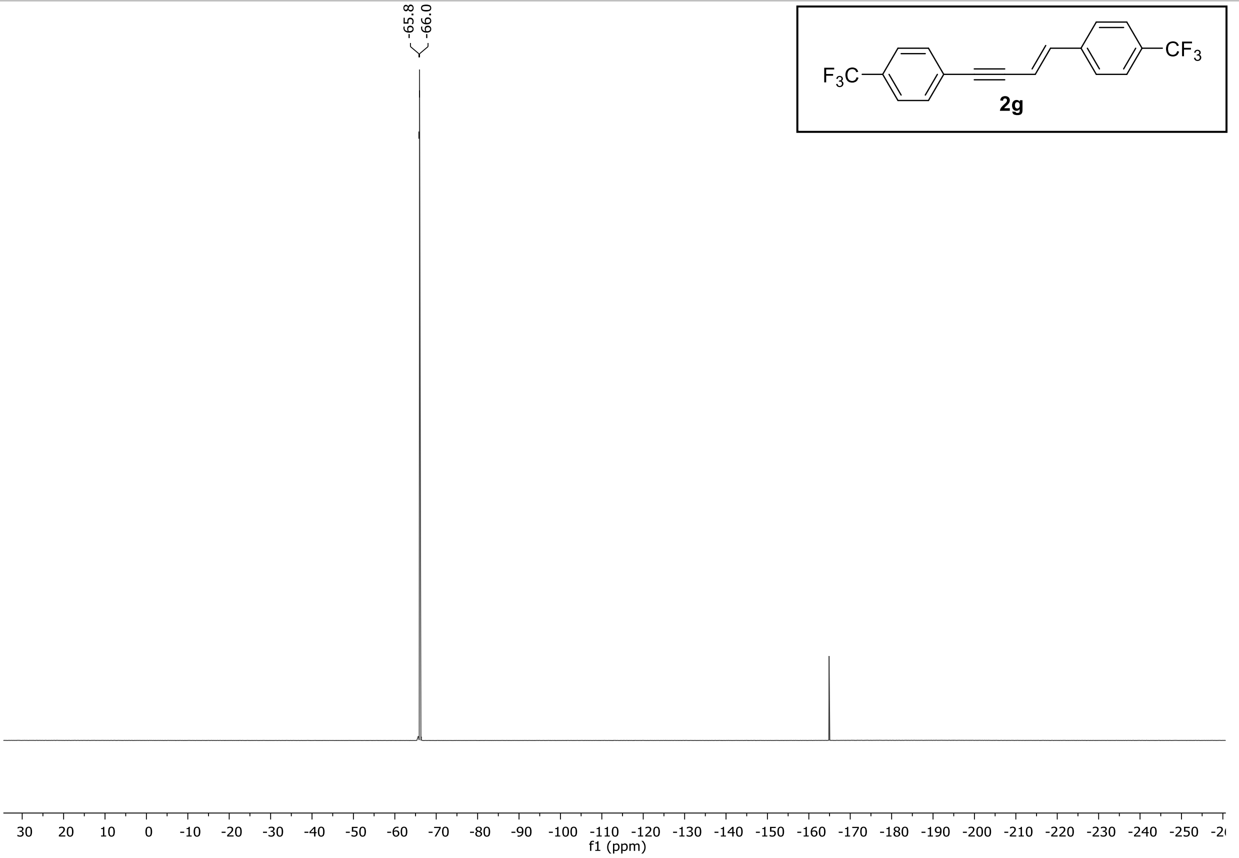
-65.8
-66.0
F3C
CF3
2g
30 20 10 0 -10 -20 -30 -40 -50 -60 -70 -80 -90 -100 -110 -120 -130 -140 -150 -160 -170 -180 -190 -200 -210 -220 -230 -240 -250
f1 (ppm)

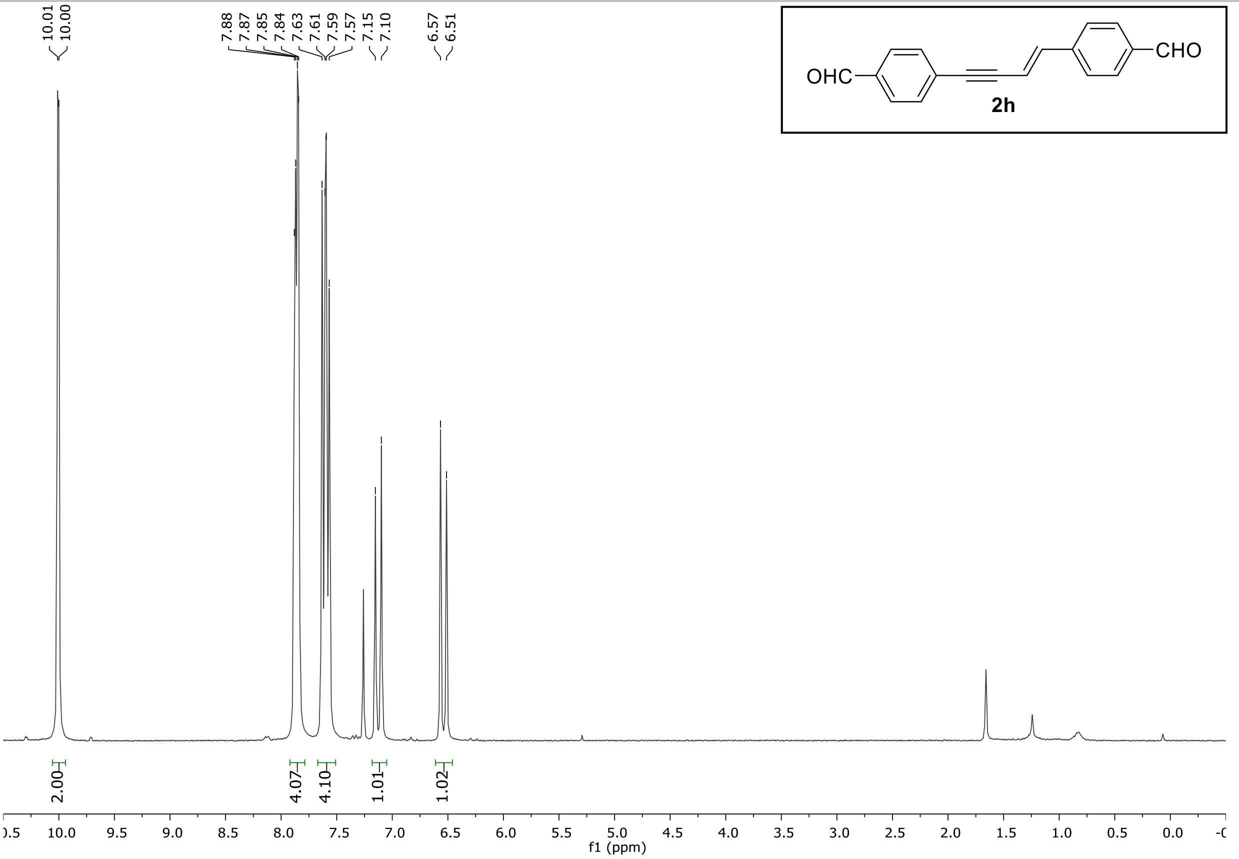
10.01
10.00
7.88
7.87
7.85
7.84
7.63
7.61
7.59
7.57
7.15
7.10
6.57
6.51
OHC
CHO
2h
2.00
4.07
4.10
1.01
1.02
f1 (ppm)

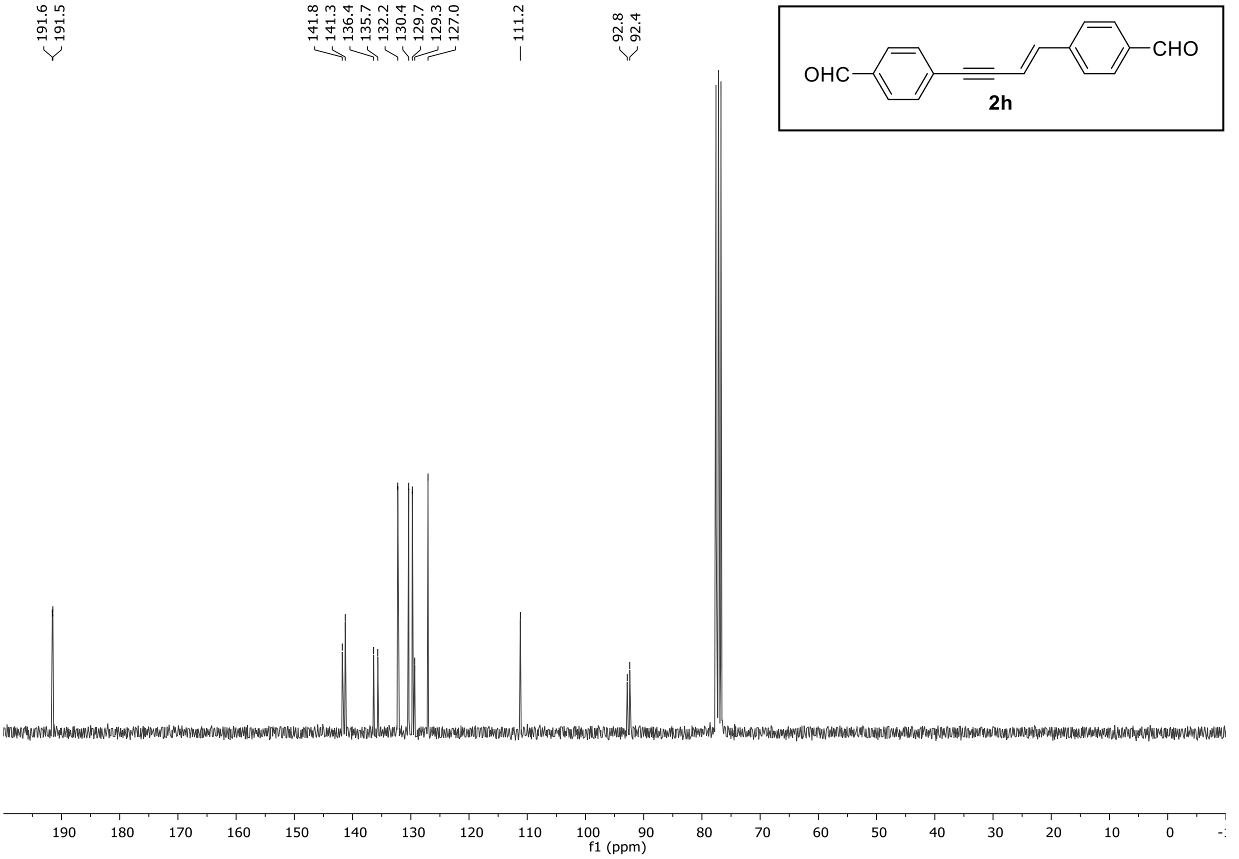
191.6
191.5
141.8
141.3
136.4
135.7
132.2
130.4
129.7
129.3
127.0
111.2
92.8
92.4
OHC
CHO
2h
f1 (ppm)

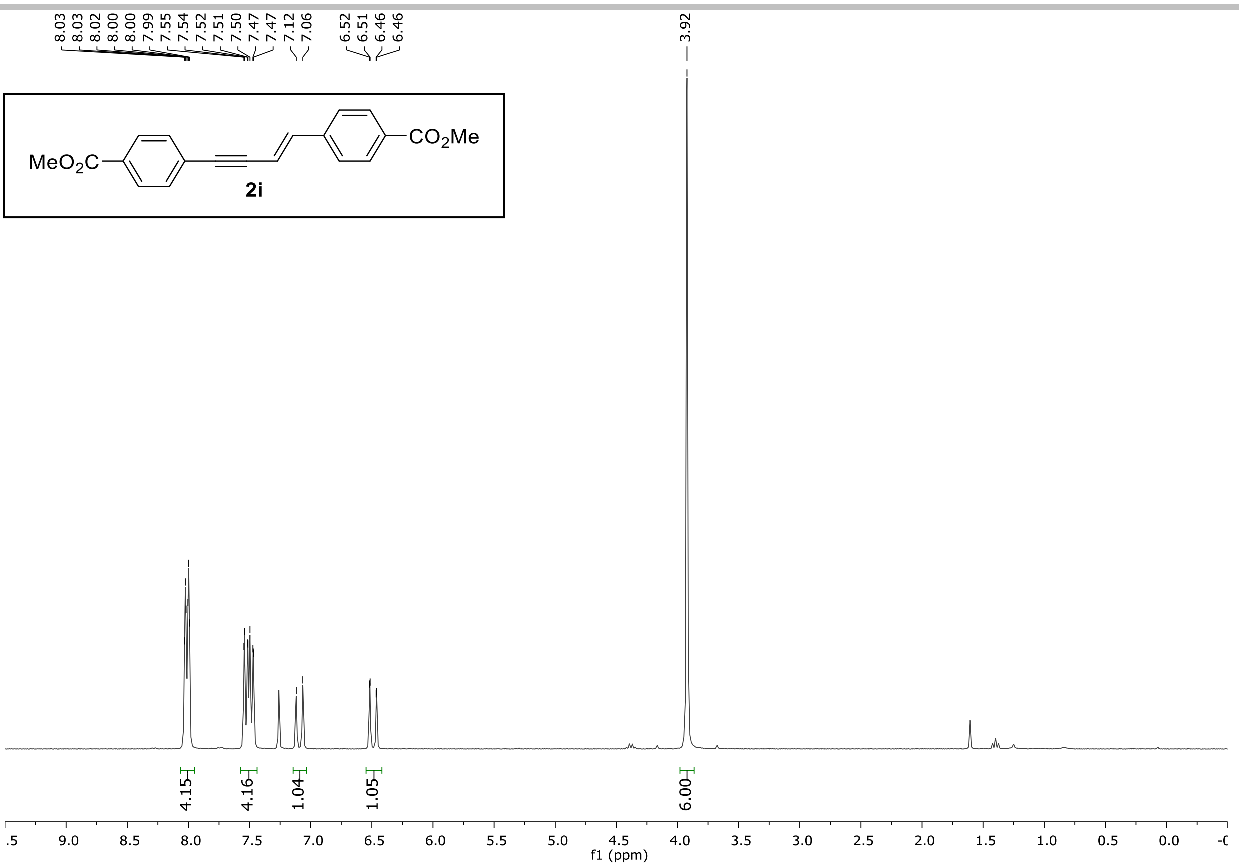
8.03 8.03 8.02 8.00 8.00 7.99 7.55 7.54 7.52 7.51 7.50 7.47 7.47 7.12 7.06 6.52 6.51 6.46 6.46
3.92
MeO2C
CO2Me
2i
4.15
4.16
1.04
1.05
6.00
f1 (ppm)

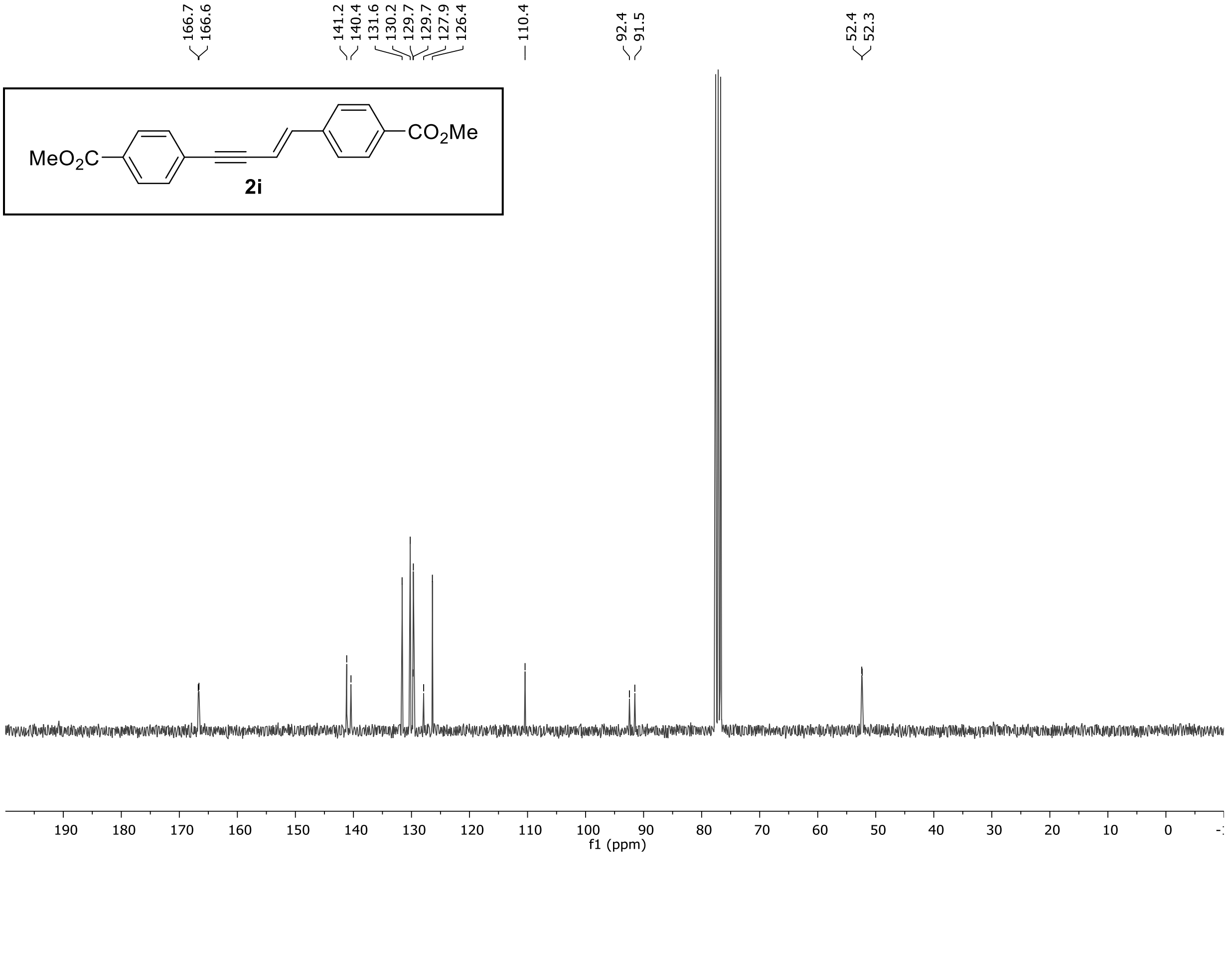
166.7 166.6
141.2 140.4 131.6 130.2 129.7 129.7 127.9 126.4
110.4
92.4 91.5
52.4 52.3
MeO2C
CO2Me
2i
f1 (ppm)

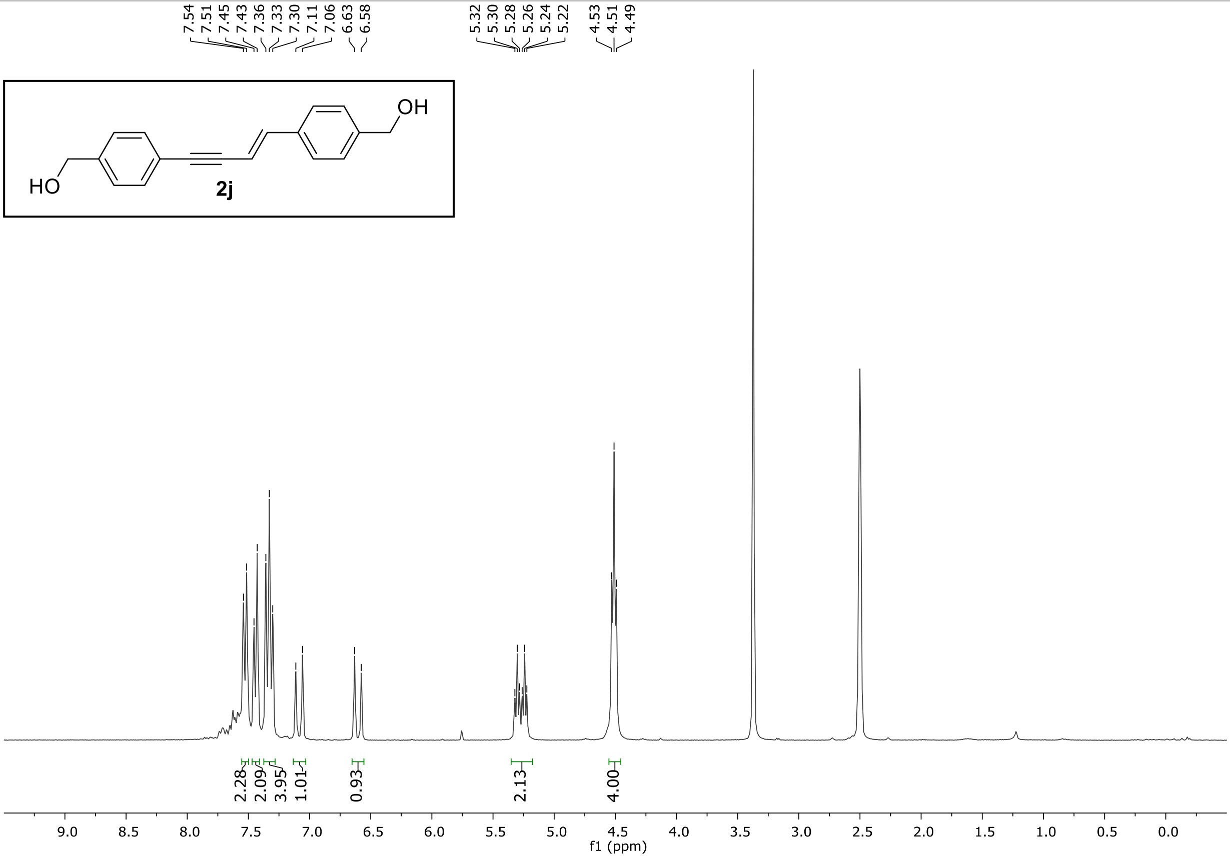
7.54
7.51
7.45
7.43
7.36
7.33
7.30
7.11
7.06
6.63
6.58
5.32
5.30
5.28
5.26
5.24
5.22
4.53
4.51
4.49
OH
HO
2j
2.28
2.09
3.95
1.01
0.93
2.13
4.00
f1 (ppm)

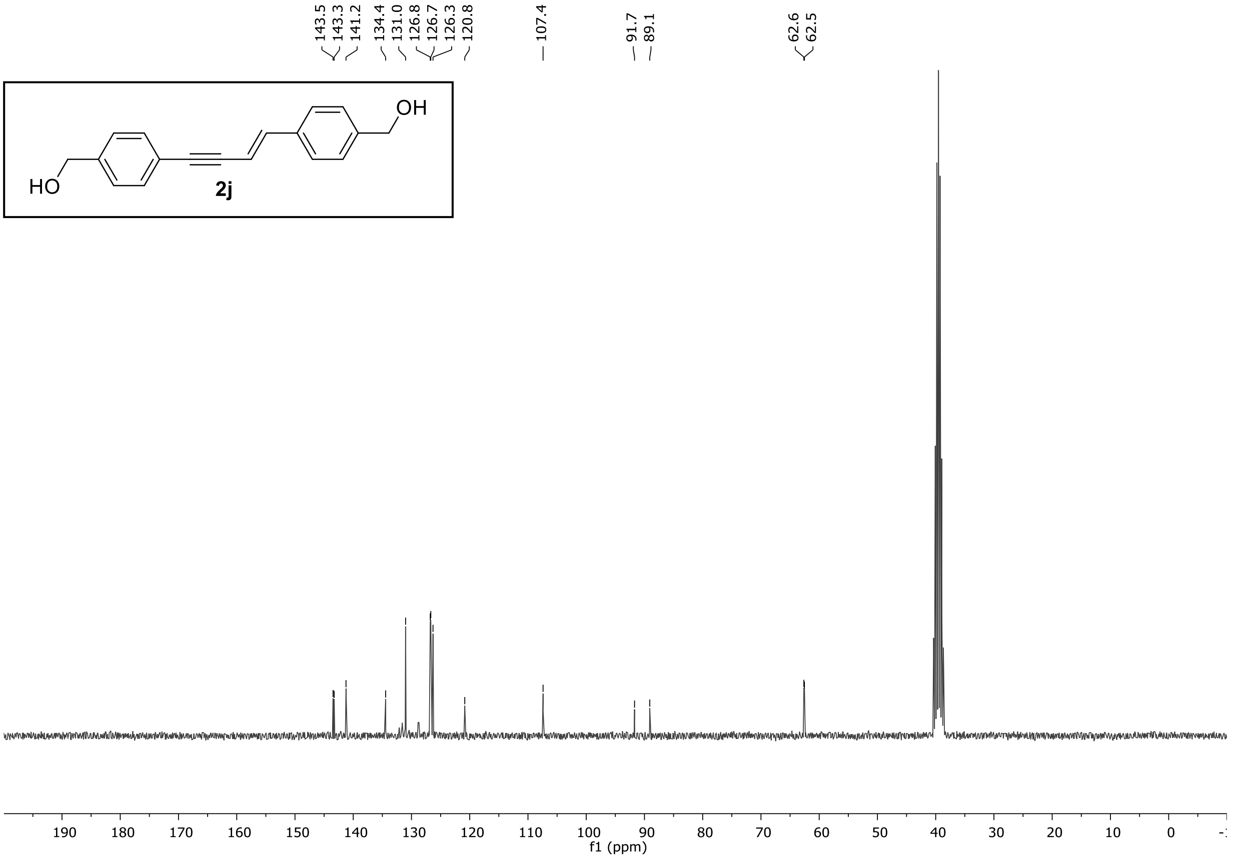
143.5
143.3
141.2
134.4
131.0
126.8
126.7
126.3
120.8
107.4
91.7
89.1
62.6
62.5
OH
HO
2j
f1 (ppm)

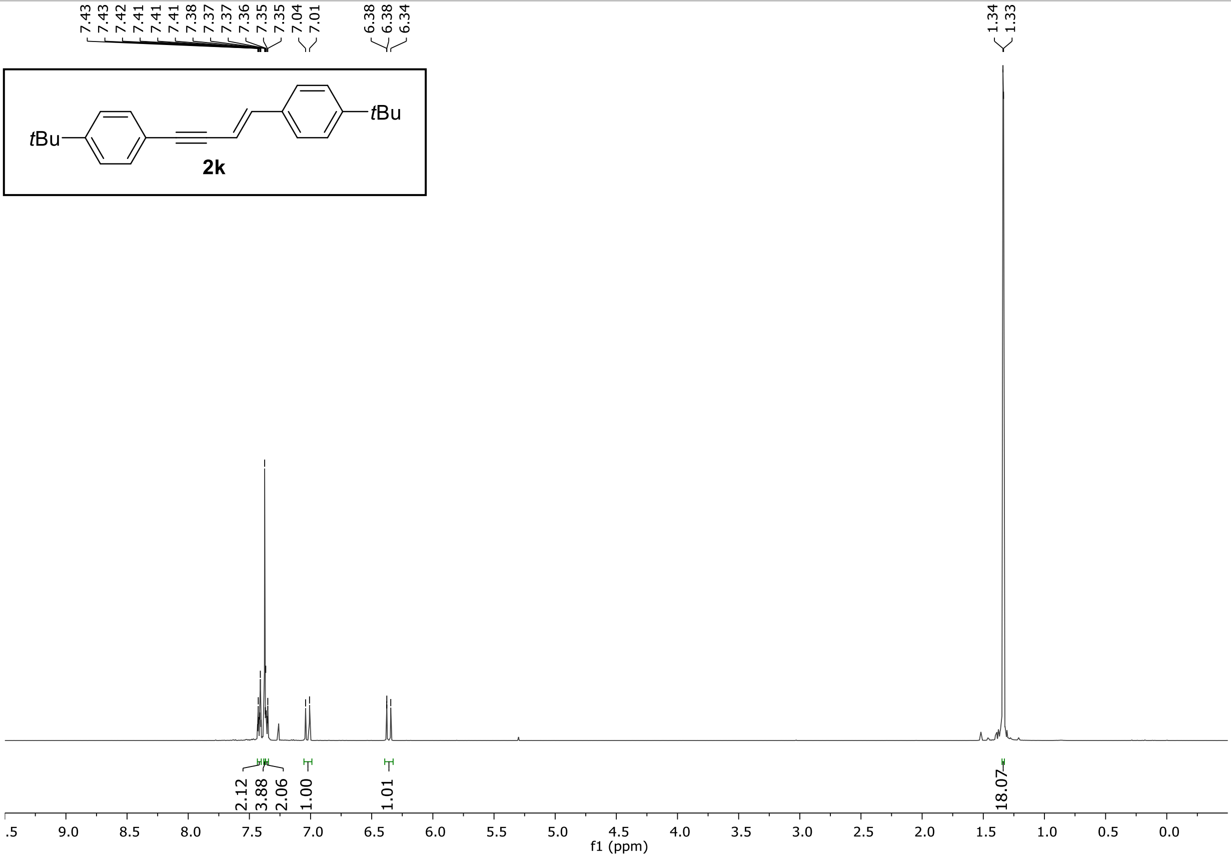
7.43
7.43
7.42
7.41
7.41
7.41
7.38
7.37
7.37
7.36
7.35
7.35
7.04
7.01
6.38
6.38
6.34
1.34
1.33
tBu
tBu
2k
2.12
3.88
2.06
1.00
1.01
18.07
f1 (ppm)

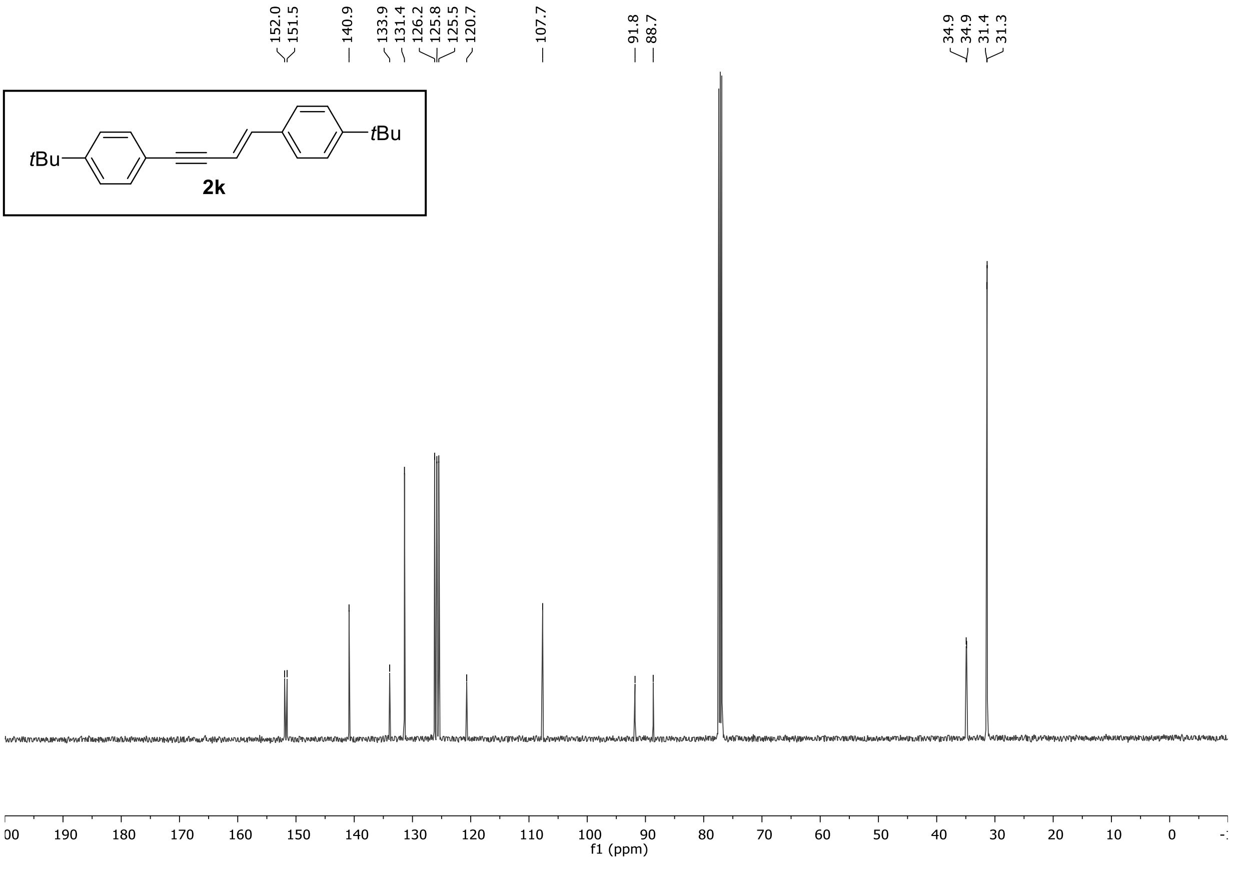
152.0
151.5
140.9
133.9
131.4
126.2
125.8
125.5
120.7
107.7
91.8
88.7
34.9
34.9
31.4
31.3
tBu
tBu
2k
f1 (ppm)

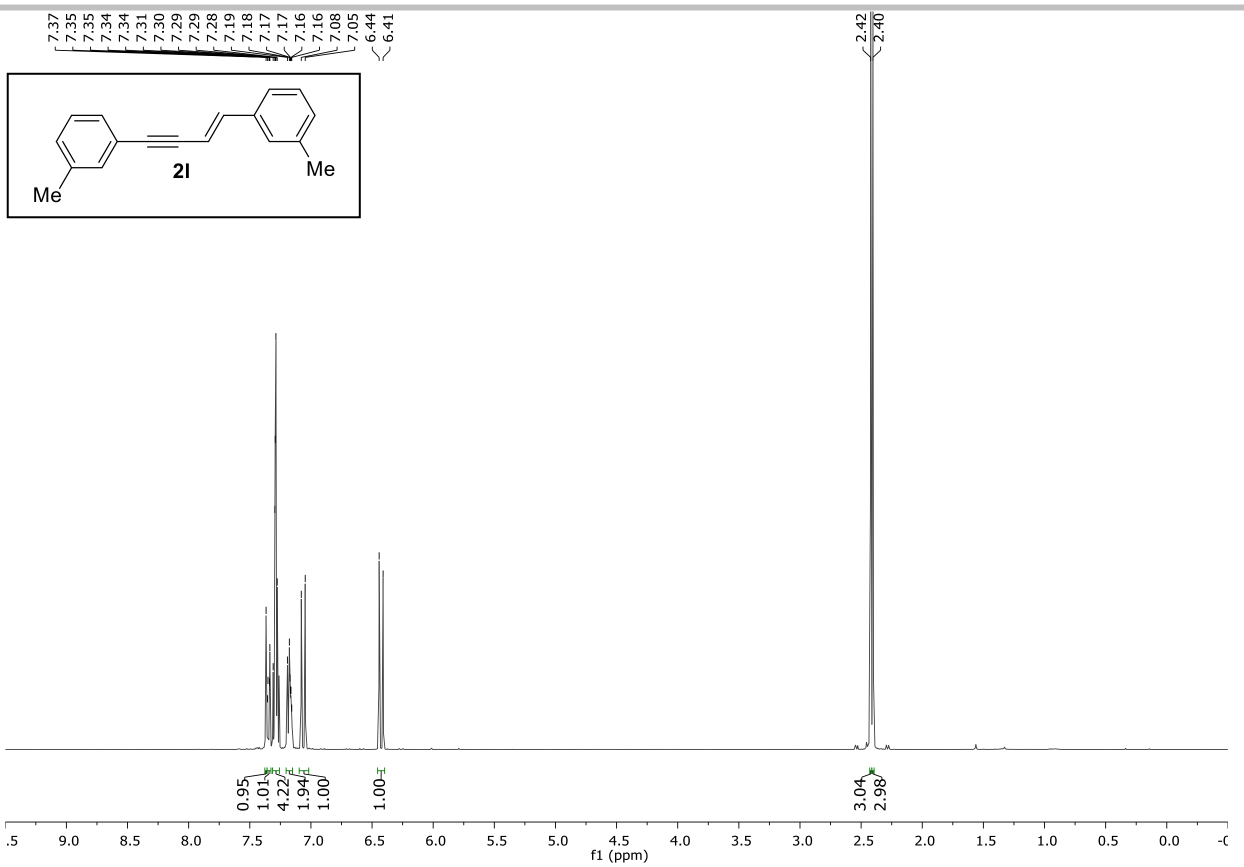
7.37
7.35
7.35
7.34
7.34
7.31
7.30
7.29
7.29
7.28
7.19
7.18
7.17
7.17
7.16
7.16
7.08
7.05
6.44
6.41
2.42
2.40
2l
Me
Me
0.95
1.01
4.22
1.94
1.00
1.00
3.04
2.98
9.0
8.5
8.0
7.5
7.0
6.5
6.0
5.5
5.0
4.5
4.0
3.5
3.0
2.5
2.0
1.5
1.0
0.5
0.0
f1 (ppm)

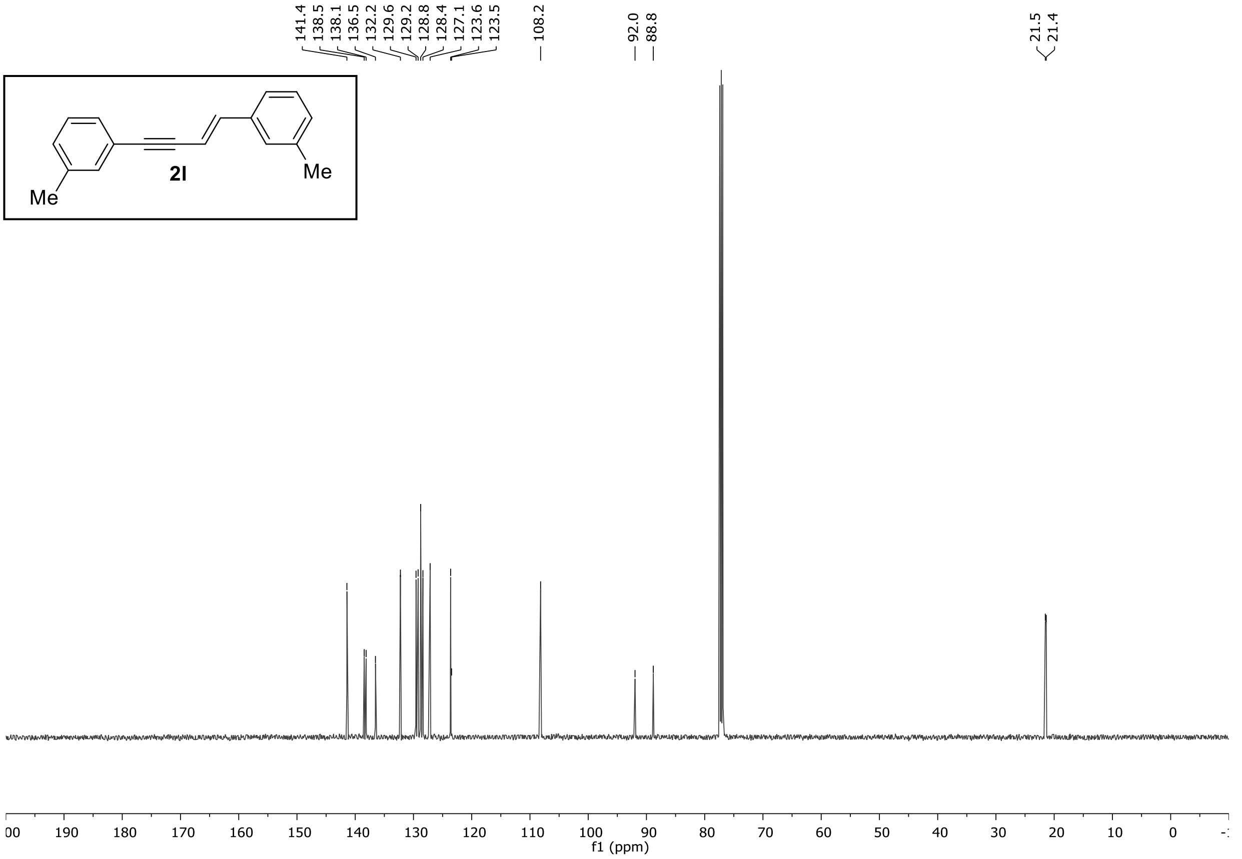
141.4
138.5
138.1
136.5
132.2
129.6
129.2
128.8
128.4
127.1
123.6
123.5
108.2
92.0
88.8
21.5
21.4
2l
Me
Me
190
180
170
160
150
140
130
120
110
100
90
80
70
60
50
40
30
20
10
0
f1 (ppm)

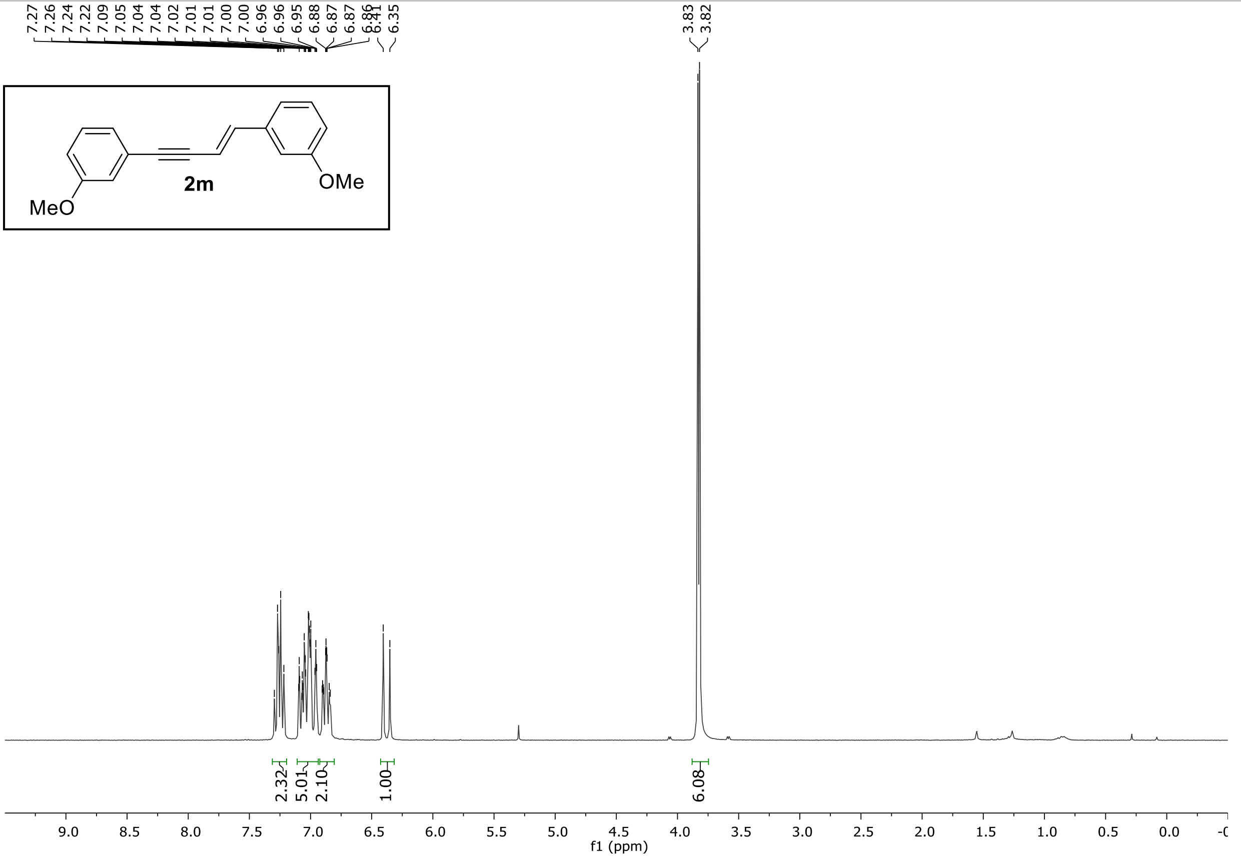
7.27 7.26 7.24 7.22 7.09 7.05 7.04 7.04 7.02 7.01 7.01 7.00 7.00 6.96 6.96 6.95 6.88 6.87 6.87 6.86 6.41 6.35
3.83 3.82
MeO
2m
OMe
2.32
5.01
2.10
1.00
6.08
f1 (ppm)

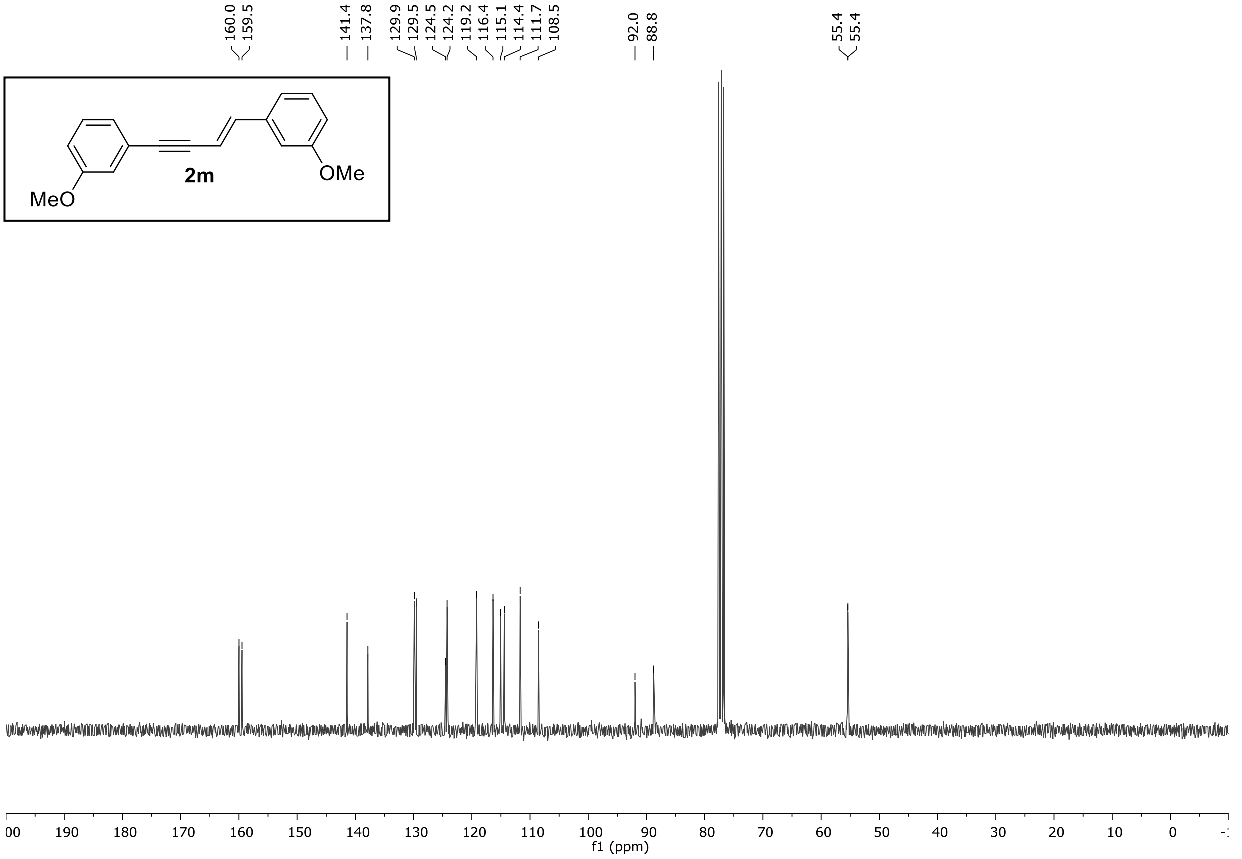
160.0 159.5
141.4 137.8
129.9 129.5 124.5 124.2 119.2 116.4 115.1 114.4 111.7 108.5
92.0 88.8
55.4 55.4
MeO
2m
OMe
f1 (ppm)

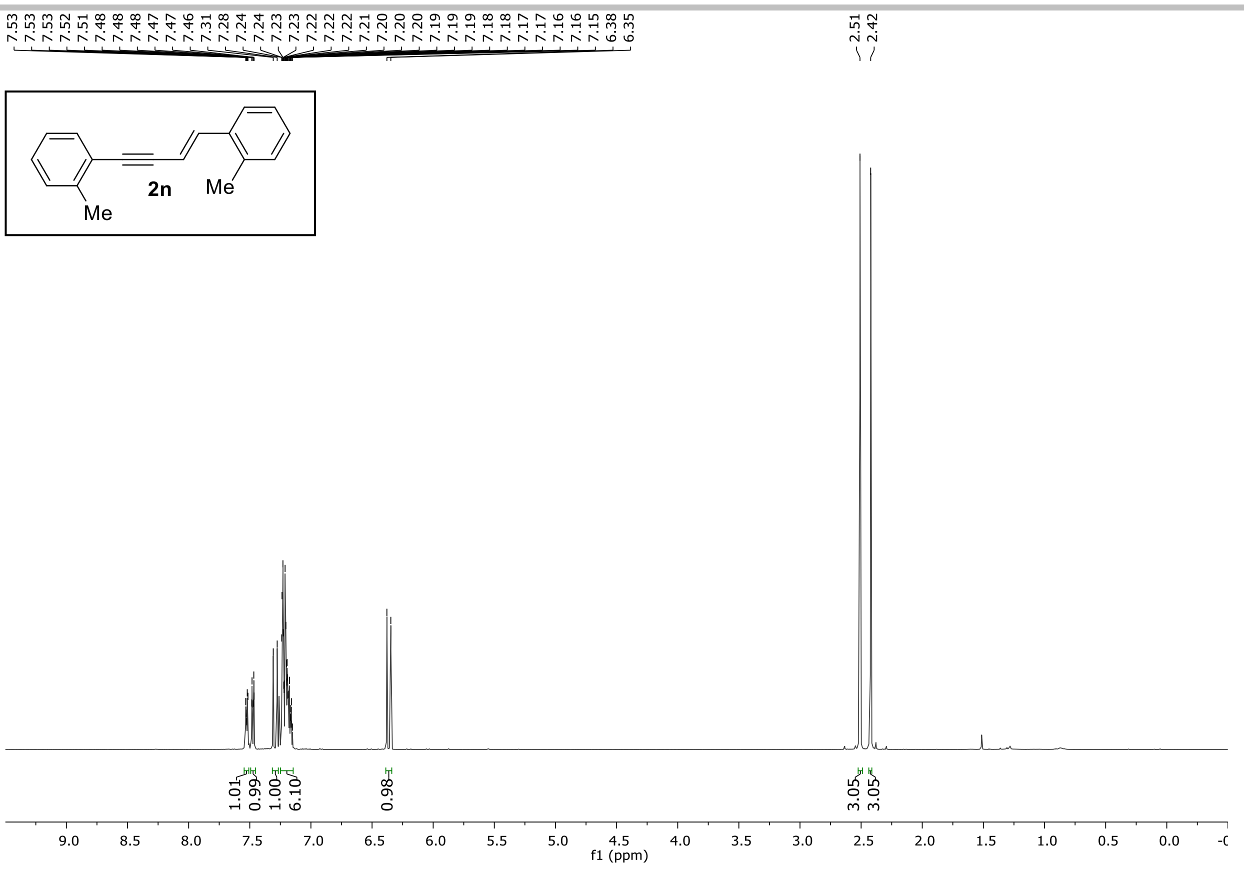
7.53
7.53
7.53
7.52
7.51
7.48
7.48
7.48
7.47
7.47
7.46
7.31
7.28
7.24
7.24
7.23
7.23
7.22
7.22
7.22
7.21
7.21
7.20
7.20
7.20
7.19
7.19
7.19
7.18
7.18
7.17
7.17
7.16
7.16
7.15
6.38
6.35
2.51
2.42
2n
Me
Me
1.01
0.99
1.00
6.10
0.98
3.05
3.05
f1 (ppm)

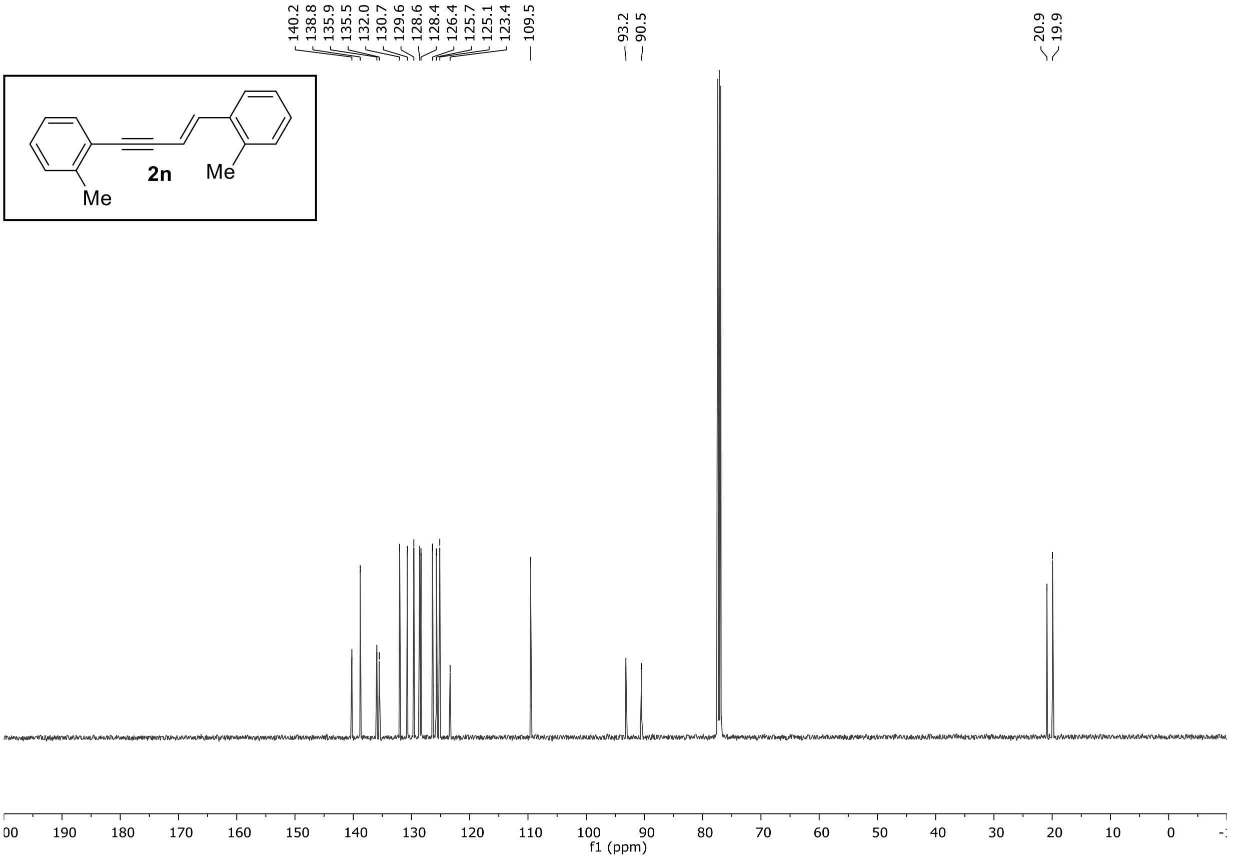
140.2
138.8
135.9
135.5
132.0
130.7
129.6
128.6
128.4
126.4
125.7
125.1
123.4
109.5
93.2
90.5
20.9
19.9
2n
Me
Me
f1 (ppm)

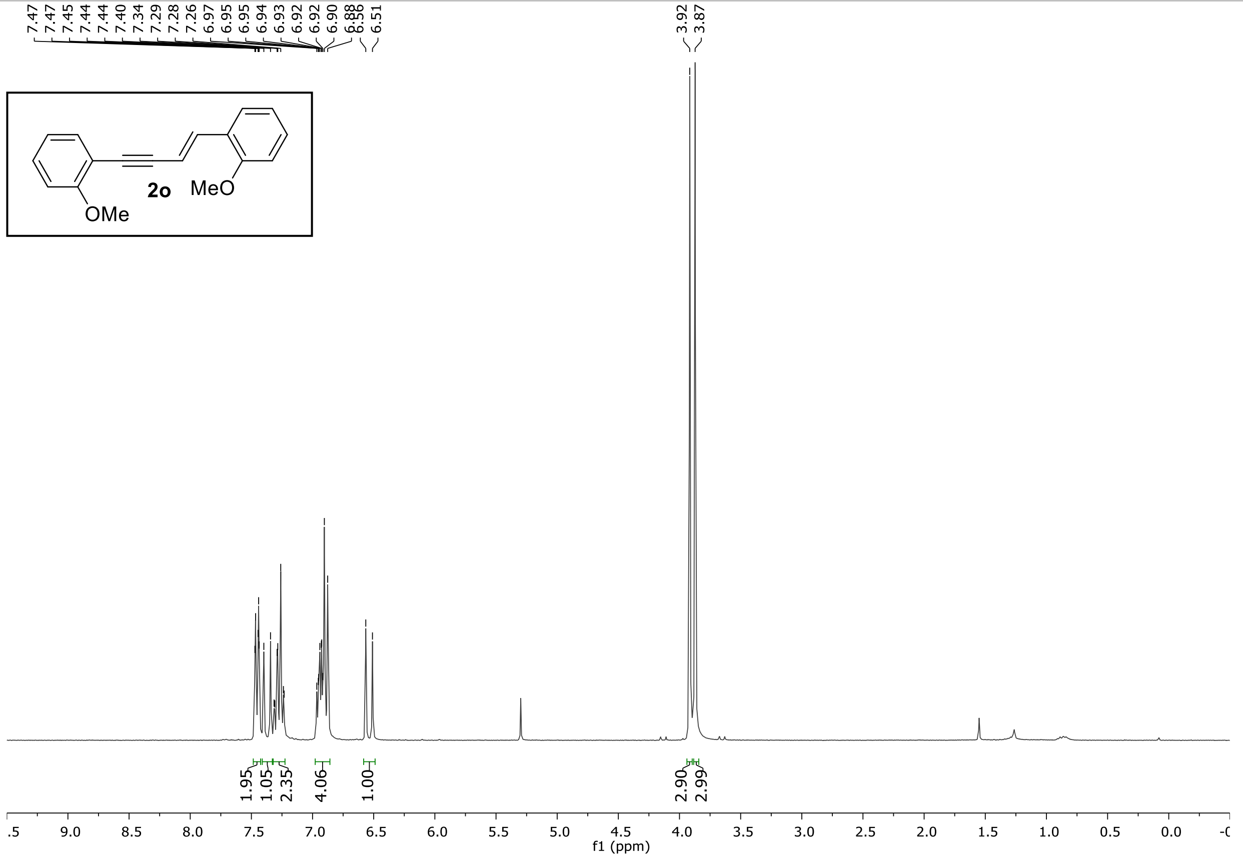
7.47
7.47
7.45
7.44
7.44
7.40
7.34
7.29
7.28
7.26
6.97
6.95
6.95
6.94
6.93
6.92
6.92
6.90
6.88
6.56
6.51
3.92
3.87
2o
MeO
OMe
1.95
1.05
2.35
4.06
1.00
2.90
2.99
f1 (ppm)

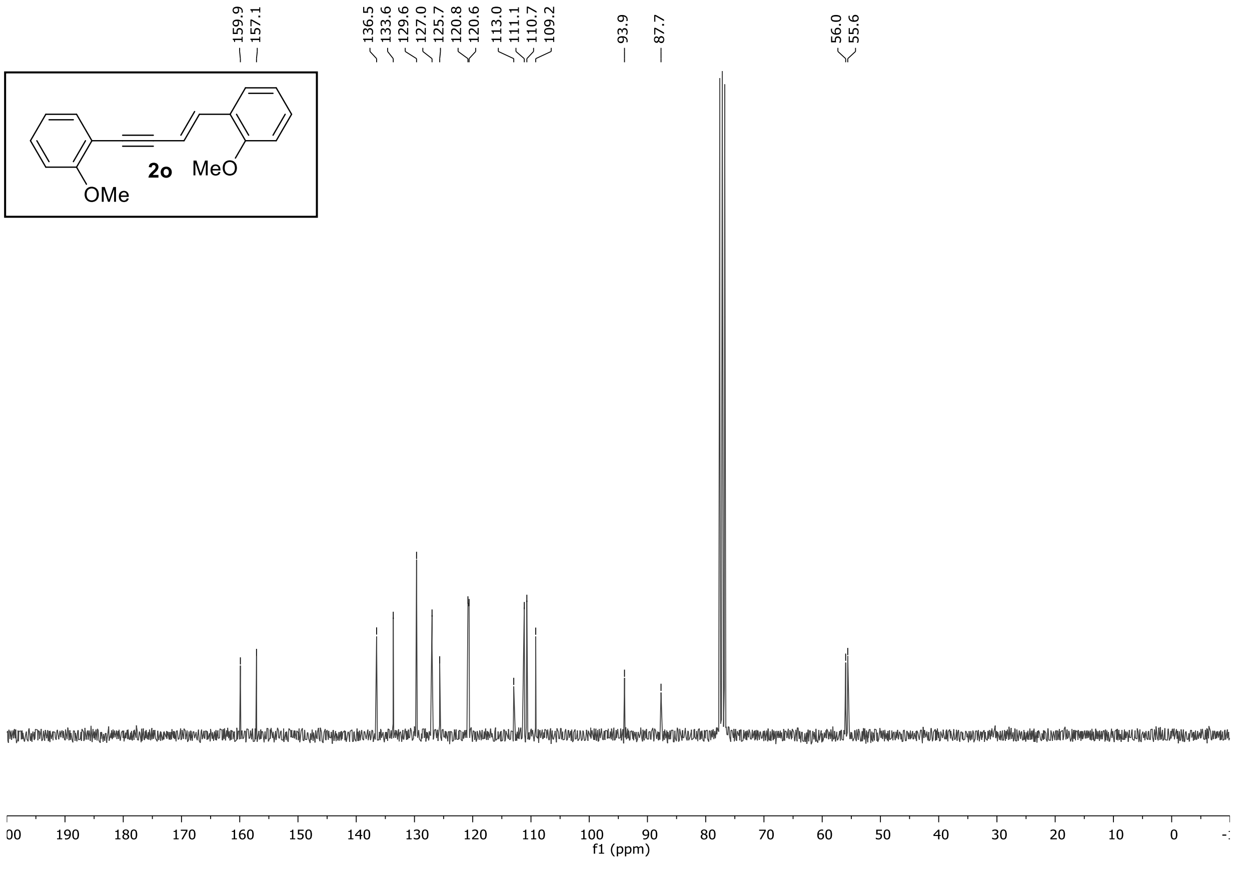
159.9
157.1
136.5
133.6
129.6
127.0
125.7
120.8
120.6
113.0
111.1
110.7
109.2
93.9
87.7
56.0
55.6
2o
MeO
OMe
f1 (ppm)

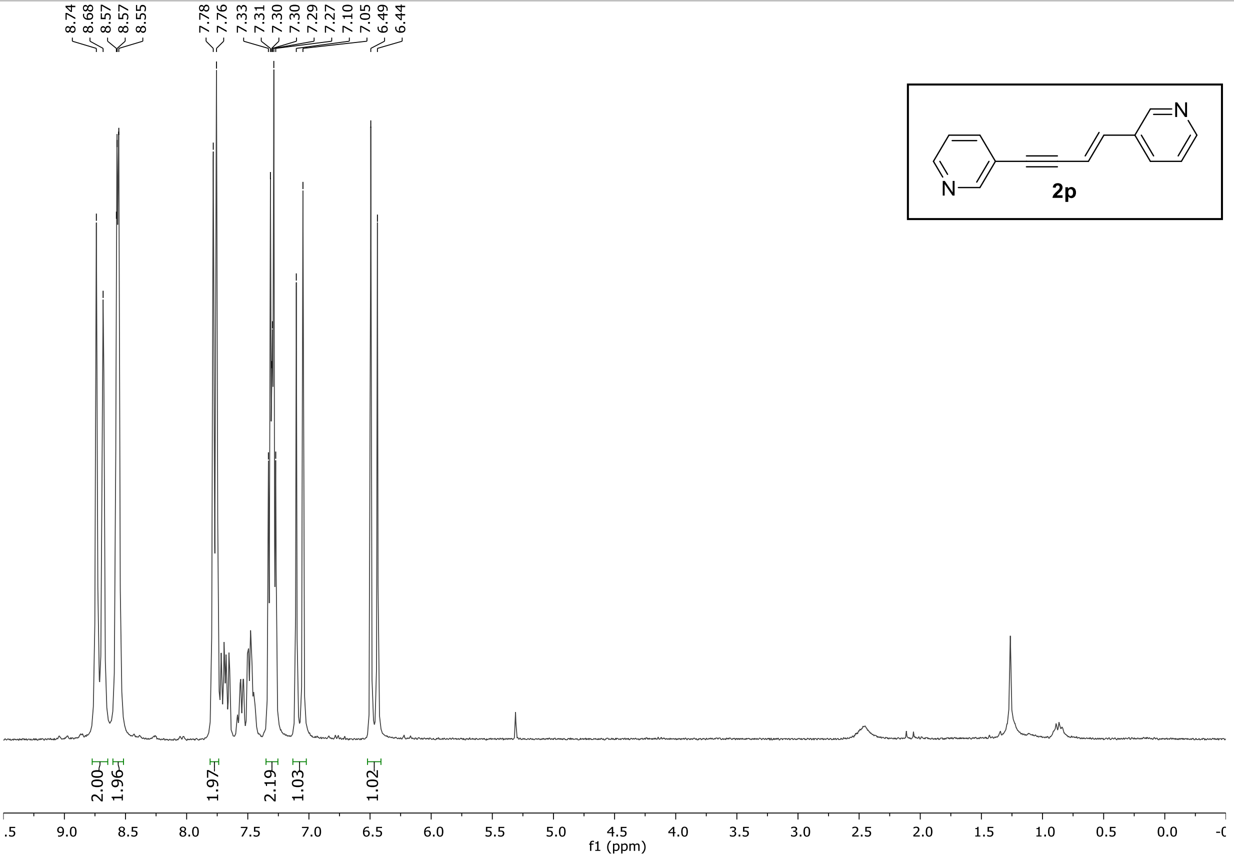
8.74
8.68
8.57
8.57
8.55
7.78
7.76
7.33
7.31
7.30
7.30
7.29
7.27
7.10
7.05
6.49
6.44
N
N
2p
2.00
1.96
1.97
2.19
1.03
1.02
f1 (ppm)

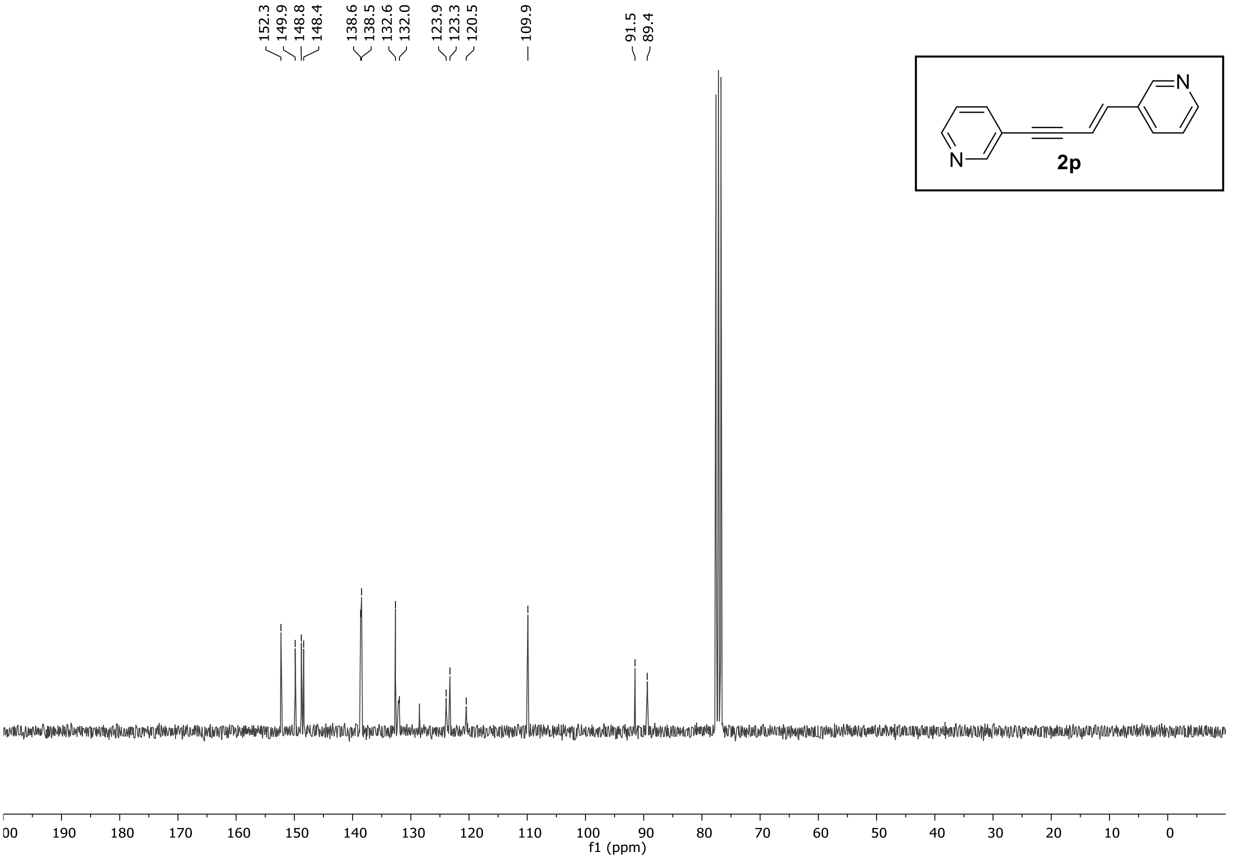
152.3
149.9
148.8
148.4
138.6
138.5
132.6
132.0
123.9
123.3
120.5
109.9
91.5
89.4
N
N
2p
f1 (ppm)

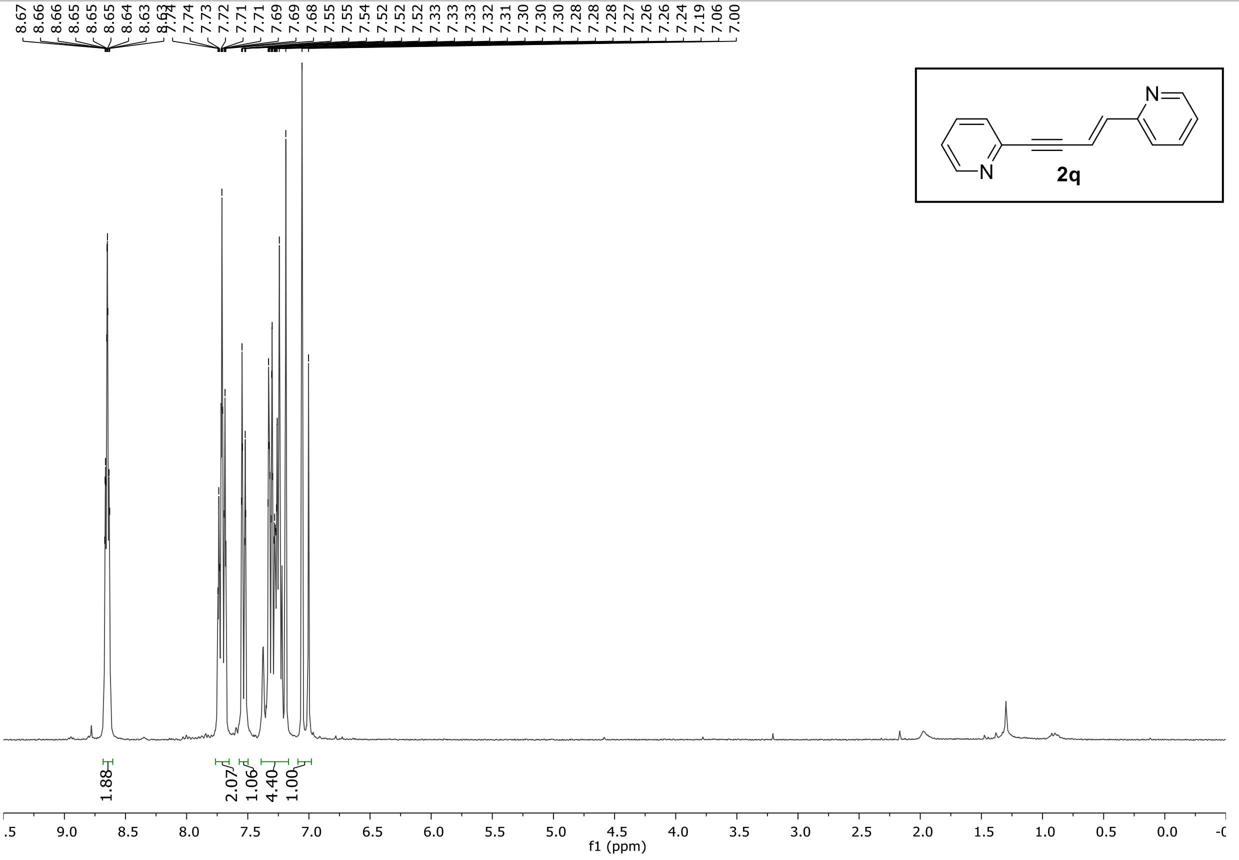
2q
f1 (ppm)

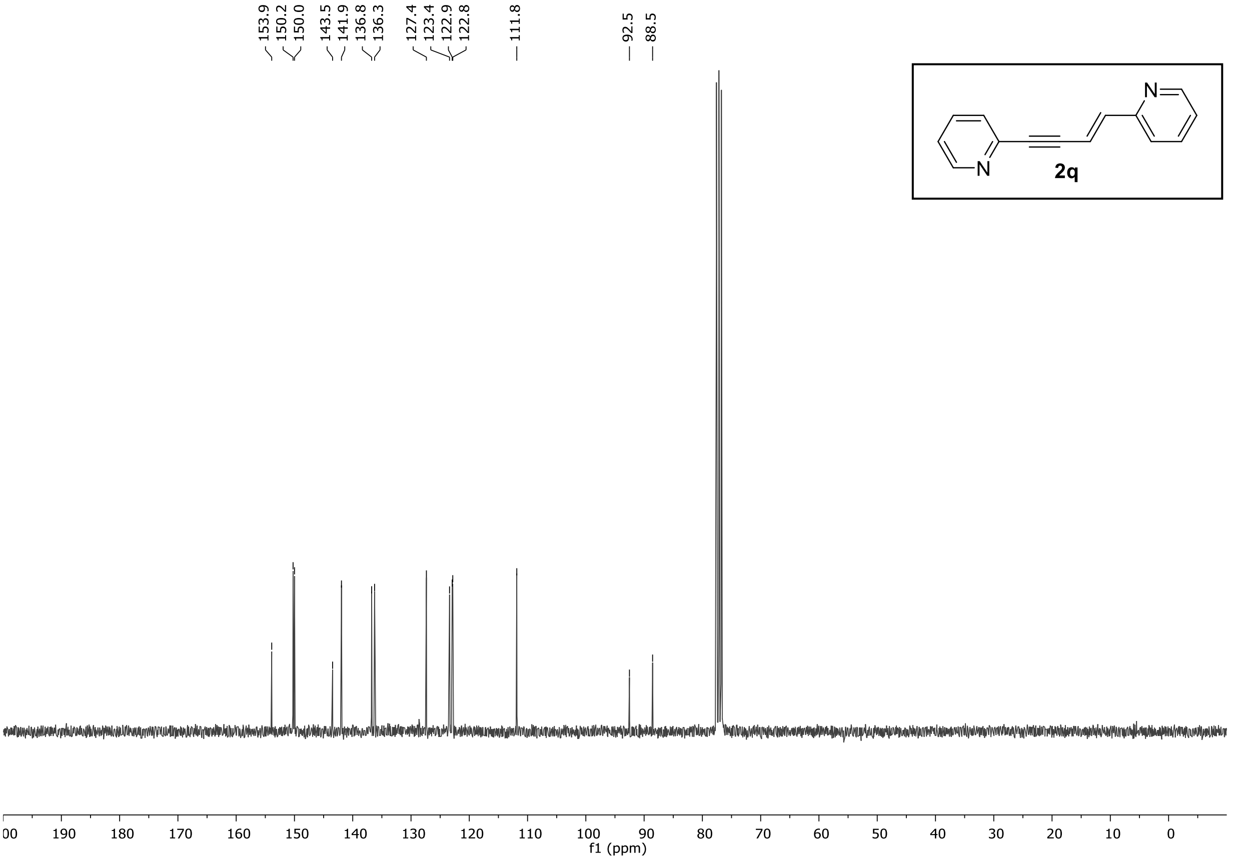
153.9
150.2
150.0
143.5
141.9
136.8
136.3
127.4
123.4
122.9
122.8
111.8
92.5
88.5
2q
f1 (ppm)

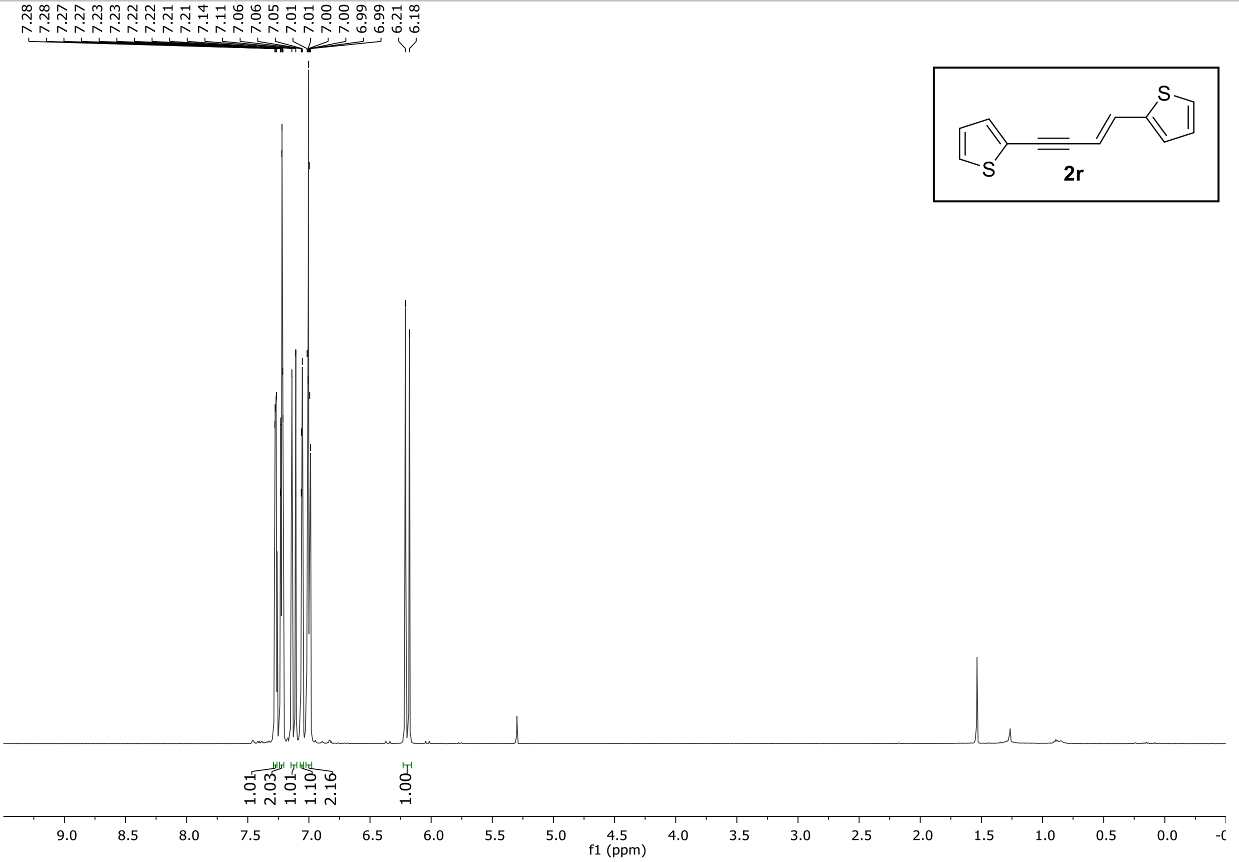
7.28
7.28
7.27
7.27
7.23
7.22
7.22
7.21
7.21
7.14
7.11
7.06
7.06
7.05
7.01
7.01
7.00
7.00
6.99
6.99
6.21
6.18
S
S
2r
1.01
2.03
1.01
1.10
2.16
1.00
f1 (ppm)

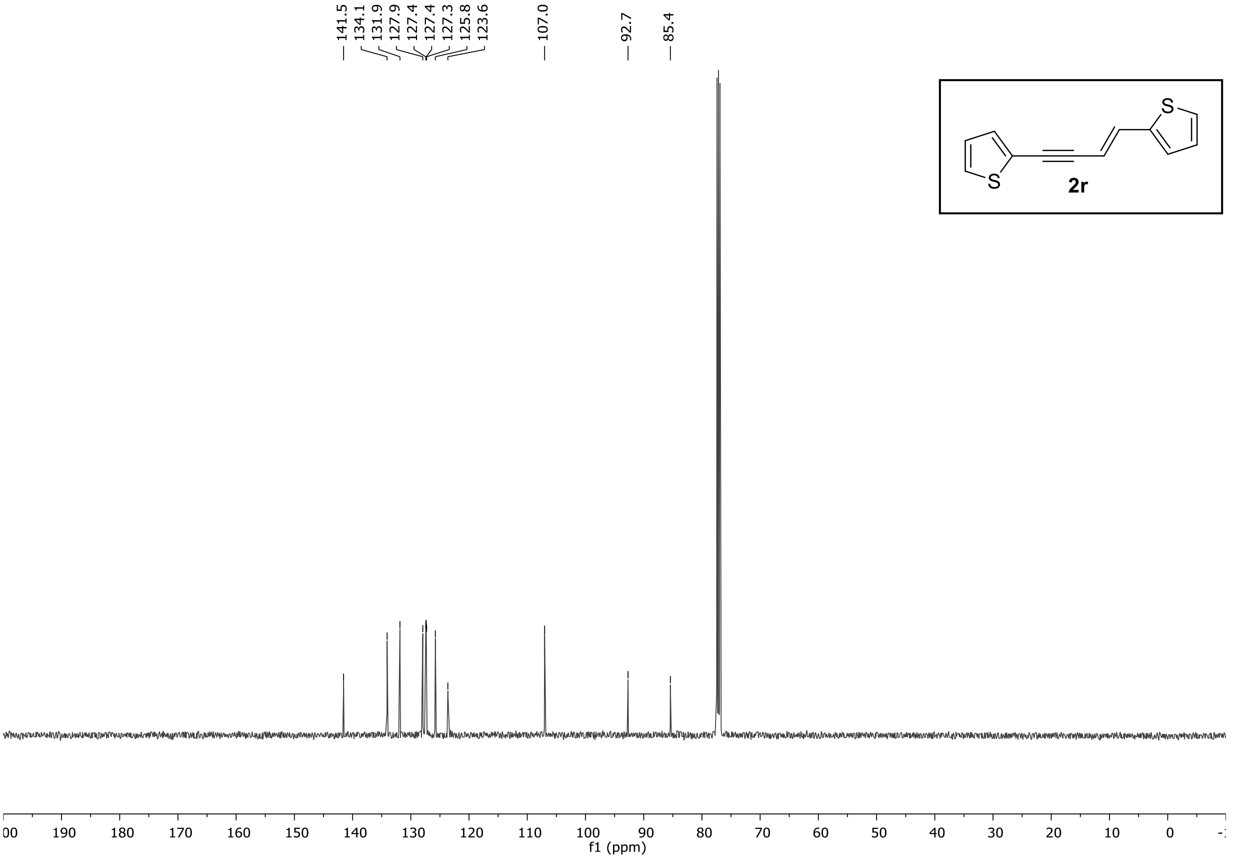
141.5
134.1
131.9
127.9
127.4
127.4
127.3
125.8
123.6
107.0
92.7
85.4
S
S
2r
f1 (ppm)

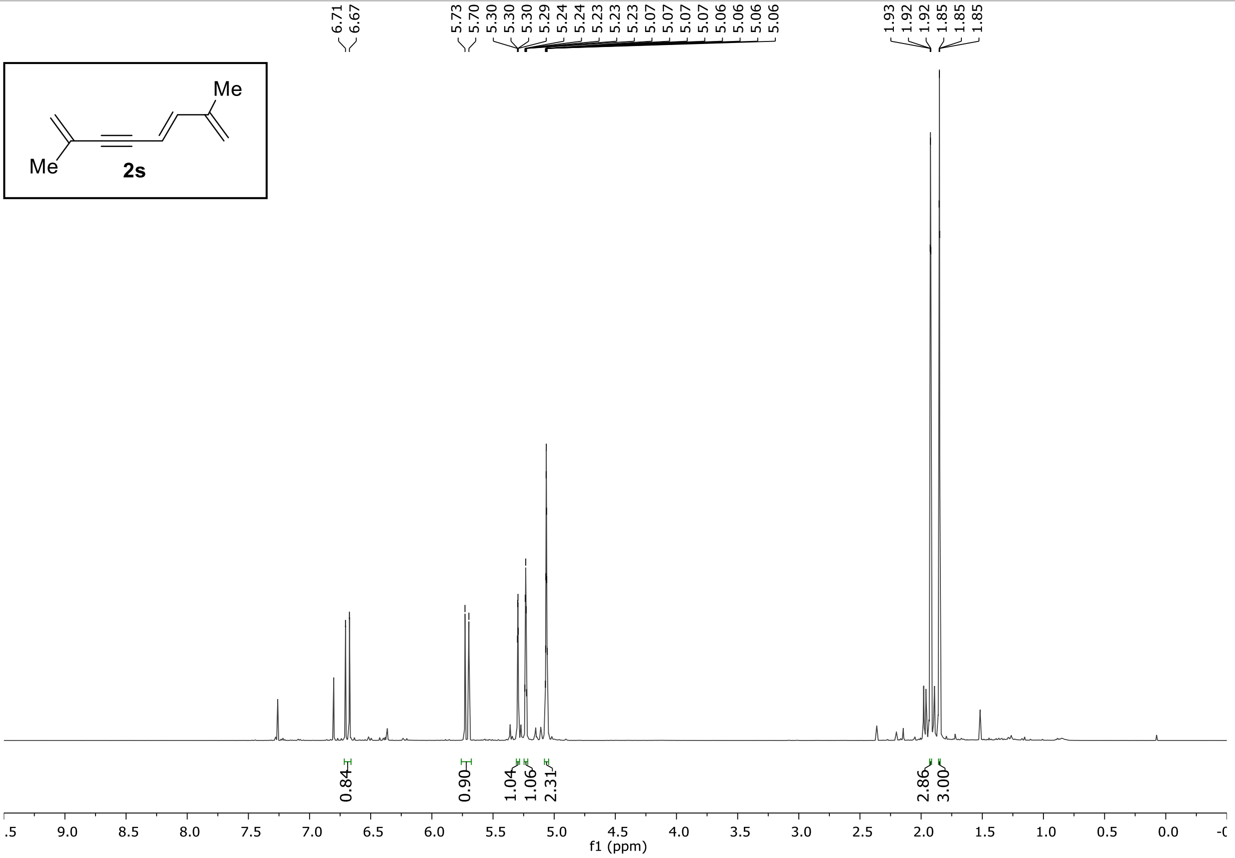
6.71
6.67
5.73
5.70
5.30
5.30
5.30
5.29
5.24
5.24
5.23
5.23
5.23
5.07
5.07
5.07
5.07
5.06
5.06
5.06
5.06
1.93
1.92
1.92
1.85
1.85
1.85
Me
Me
2s
0.84
0.90
1.04
1.06
2.31
2.86
3.00
f1 (ppm)

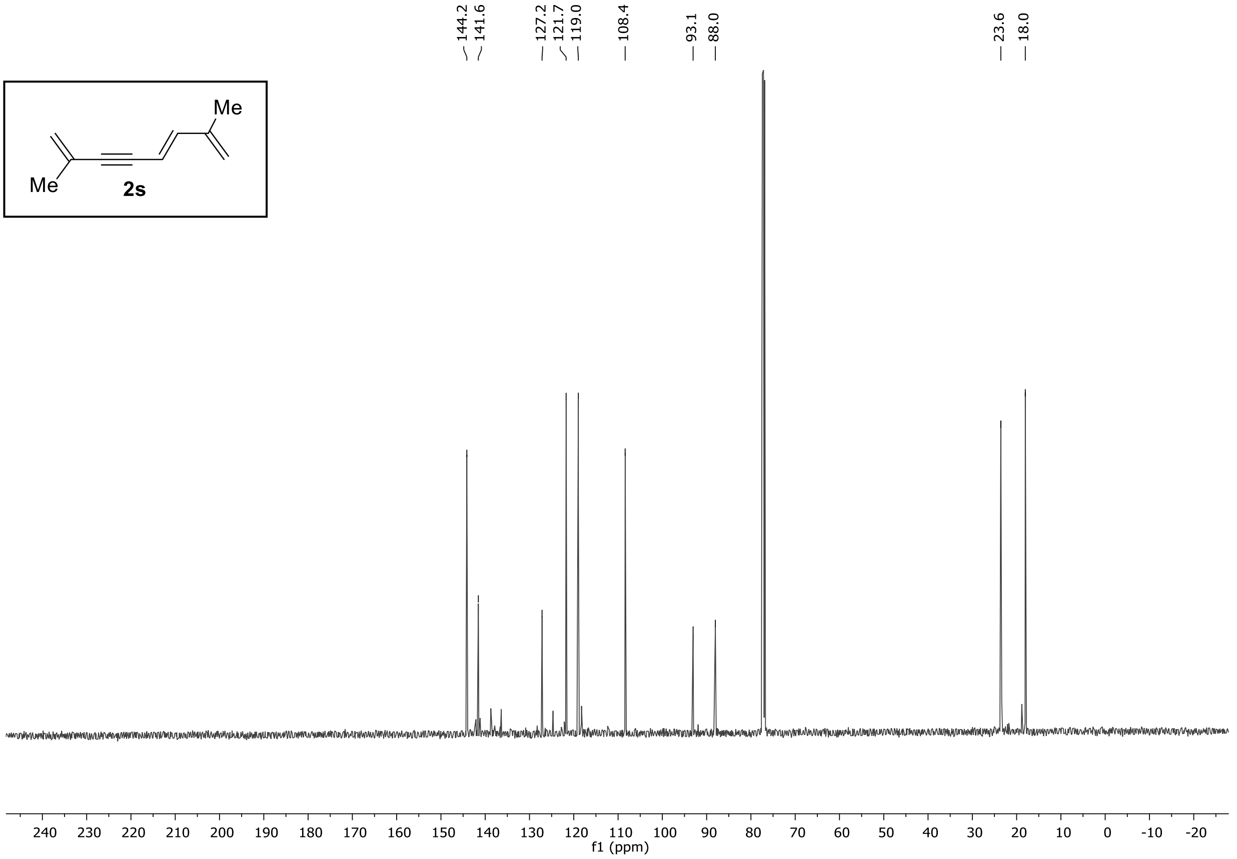
144.2
141.6
127.2
121.7
119.0
108.4
93.1
88.0
23.6
18.0
Me
Me
2s
f1 (ppm)

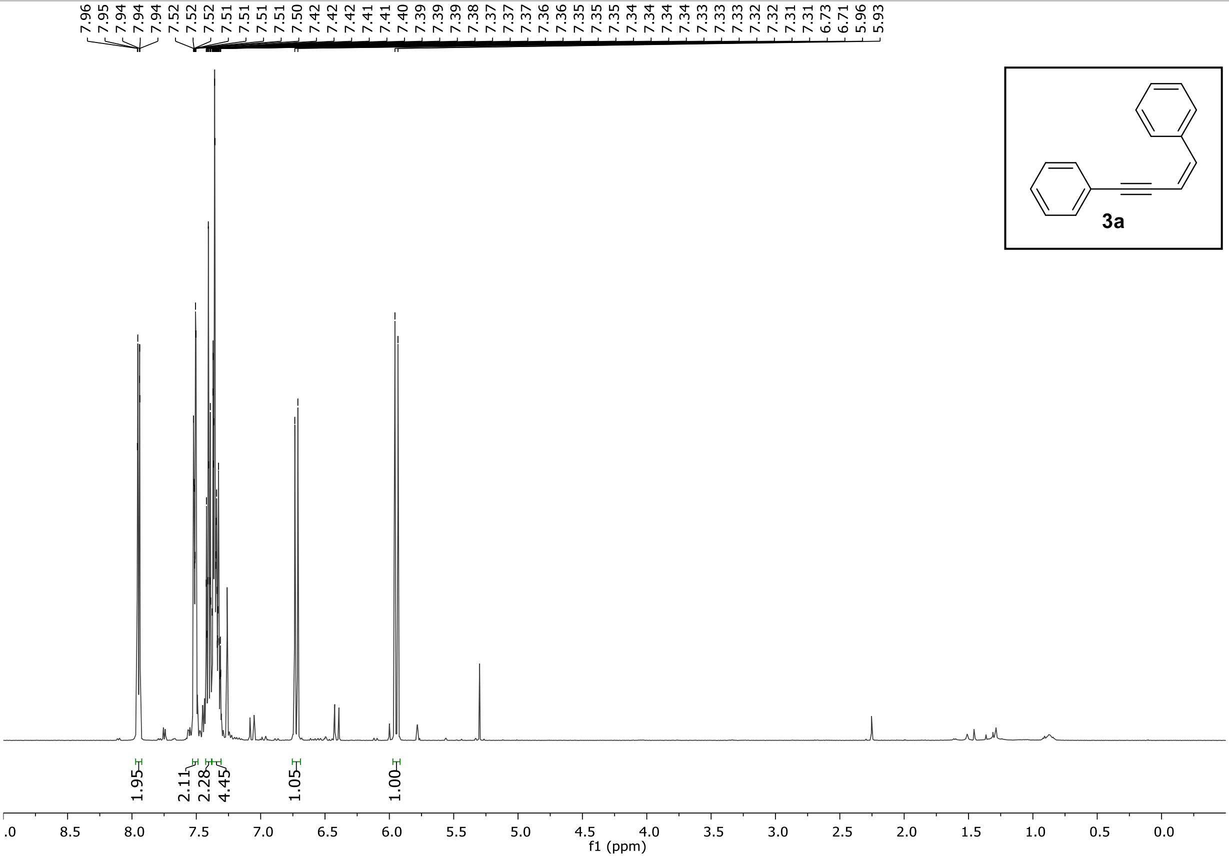
7.96
7.95
7.94
7.94
7.94
7.52
7.52
7.52
7.51
7.51
7.51
7.51
7.50
7.42
7.42
7.42
7.41
7.41
7.40
7.39
7.39
7.39
7.38
7.37
7.37
7.37
7.36
7.36
7.35
7.35
7.35
7.34
7.34
7.34
7.34
7.33
7.33
7.33
7.32
7.32
7.31
7.31
6.73
6.71
5.96
5.93
3a
1.95
2.11
2.28
4.45
1.05
1.00
f1 (ppm)

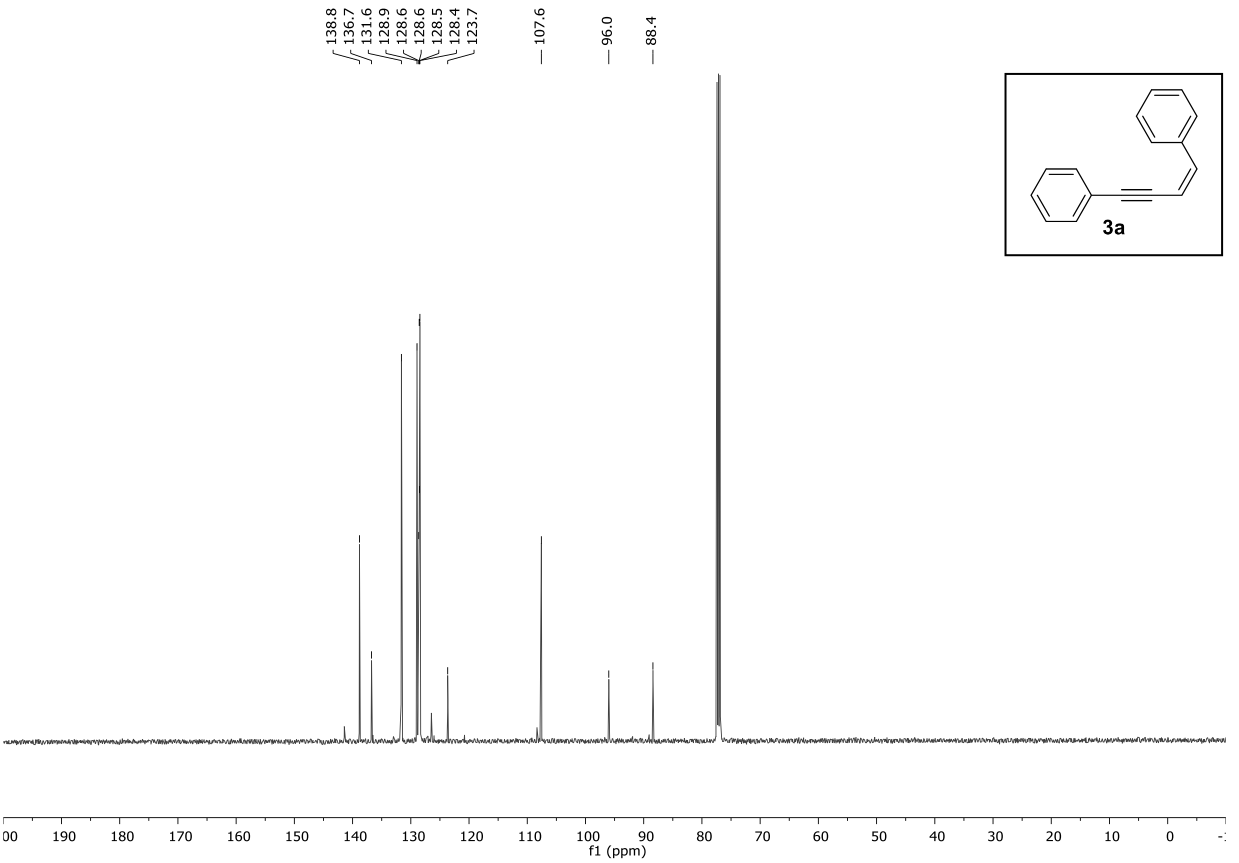
138.8
136.7
131.6
128.9
128.6
128.6
128.5
128.4
123.7
107.6
96.0
88.4
3a
f1 (ppm)

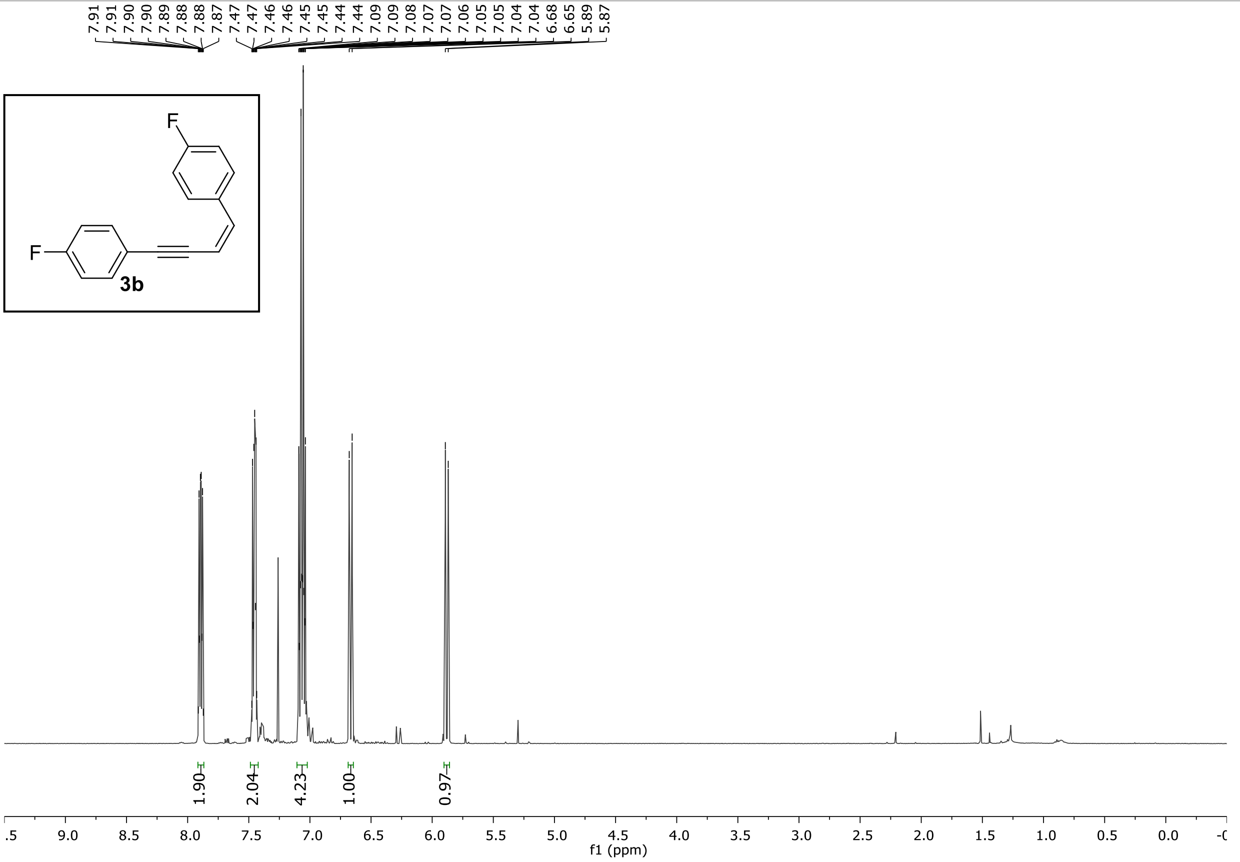
7.91
7.91
7.90
7.90
7.89
7.88
7.88
7.87
7.47
7.47
7.46
7.46
7.45
7.45
7.44
7.44
7.09
7.09
7.08
7.07
7.07
7.06
7.05
7.05
7.04
7.04
6.68
6.65
5.89
5.87
F
F
3b
1.90
2.04
4.23
1.00
0.97
f1 (ppm)

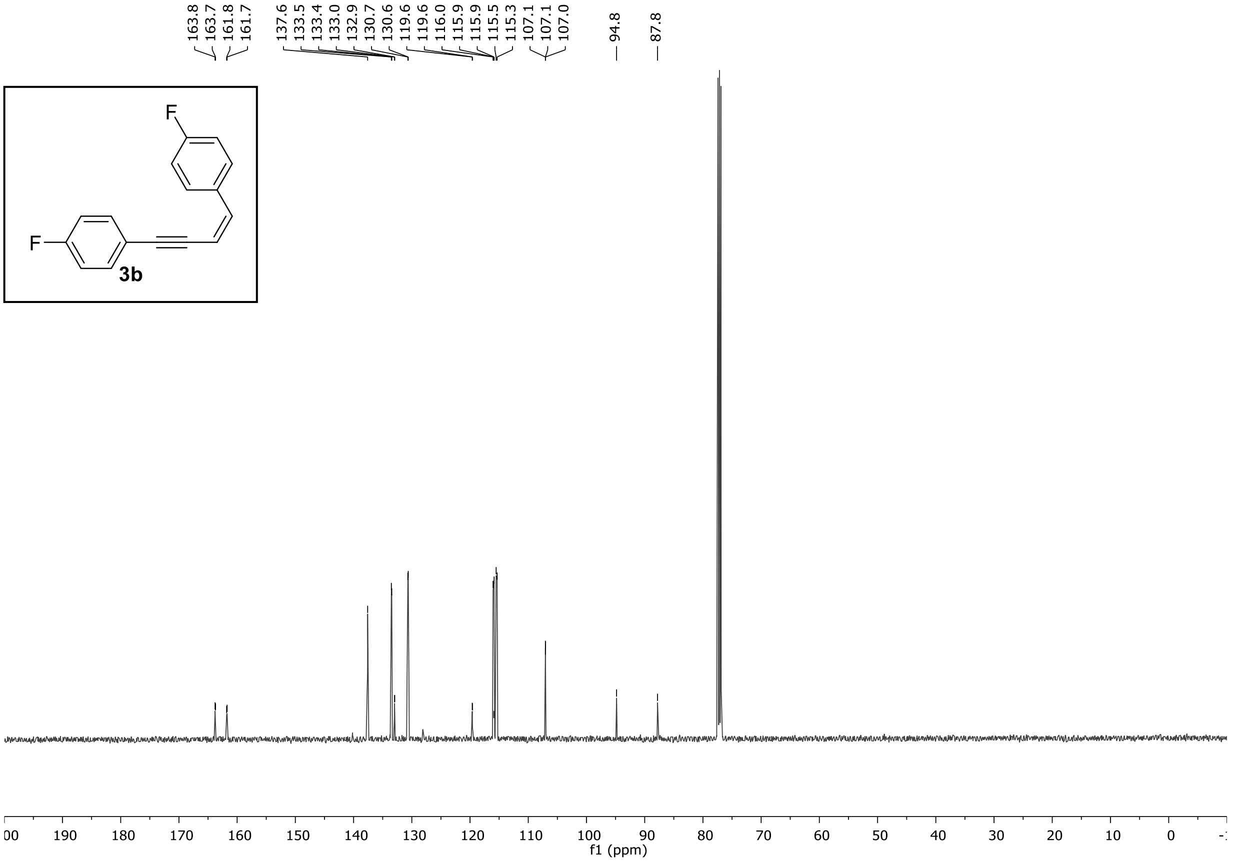
163.8
163.7
161.8
161.7
137.6
133.5
133.4
133.0
132.9
130.7
130.6
119.6
119.6
116.0
115.9
115.9
115.5
115.3
107.1
107.1
107.0
94.8
87.8
F
F
3b
f1 (ppm)

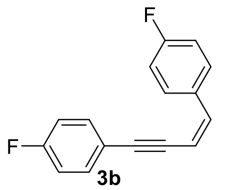

3b

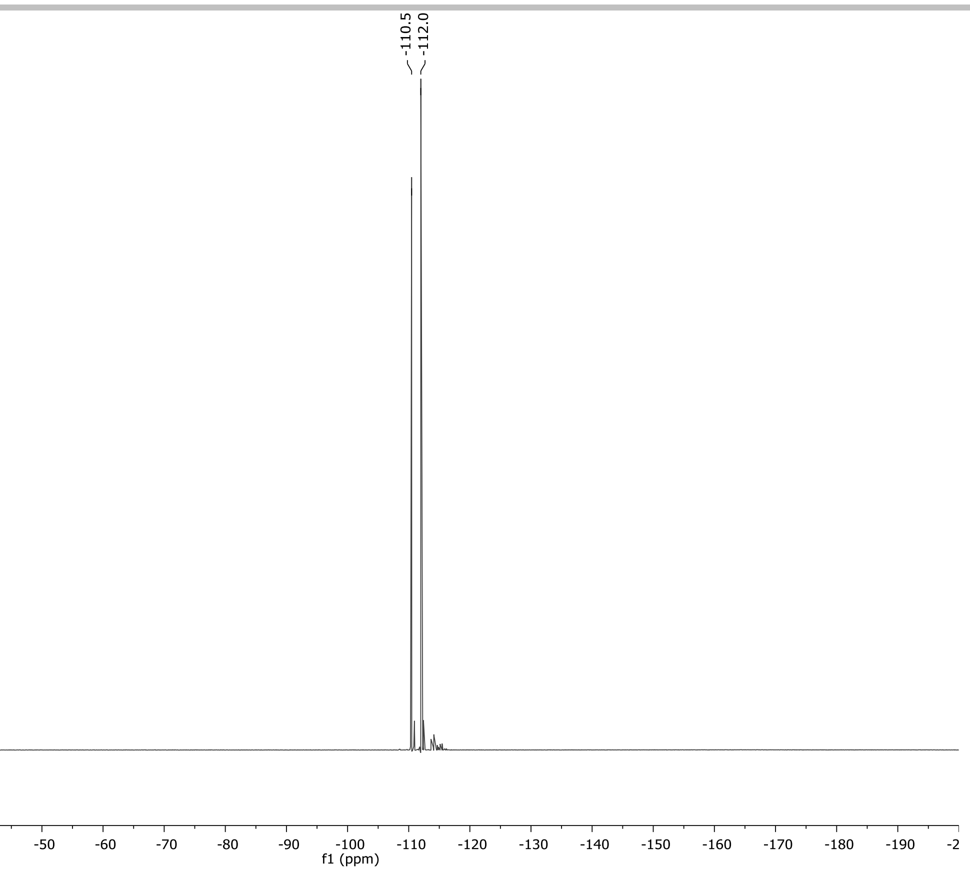
-110.5
-112.0
0
-10
-20
-30
-40
-50
-60
-70
-80
-90
-100
-110
-120
-130
-140
-150
-160
-170
-180
-190
-2
f1 (ppm)

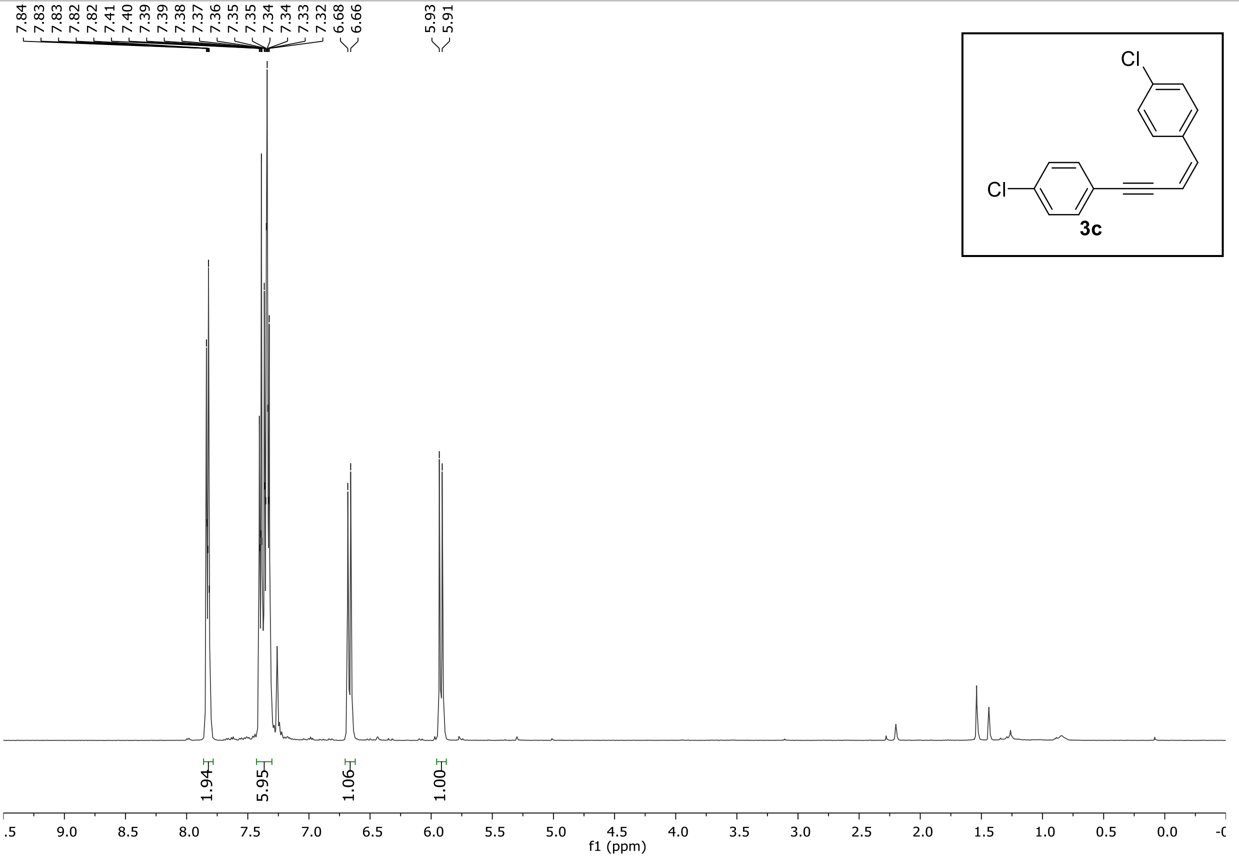
7.84
7.83
7.83
7.82
7.82
7.41
7.40
7.39
7.39
7.38
7.37
7.36
7.35
7.35
7.34
7.34
7.33
7.32
6.68
6.66
5.93
5.91
Cl
Cl
3c
1.94
5.95
1.06
1.00
9.0
8.5
8.0
7.5
7.0
6.5
6.0
5.5
5.0
4.5
4.0
3.5
3.0
2.5
2.0
1.5
1.0
0.5
0.0
f1 (ppm)

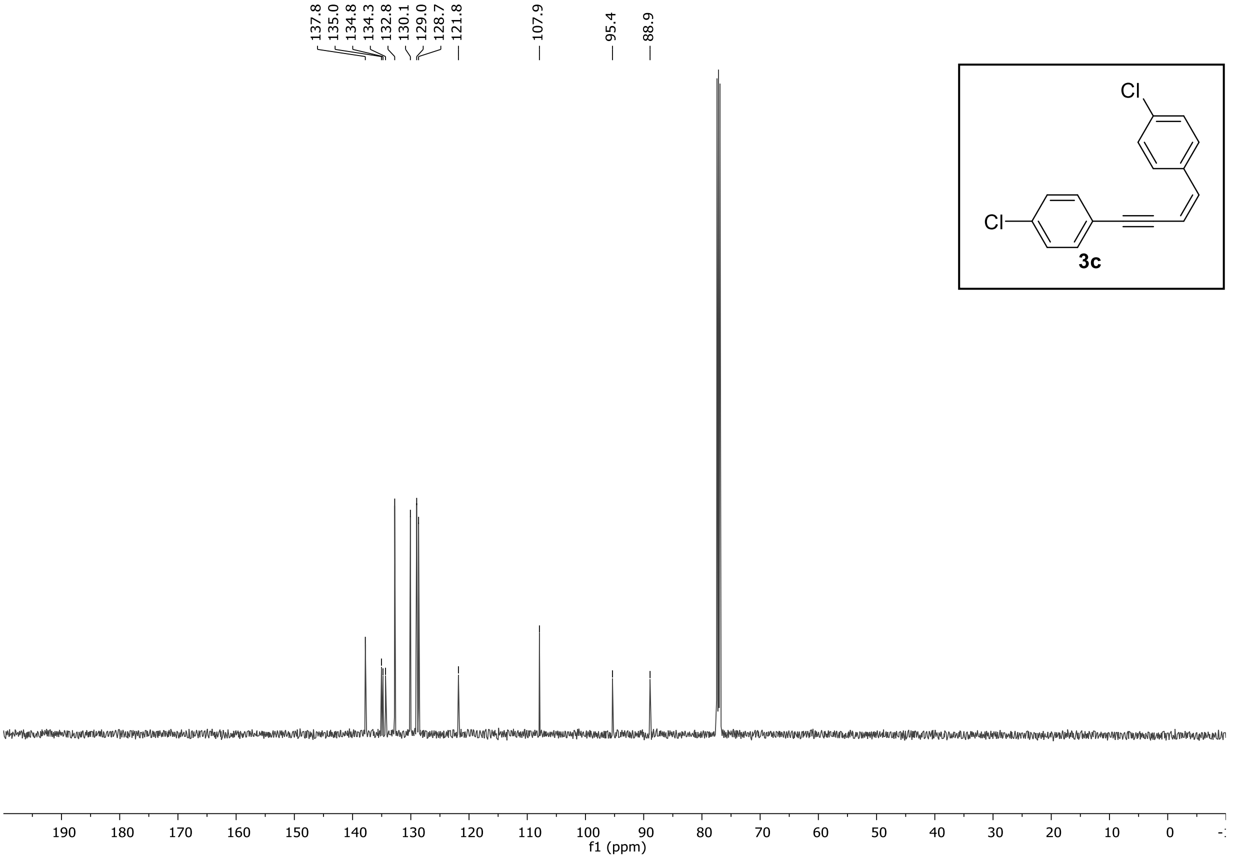
137.8
135.0
134.8
134.3
132.8
130.1
129.0
128.7
121.8
107.9
95.4
88.9
Cl
Cl
3c
190
180
170
160
150
140
130
120
110
100
90
80
70
60
50
40
30
20
10
0
f1 (ppm)

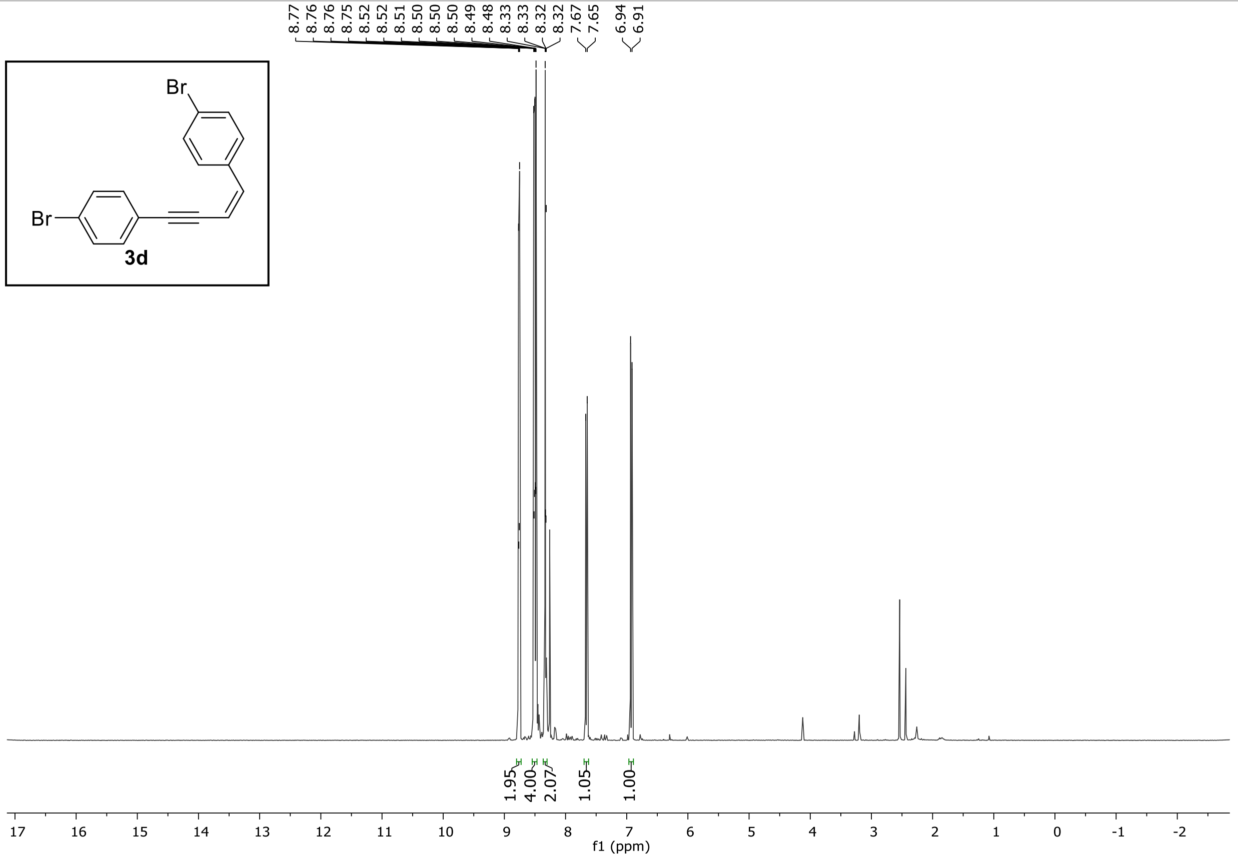
8.77
8.76
8.76
8.75
8.52
8.52
8.51
8.50
8.50
8.50
8.49
8.48
8.33
8.33
8.32
8.32
7.67
7.65
6.94
6.91
Br
Br
3d
1.95
4.00
2.07
1.05
1.00
17 16 15 14 13 12 11 10 9 8 7 6 5 4 3 2 1 0 -1 -2
f1 (ppm)

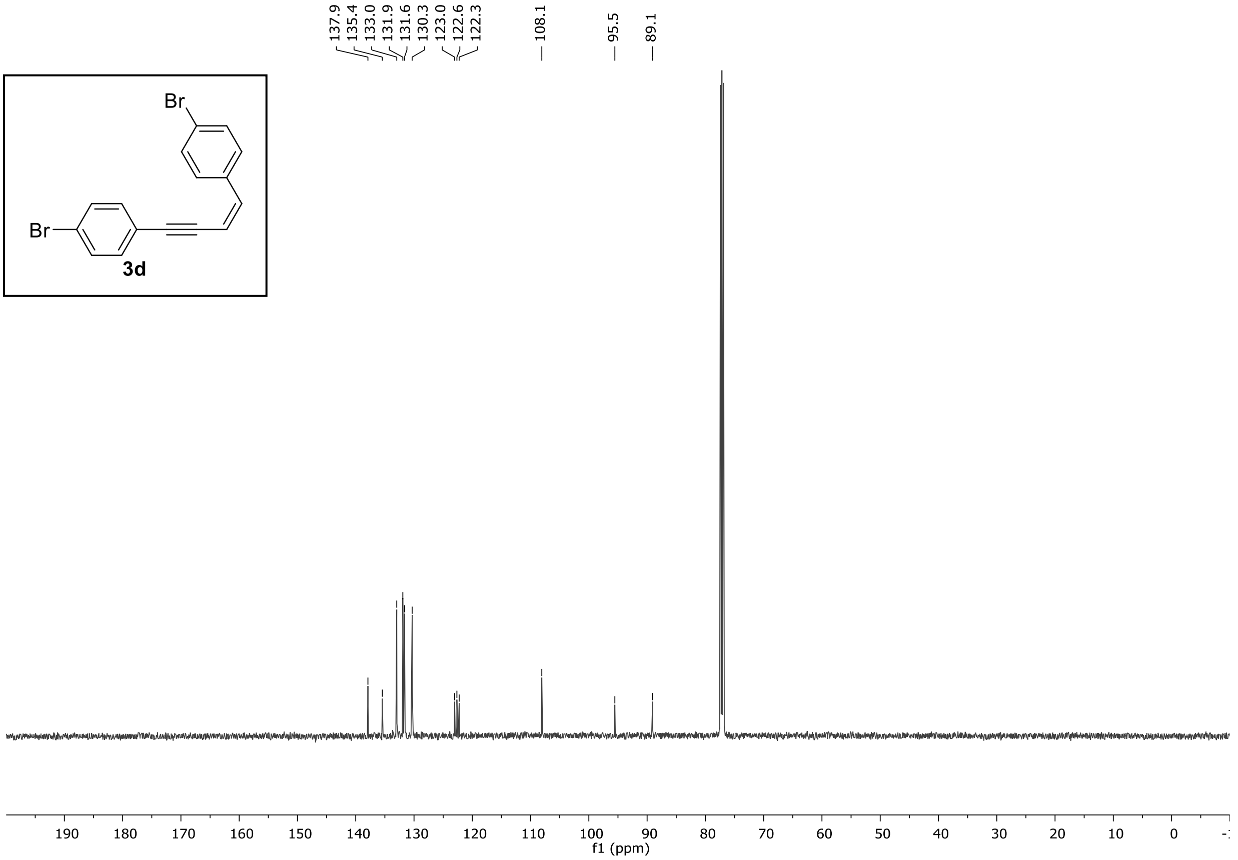
137.9
135.4
133.0
131.9
131.6
130.3
123.0
122.6
122.3
108.1
95.5
89.1
Br
Br
3d
190 180 170 160 150 140 130 120 110 100 90 80 70 60 50 40 30 20 10 0
f1 (ppm)

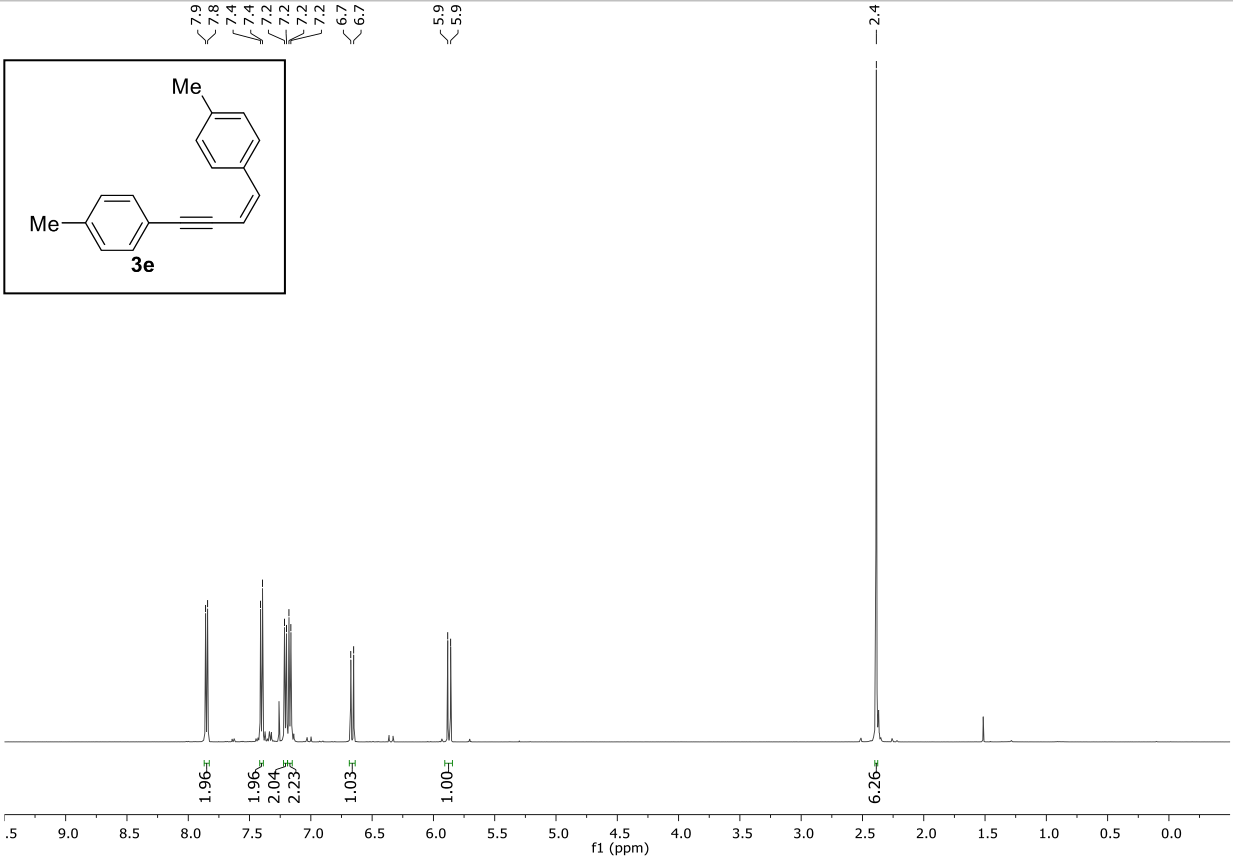
7.9
7.8
7.4
7.4
7.2
7.2
7.2
7.2
6.7
6.7
5.9
5.9
2.4
Me
Me
3e
1.96
1.96
2.04
2.23
1.03
1.00
6.26
f1 (ppm)

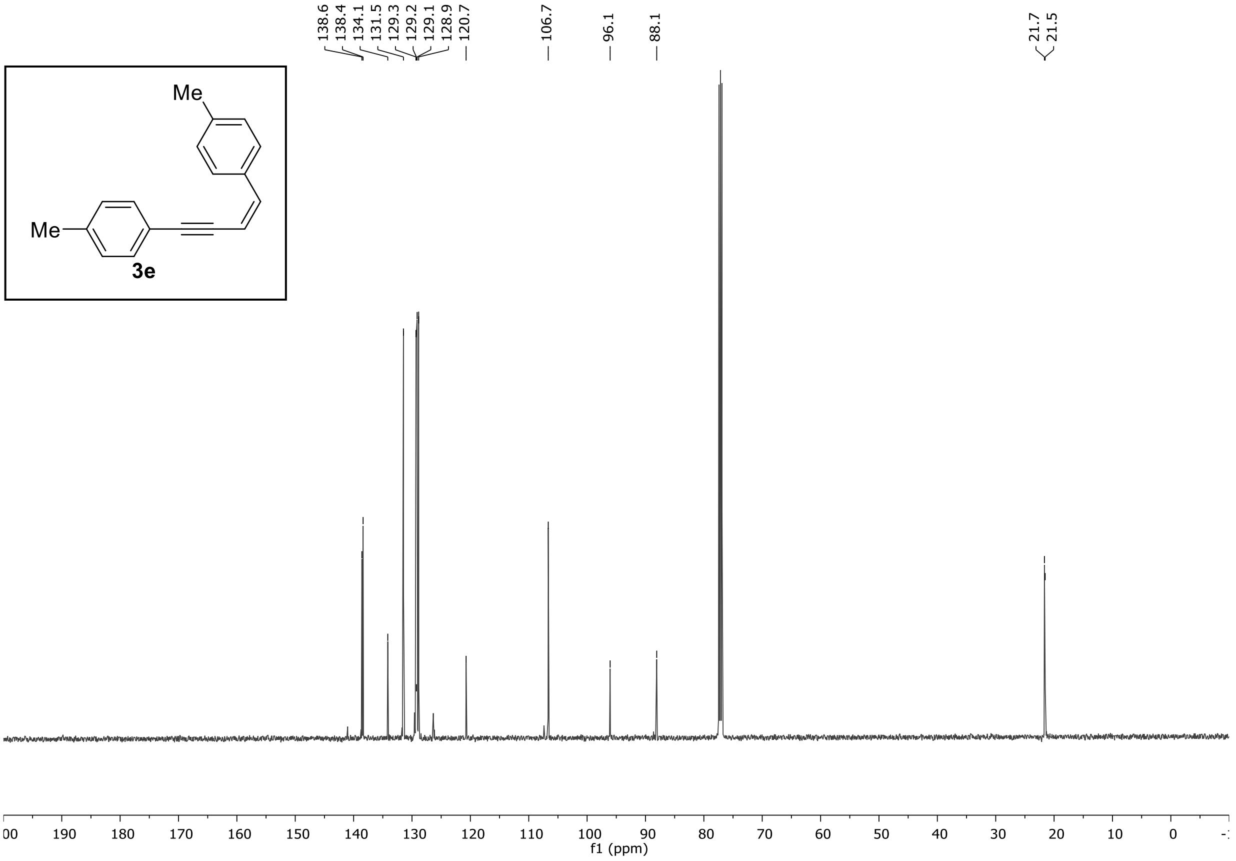
138.6
138.4
134.1
131.5
129.3
129.2
129.1
128.9
120.7
106.7
96.1
88.1
21.7
21.5
Me
Me
3e
f1 (ppm)

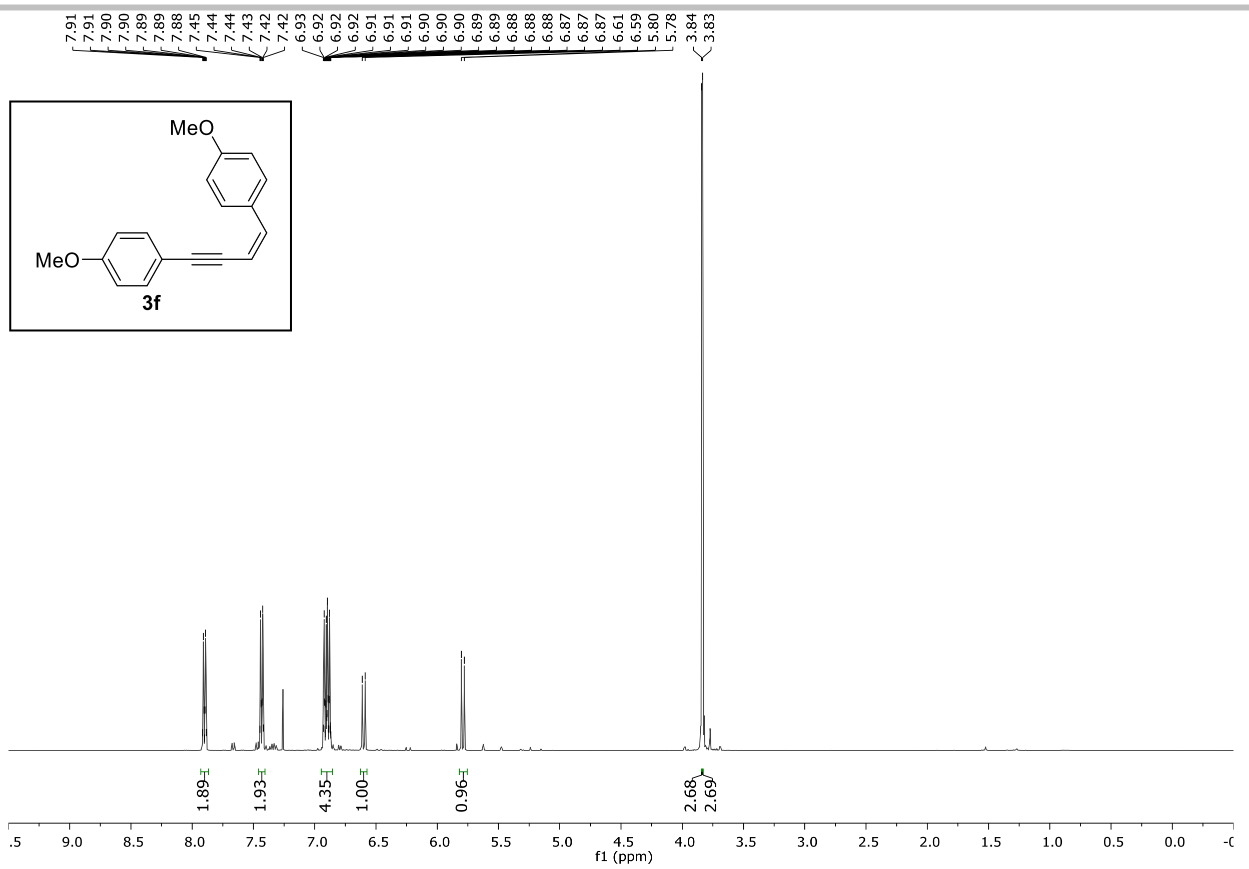
7.91
7.91
7.90
7.90
7.89
7.89
7.88
7.45
7.44
7.44
7.43
7.42
7.42
6.93
6.92
6.92
6.92
6.91
6.91
6.91
6.90
6.90
6.90
6.89
6.89
6.88
6.88
6.88
6.87
6.87
6.87
6.61
6.59
5.80
5.78
3.84
3.83
MeO
MeO
3f
1.89
1.93
4.35
1.00
0.96
2.68
2.69
f1 (ppm)

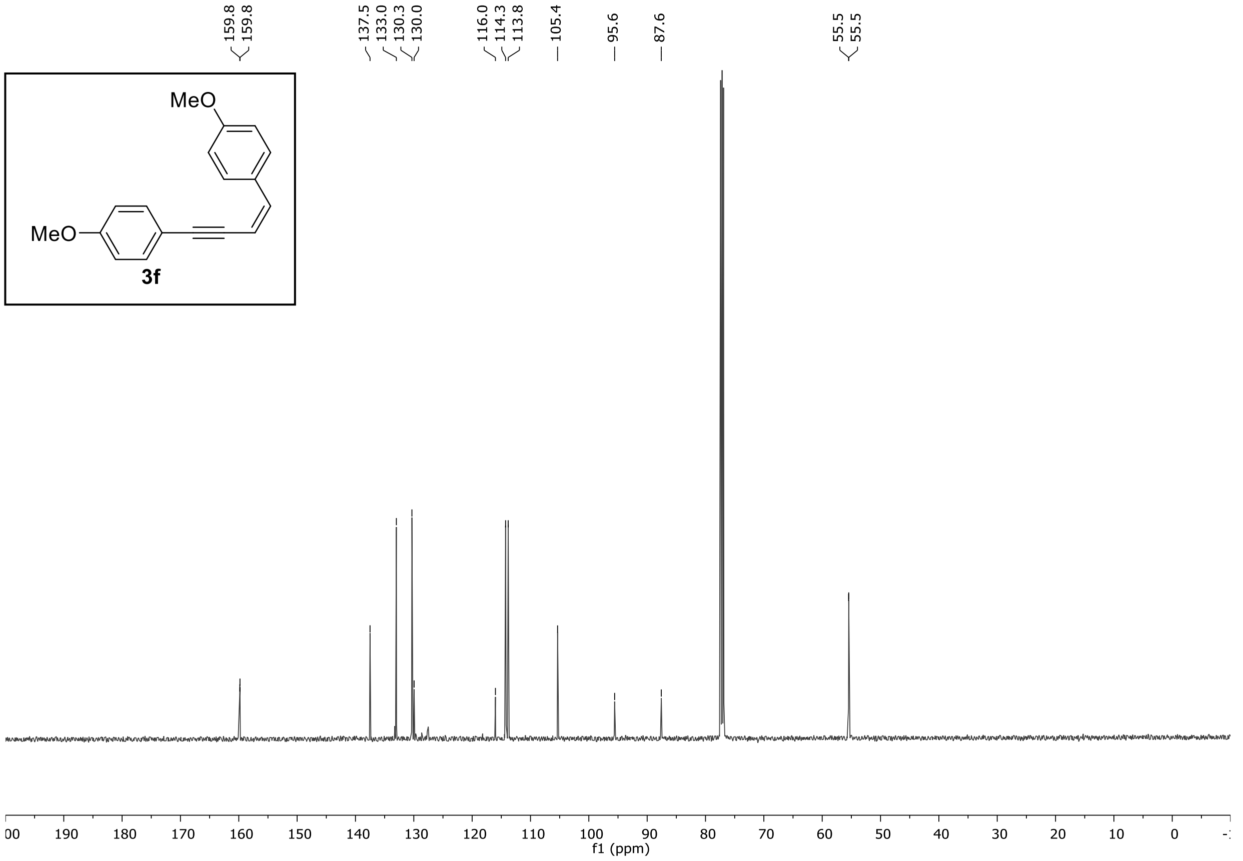
159.8
159.8
137.5
133.0
130.3
130.0
116.0
114.3
113.8
105.4
95.6
87.6
55.5
55.5
MeO
MeO
3f
f1 (ppm)

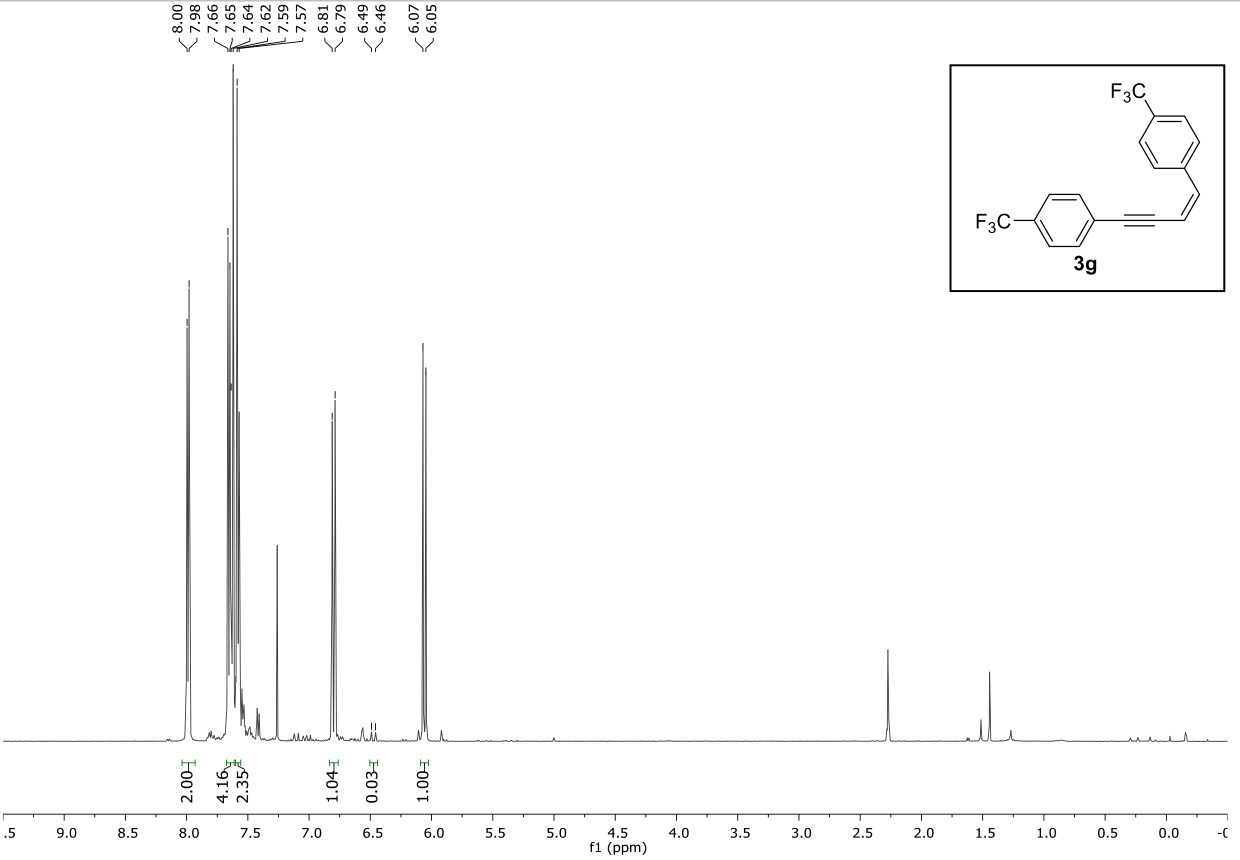
8.00
7.98
7.66
7.65
7.64
7.62
7.59
7.57
6.81
6.79
6.49
6.46
6.07
6.05
2.00
4.16
2.35
1.04
0.03
1.00
F3C
F3C
3g
f1 (ppm)

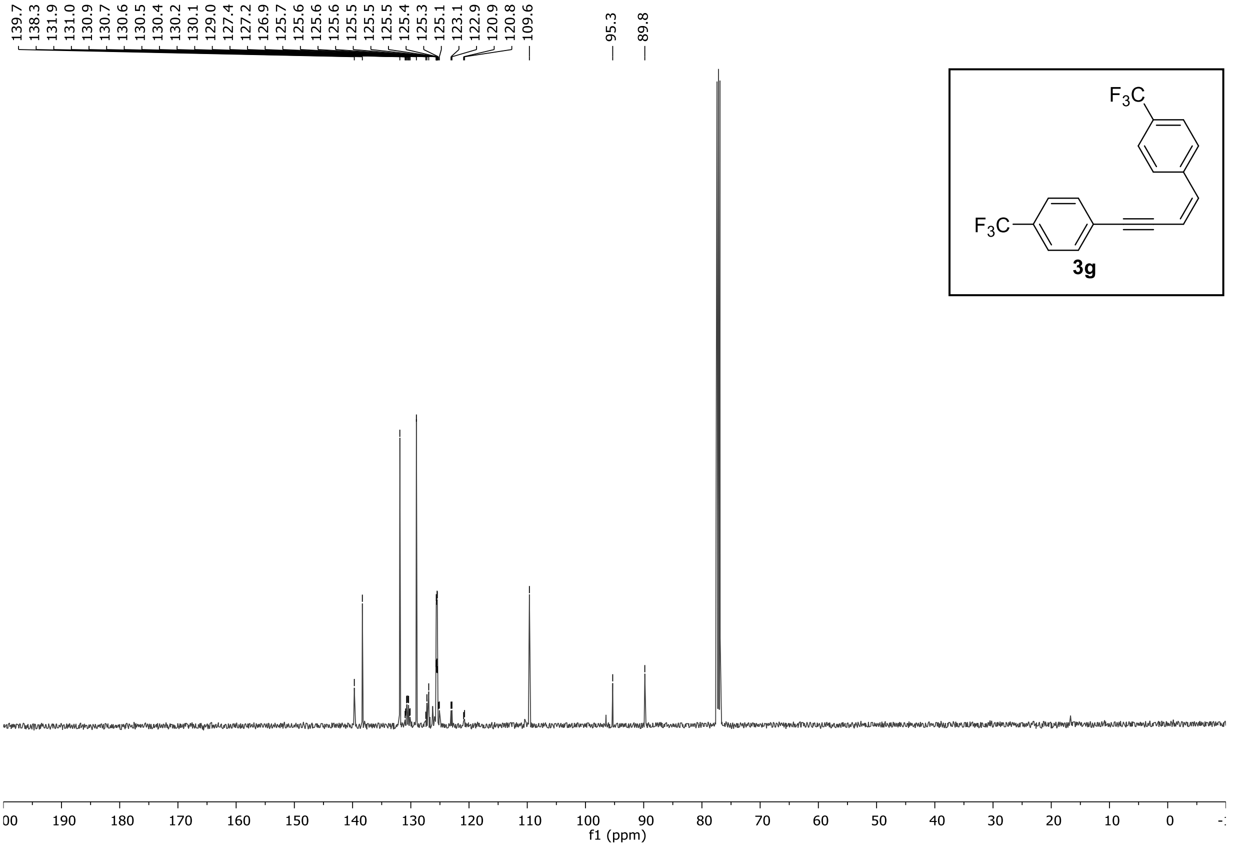
139.7
138.3
131.9
131.0
130.9
130.7
130.6
130.5
130.4
130.2
130.1
129.0
127.4
127.2
126.9
125.7
125.6
125.6
125.6
125.5
125.5
125.5
125.4
125.3
125.1
123.1
122.9
120.9
120.8
109.6
95.3
89.8
F3C
F3C
3g
f1 (ppm)

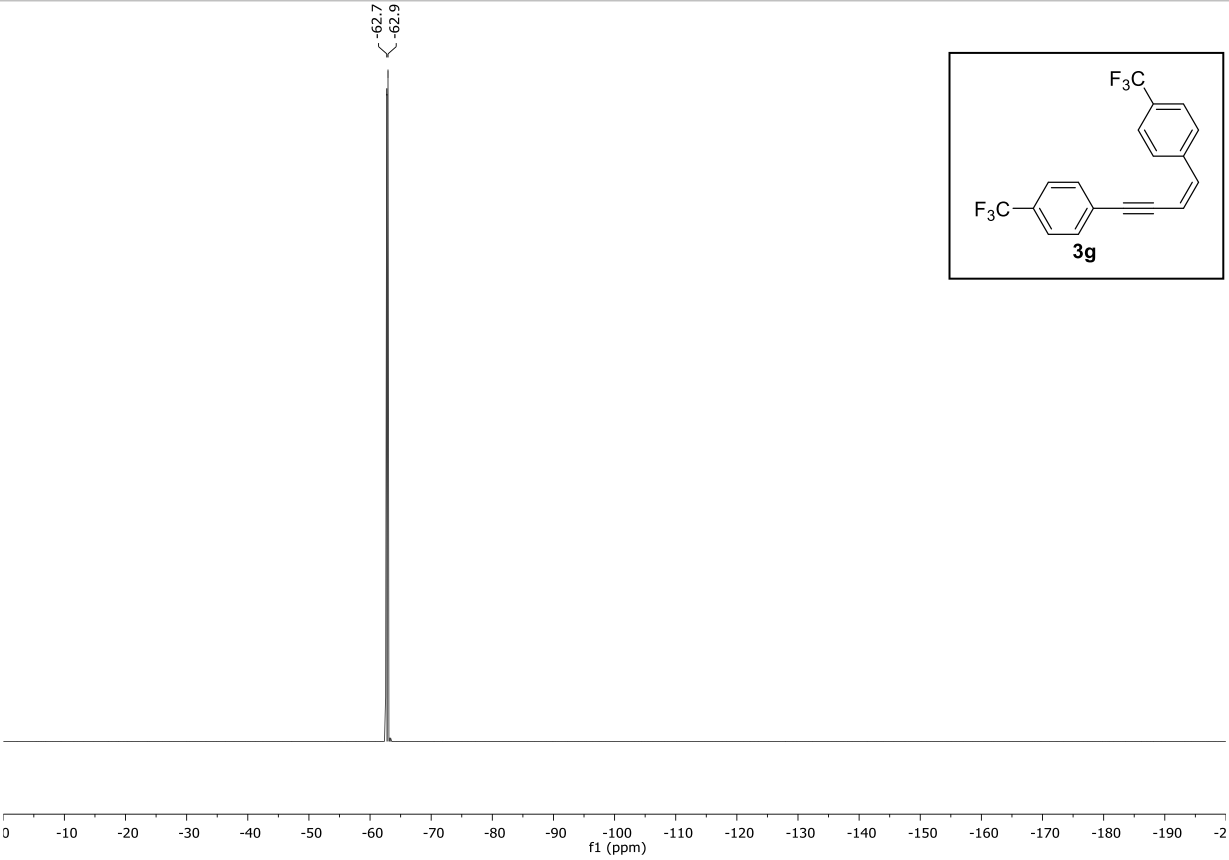
-62.7
-62.9
F3C
F3C
3g
0 -10 -20 -30 -40 -50 -60 -70 -80 -90 -100 -110 -120 -130 -140 -150 -160 -170 -180 -190 -2
f1 (ppm)

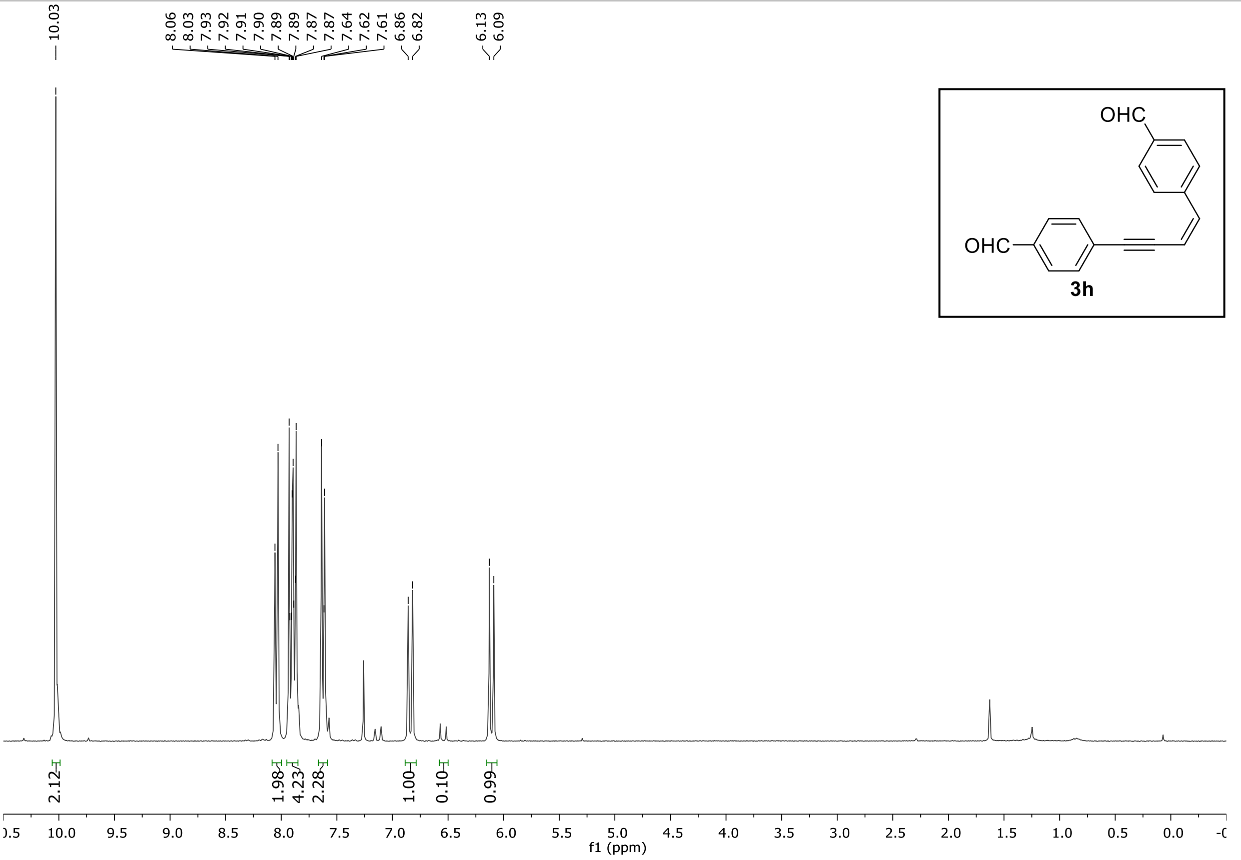
10.03
8.06
8.03
7.93
7.92
7.91
7.90
7.89
7.89
7.87
7.87
7.64
7.62
7.61
6.86
6.82
6.13
6.09
OHC
OHC
3h
2.12
1.98
4.23
2.28
1.00
0.10
0.99
f1 (ppm)

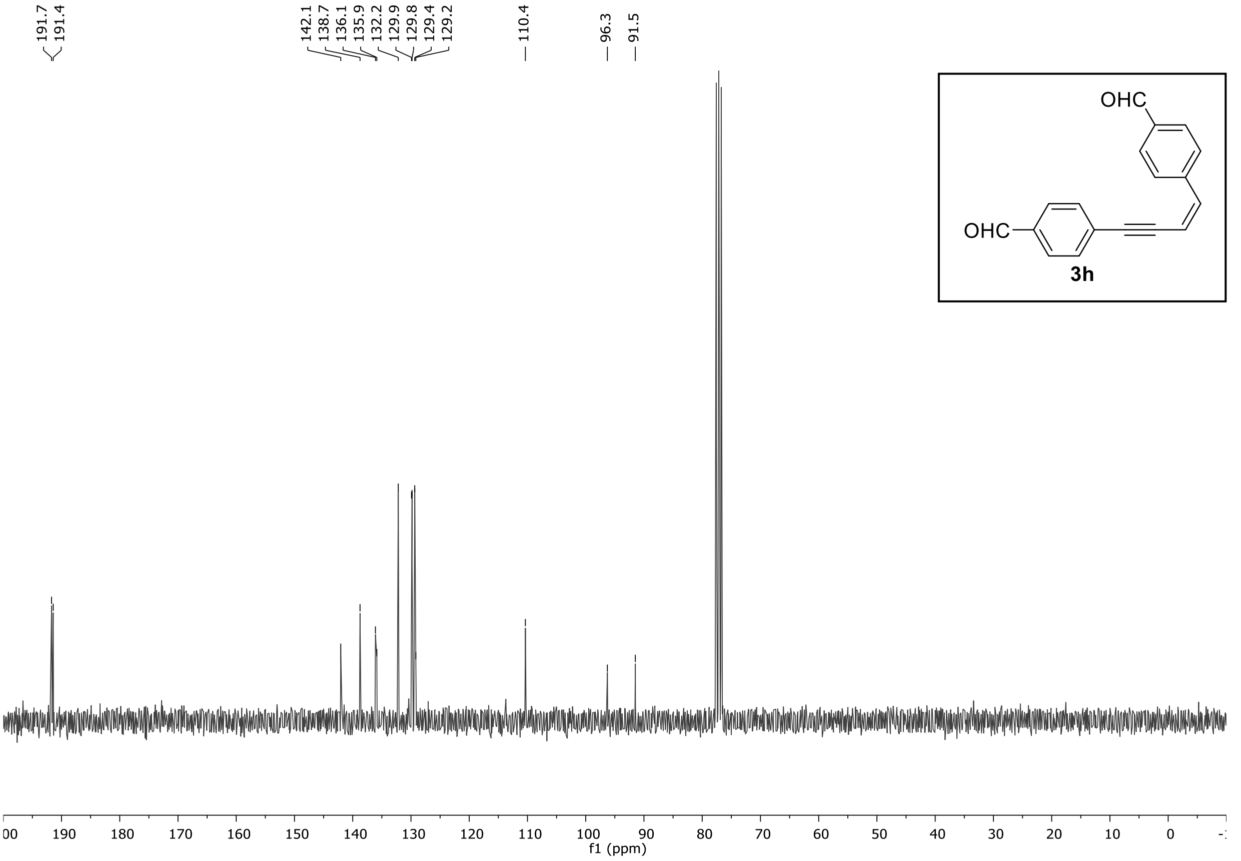
191.7
191.4
142.1
138.7
136.1
135.9
132.2
129.9
129.8
129.4
129.2
110.4
96.3
91.5
OHC
OHC
3h
f1 (ppm)

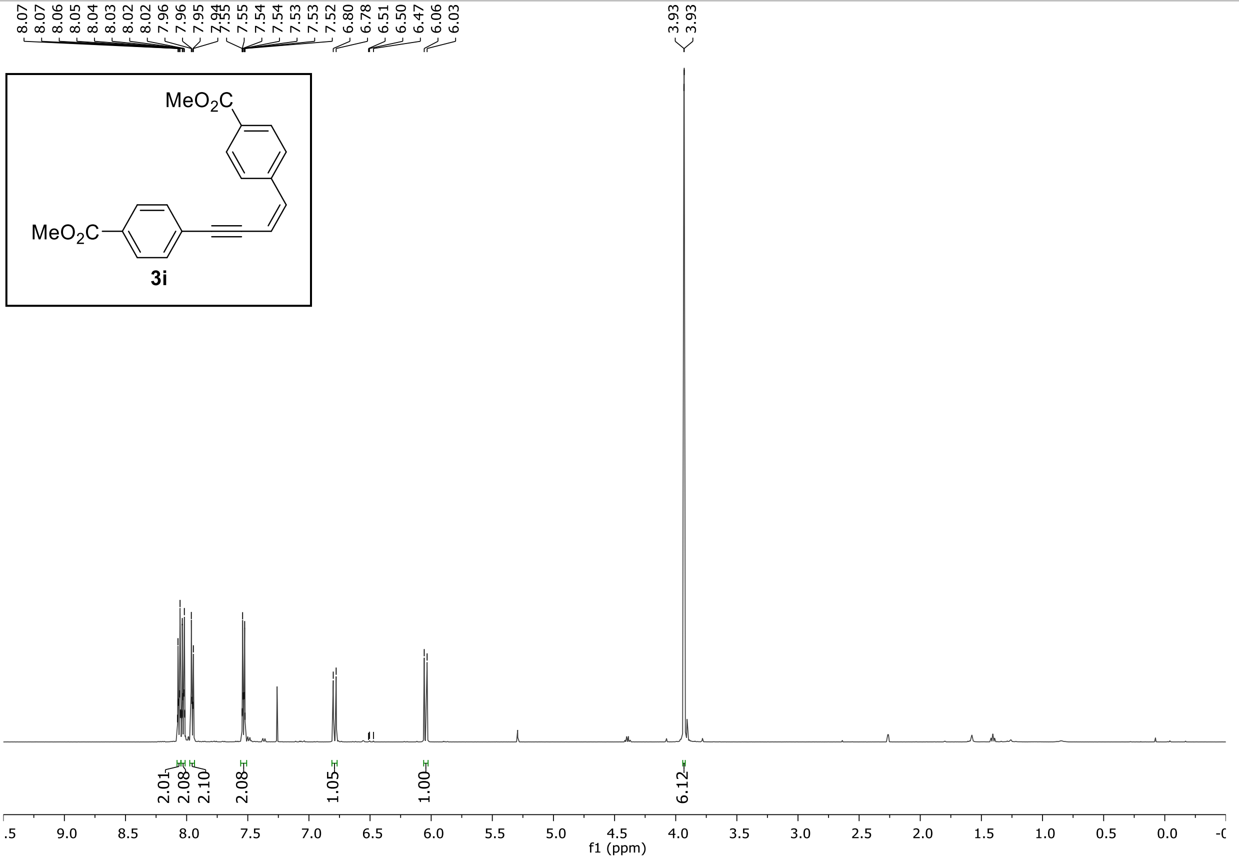
8.07
8.07
8.06
8.05
8.04
8.03
8.02
8.02
7.96
7.96
7.95
7.94
7.55
7.55
7.54
7.54
7.53
7.53
7.52
6.80
6.78
6.51
6.50
6.47
6.06
6.03
3.93
3.93
MeO2C
MeO2C
3i
2.01
2.08
2.10
2.08
1.05
1.00
6.12
f1 (ppm)

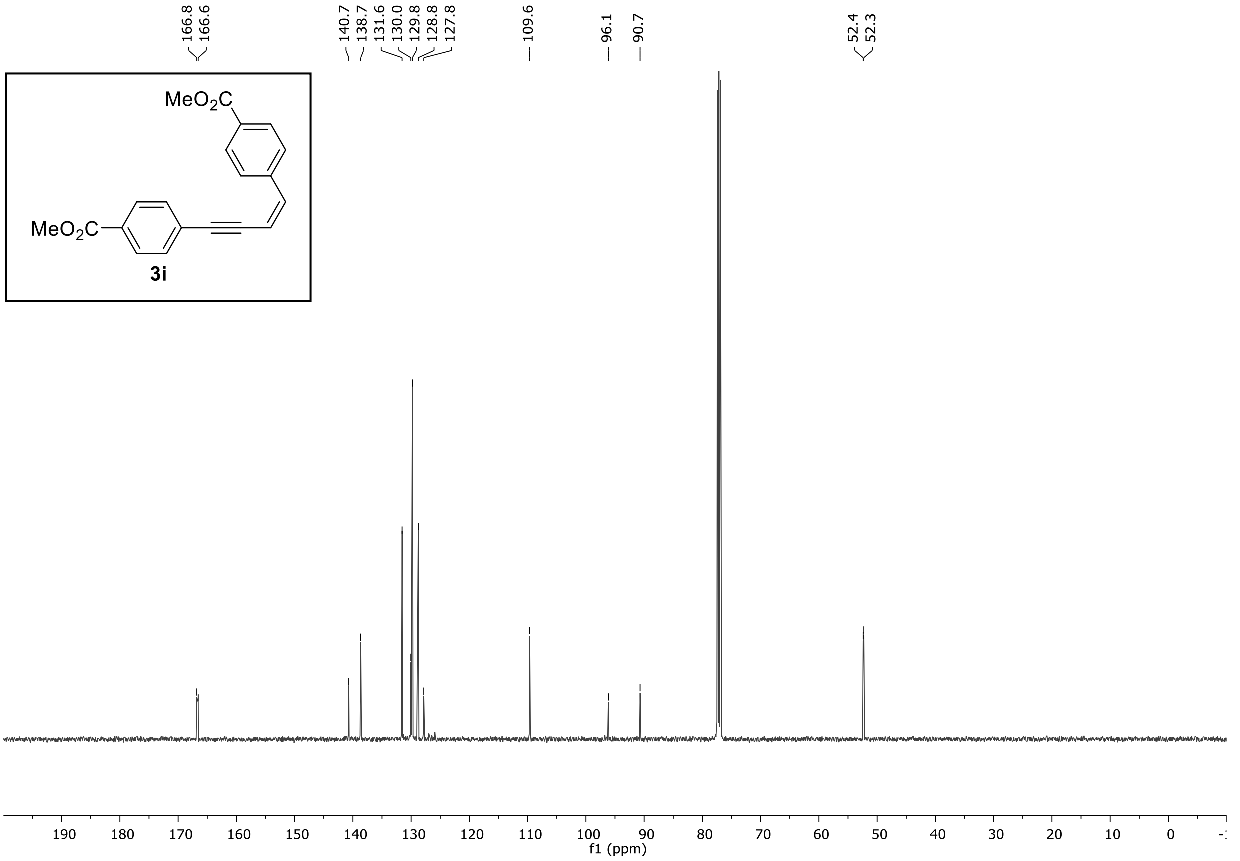
166.8
166.6
140.7
138.7
131.6
130.0
129.8
128.8
127.8
109.6
96.1
90.7
52.4
52.3
MeO2C
MeO2C
3i
f1 (ppm)

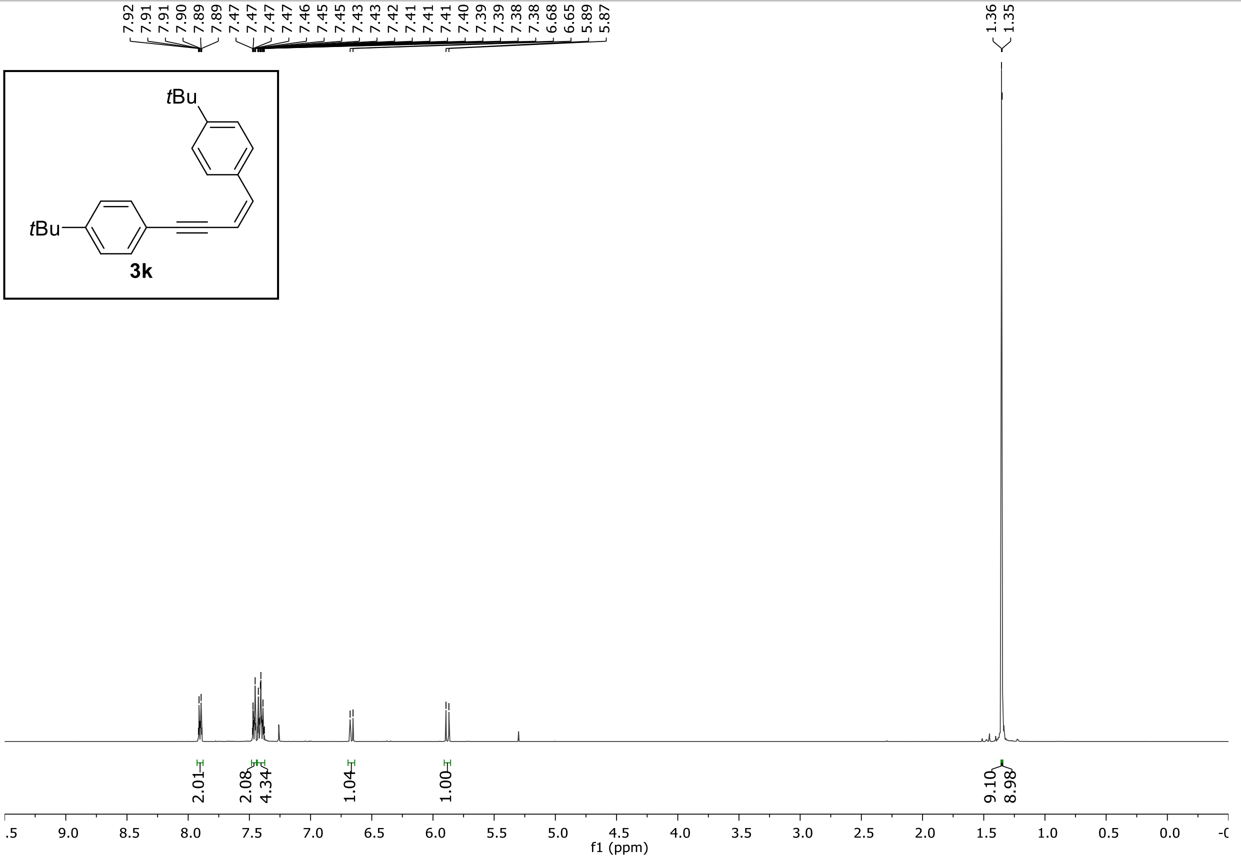
7.92
7.91
7.91
7.90
7.89
7.89
7.47
7.47
7.47
7.47
7.46
7.45
7.45
7.43
7.43
7.42
7.41
7.41
7.41
7.40
7.39
7.39
7.38
7.38
6.68
6.65
5.89
5.87
1.36
1.35
tBu
tBu
3k
2.01
2.08
4.34
1.04
1.00
9.10
8.98
9.0 8.5 8.0 7.5 7.0 6.5 6.0 5.5 5.0 4.5 4.0 3.5 3.0 2.5 2.0 1.5 1.0 0.5 0.0
f1 (ppm)

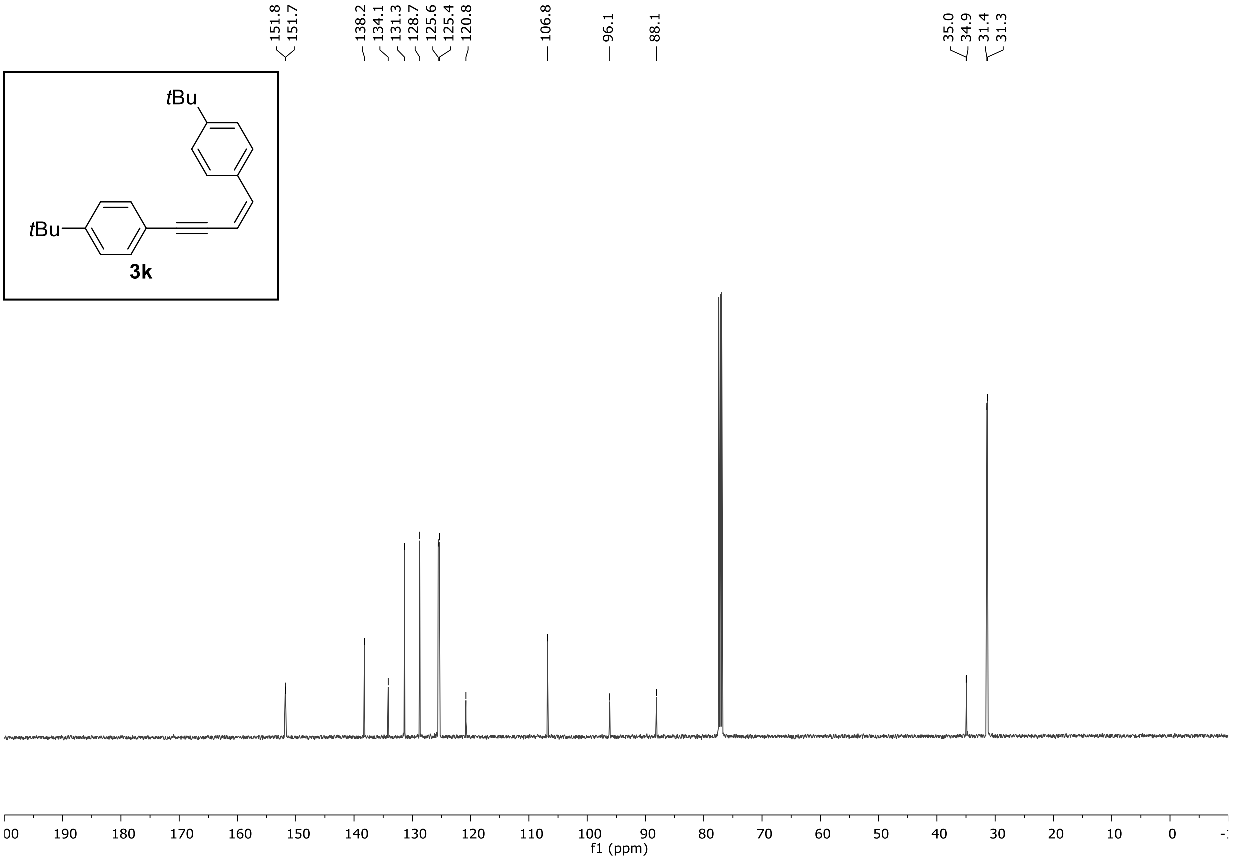
151.8
151.7
138.2
134.1
131.3
128.7
125.6
125.4
120.8
106.8
96.1
88.1
35.0
34.9
31.4
31.3
tBu
tBu
3k
190 180 170 160 150 140 130 120 110 100 90 80 70 60 50 40 30 20 10 0
f1 (ppm)

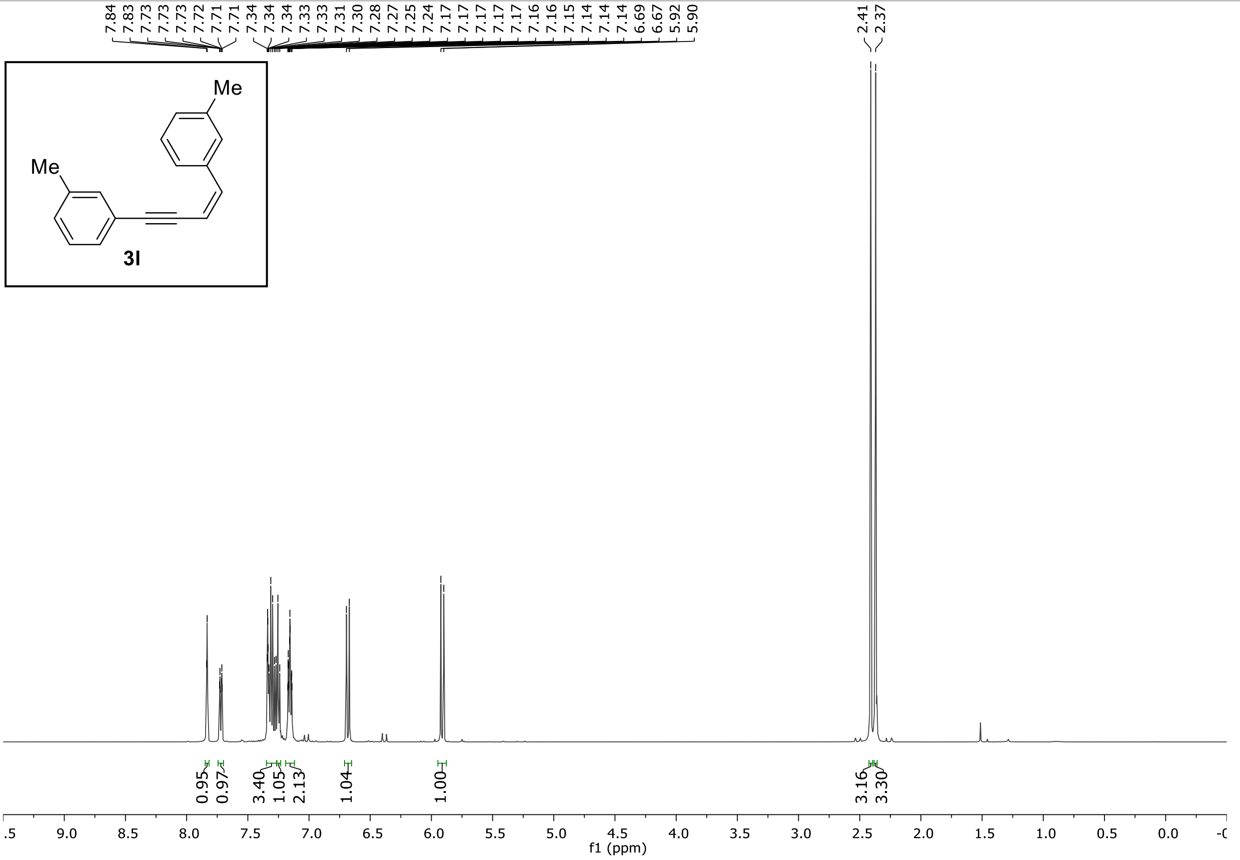
7.84
7.83
7.73
7.73
7.73
7.72
7.71
7.71
7.34
7.34
7.34
7.33
7.33
7.31
7.30
7.28
7.27
7.25
7.24
7.17
7.17
7.17
7.17
7.17
7.16
7.16
7.15
7.14
7.14
7.14
6.69
6.67
5.92
5.90
2.41
2.37
Me
Me
3l
0.95
0.97
3.40
1.05
2.13
1.04
1.00
3.16
3.30
9.0
8.5
8.0
7.5
7.0
6.5
6.0
5.5
5.0
4.5
4.0
3.5
3.0
2.5
2.0
1.5
1.0
0.5
0.0
f1 (ppm)

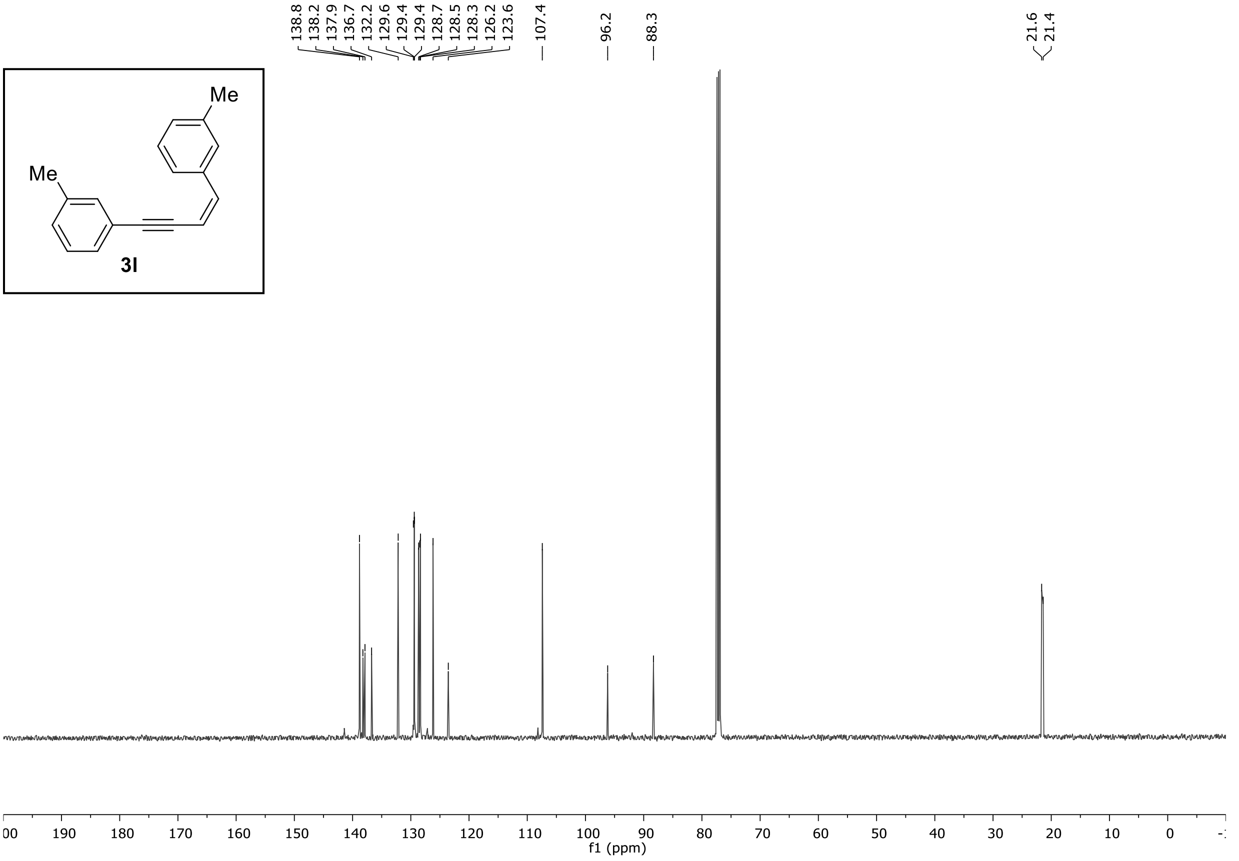
138.8
138.2
137.9
136.7
132.2
129.6
129.4
129.4
128.7
128.5
128.3
126.2
123.6
107.4
96.2
88.3
21.6
21.4
Me
Me
3l
190
180
170
160
150
140
130
120
110
100
90
80
70
60
50
40
30
20
10
0
f1 (ppm)

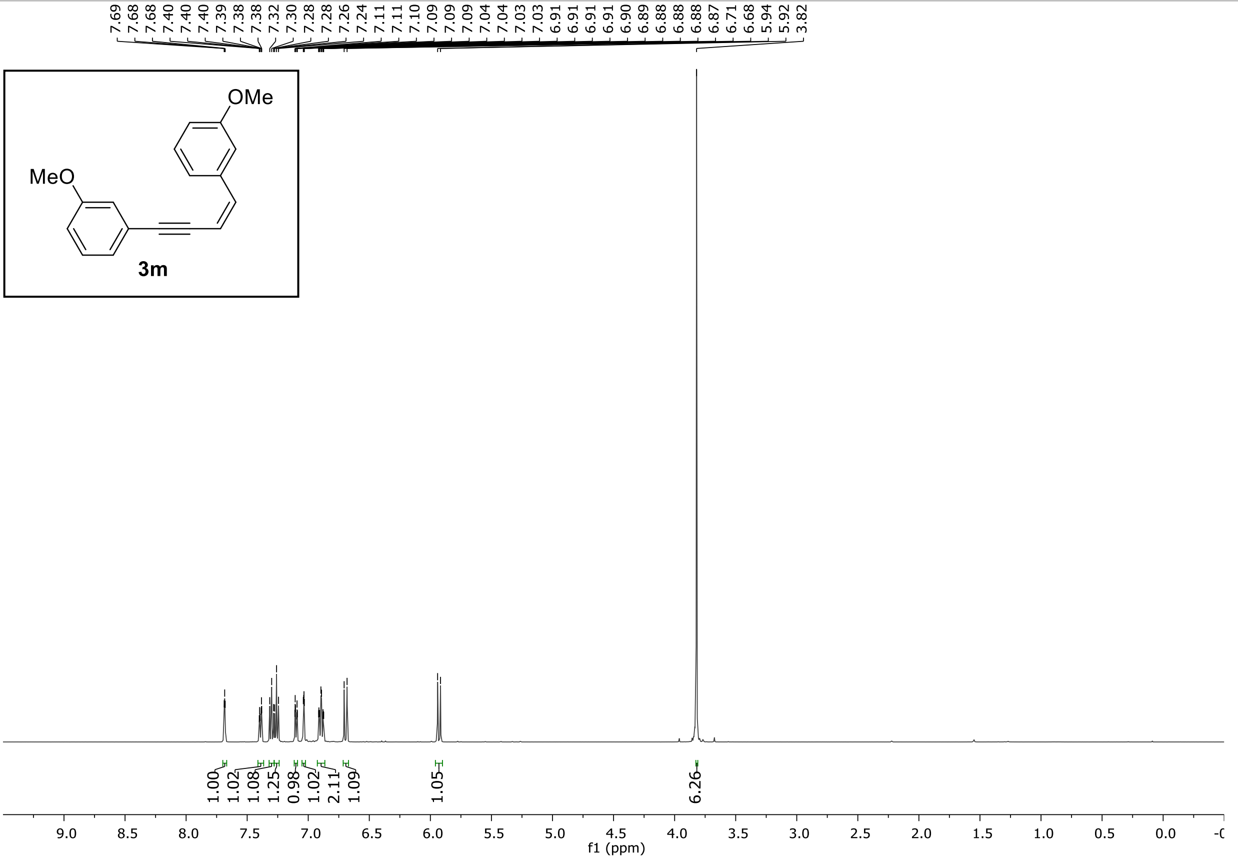
7.69 7.68 7.68 7.40 7.40 7.40 7.39 7.38 7.38 7.32 7.30 7.28 7.28 7.26 7.24 7.11 7.11 7.10 7.09 7.09 7.09 7.04 7.04 7.03 7.03 6.91 6.91 6.91 6.91 6.90 6.89 6.88 6.88 6.88 6.87 6.71 6.68 5.94 5.92 3.82
OMe
MeO
3m
1.00 1.02 1.08 1.25 0.98 1.02 2.11 1.09 1.05 6.26
f1 (ppm)

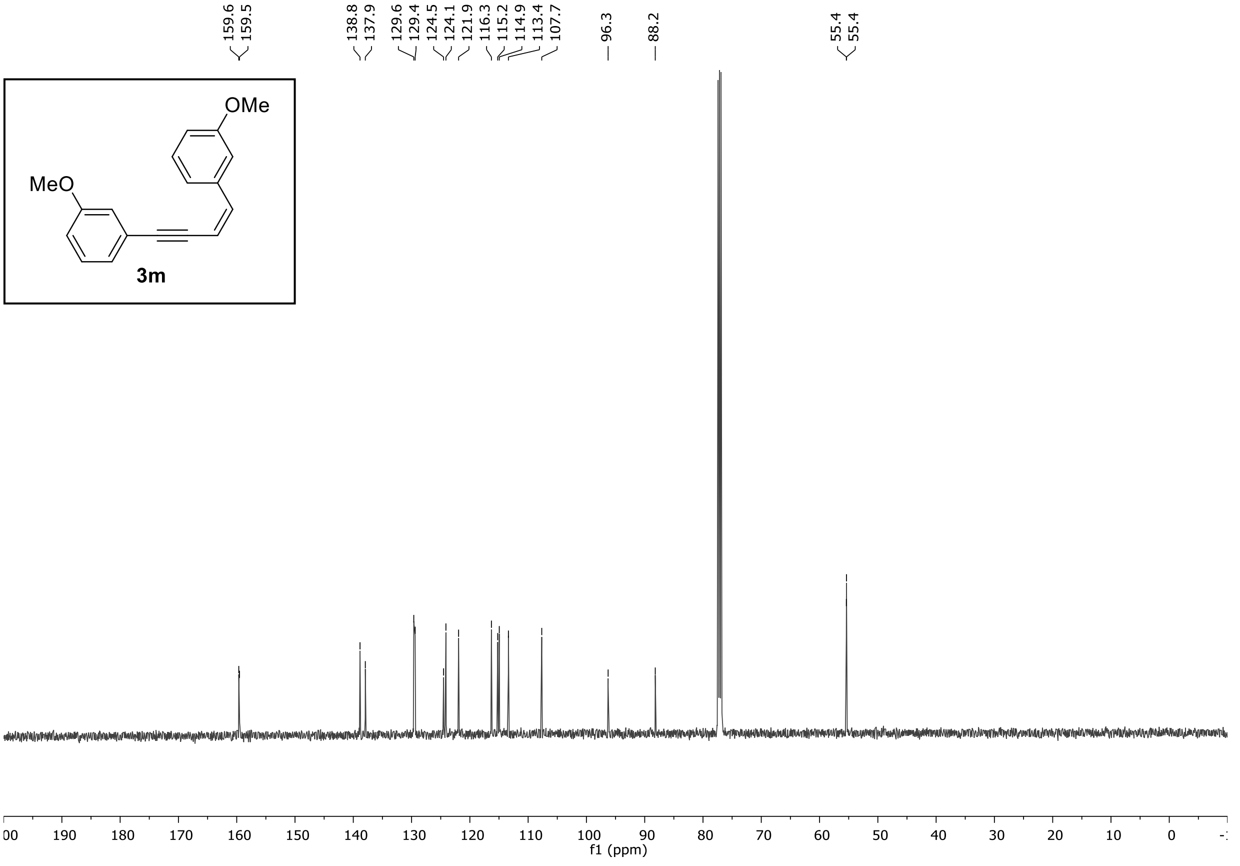
159.6 159.5 138.8 137.9 129.6 129.4 124.5 124.1 121.9 116.3 115.2 114.9 113.4 107.7 96.3 88.2 55.4 55.4
OMe
MeO
3m
f1 (ppm)

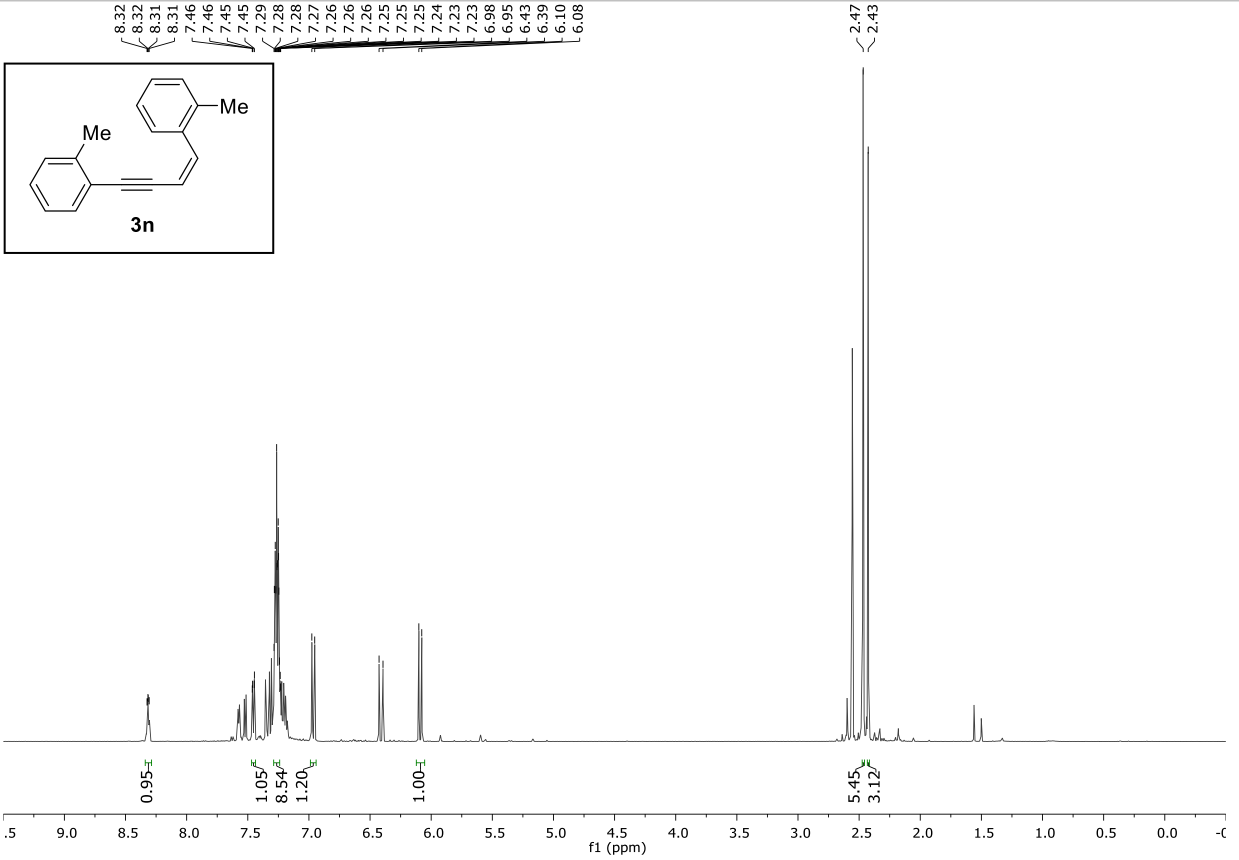
8.32 8.32 8.31 8.31 7.46 7.46 7.45 7.45 7.29 7.28 7.28 7.27 7.26 7.26 7.26 7.25 7.25 7.25 7.24 7.23 7.23 6.98 6.95 6.43 6.39 6.10 6.08
2.47 2.43
Me
Me
3n
0.95
1.05
8.54
1.20
1.00
5.45
3.12
f1 (ppm)

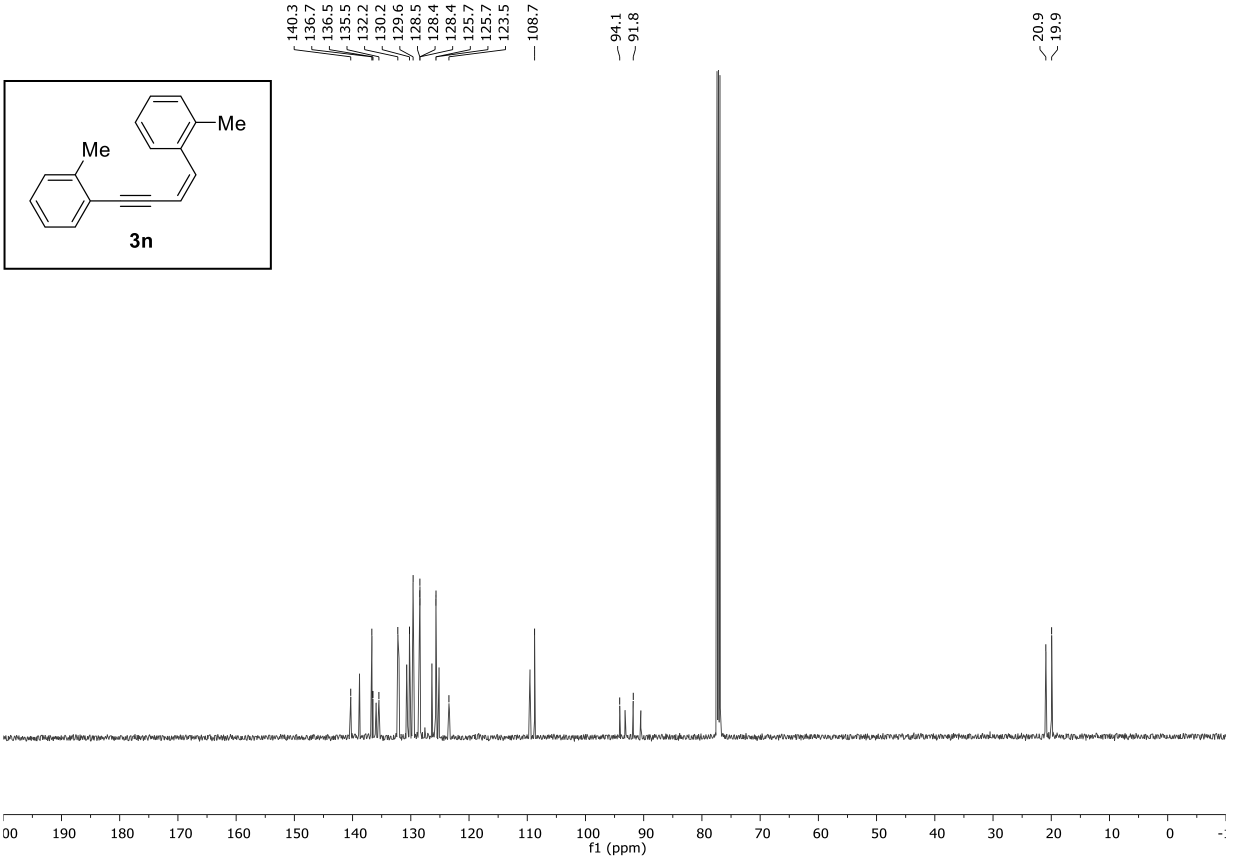
140.3 136.7 136.5 135.5 132.2 130.2 129.6 128.5 128.4 128.4 125.7 125.7 123.5
108.7
94.1 91.8
20.9 19.9
Me
Me
3n
f1 (ppm)

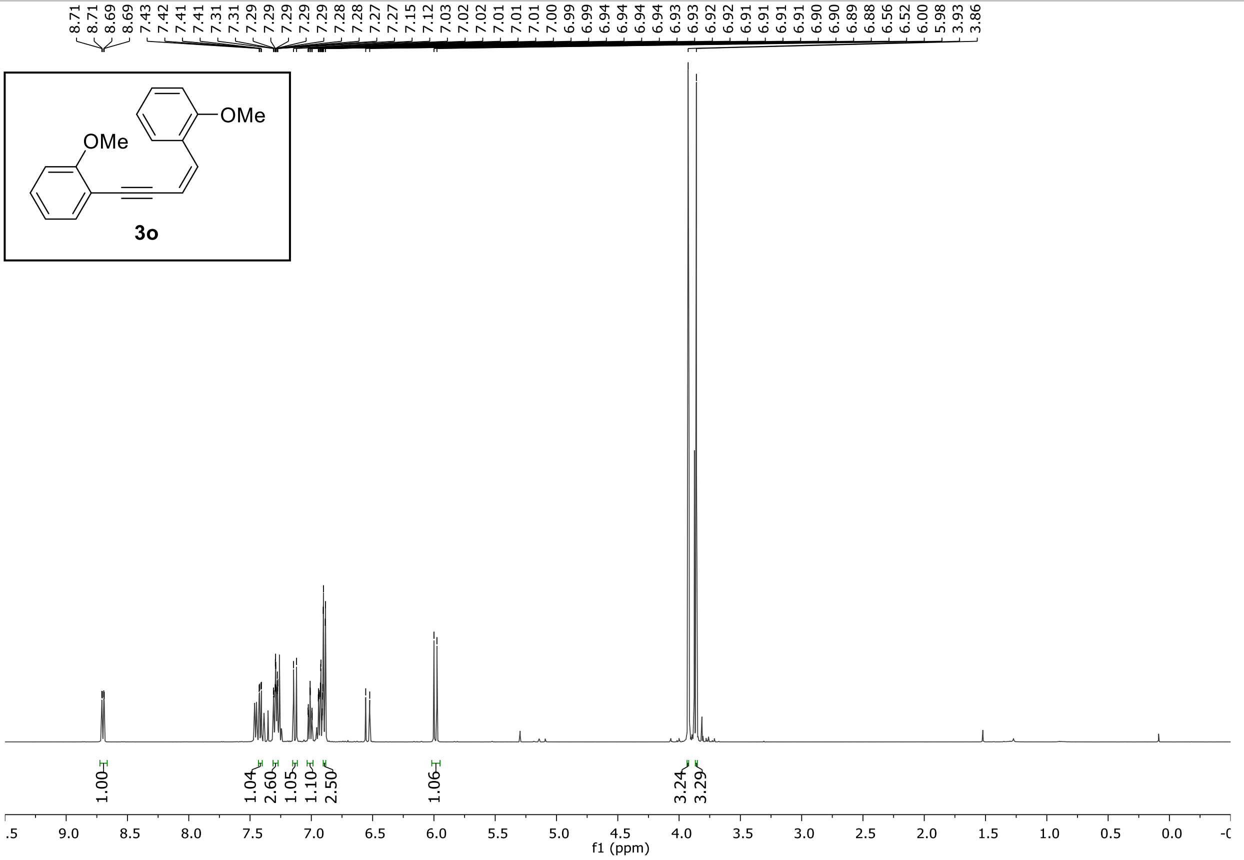
8.71
8.71
8.69
8.69
7.43
7.42
7.41
7.41
7.31
7.31
7.29
7.29
7.29
7.29
7.29
7.28
7.28
7.27
7.27
7.15
7.12
7.03
7.02
7.02
7.01
7.01
7.01
7.00
6.99
6.99
6.94
6.94
6.94
6.94
6.93
6.93
6.92
6.92
6.91
6.91
6.91
6.91
6.90
6.90
6.89
6.88
6.56
6.52
6.00
5.98
3.93
3.86
OMe
OMe
3o
1.00
1.04
2.60
1.05
1.10
2.50
1.06
3.24
3.29
.5
9.0
8.5
8.0
7.5
7.0
6.5
6.0
5.5
5.0
4.5
4.0
3.5
3.0
2.5
2.0
1.5
1.0
0.5
0.0
f1 (ppm)

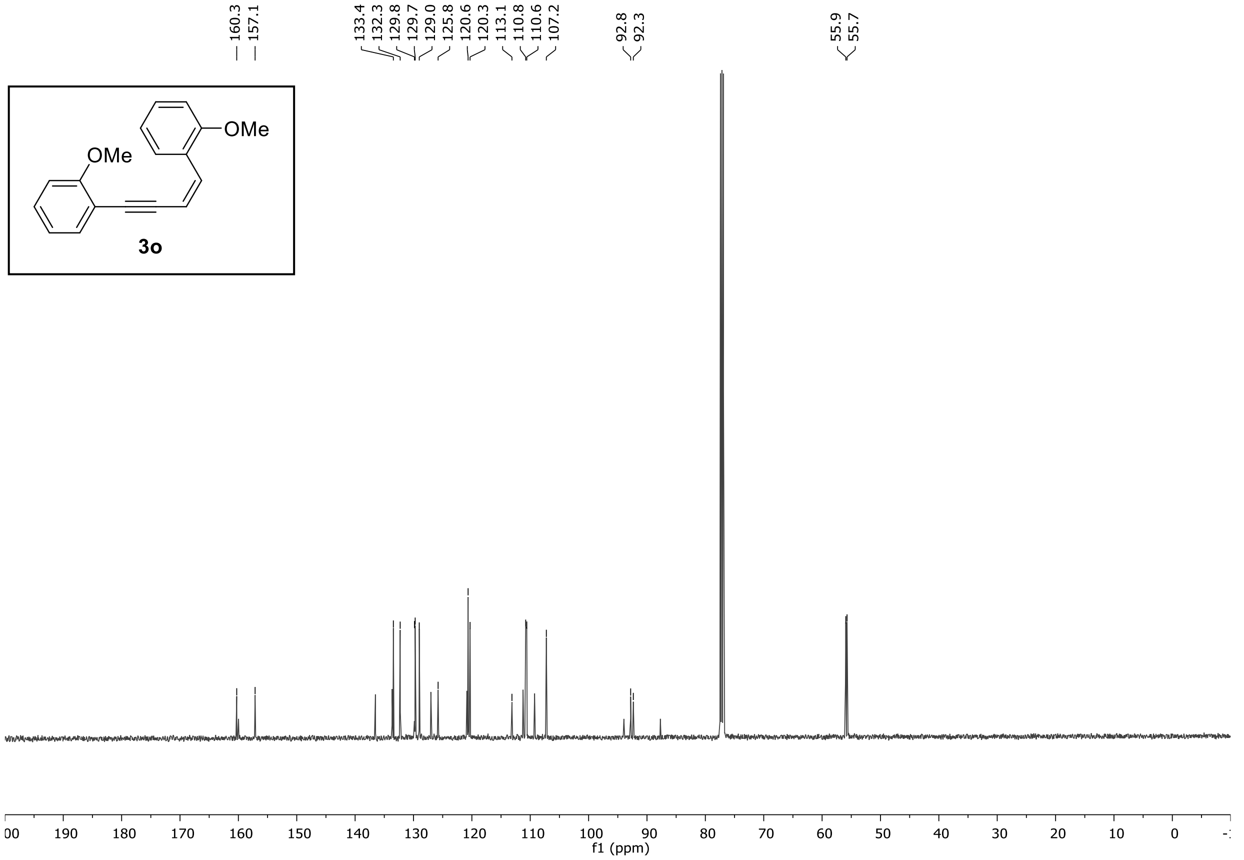
160.3
157.1
133.4
132.3
129.8
129.7
129.0
125.8
120.6
120.3
113.1
110.8
110.6
107.2
92.8
92.3
55.9
55.7
OMe
OMe
3o
190
180
170
160
150
140
130
120
110
100
90
80
70
60
50
40
30
20
10
0
f1 (ppm)

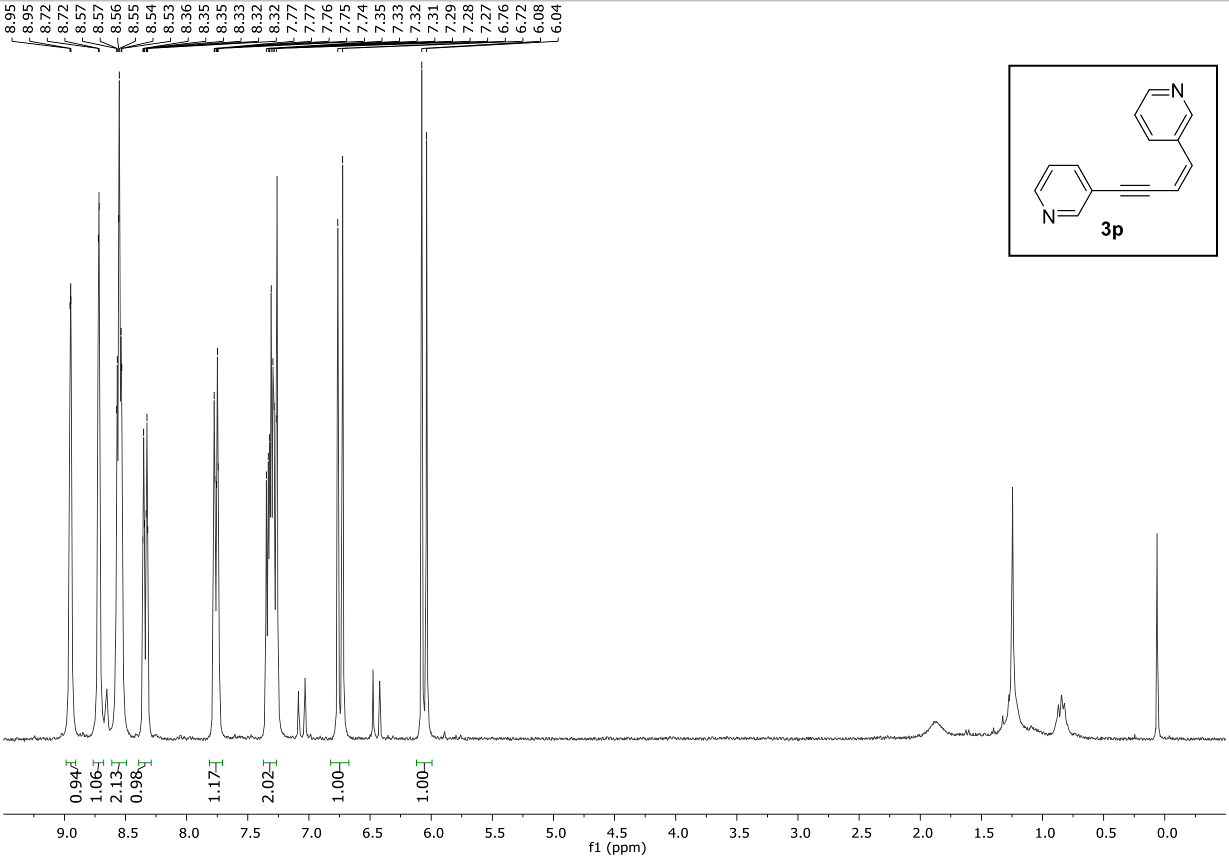
8.95
8.95
8.72
8.72
8.57
8.57
8.56
8.55
8.54
8.53
8.36
8.35
8.35
8.33
8.32
8.32
7.77
7.77
7.76
7.75
7.74
7.35
7.33
7.32
7.31
7.29
7.28
7.27
6.76
6.72
6.08
6.04
3p
0.94
1.06
2.13
0.98
1.17
2.02
1.00
1.00
f1 (ppm)

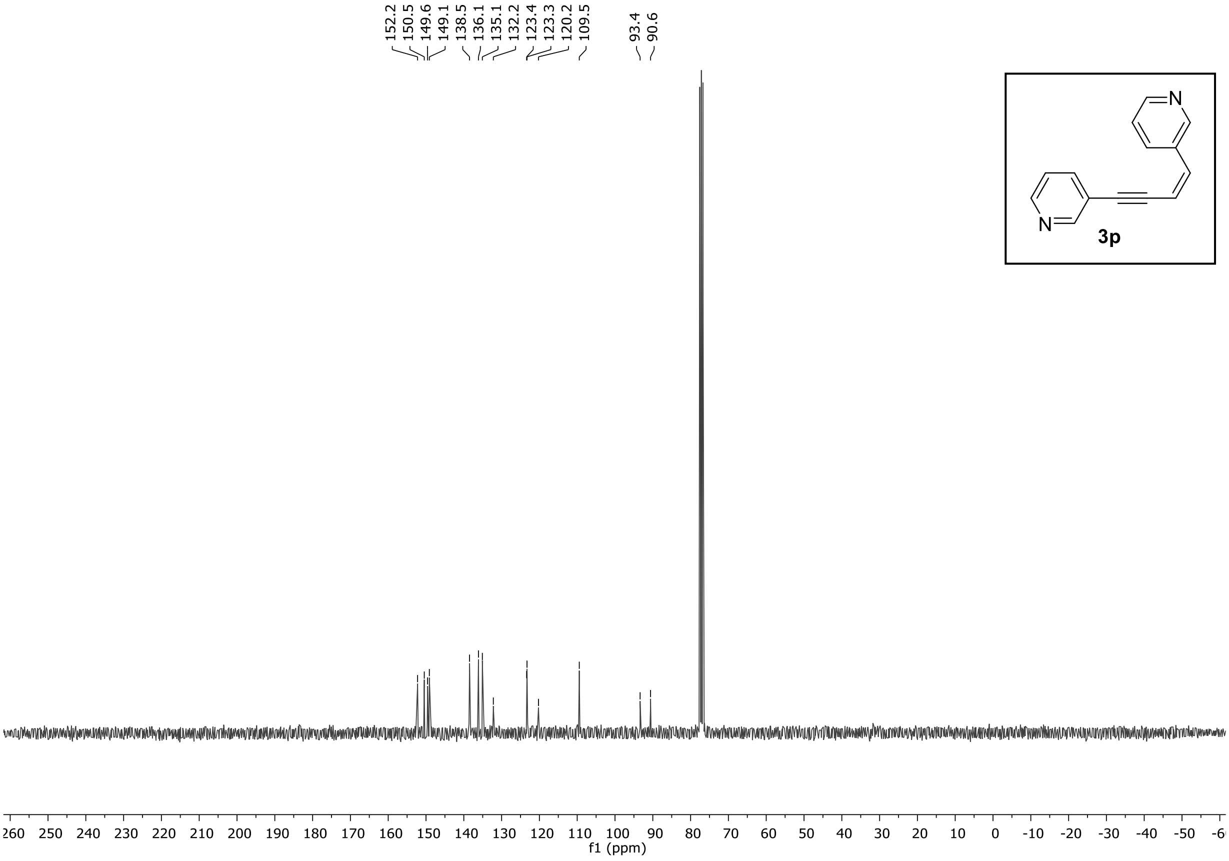
152.2
150.5
149.6
149.1
138.5
136.1
135.1
132.2
123.4
123.3
120.2
109.5
93.4
90.6
3p
f1 (ppm)

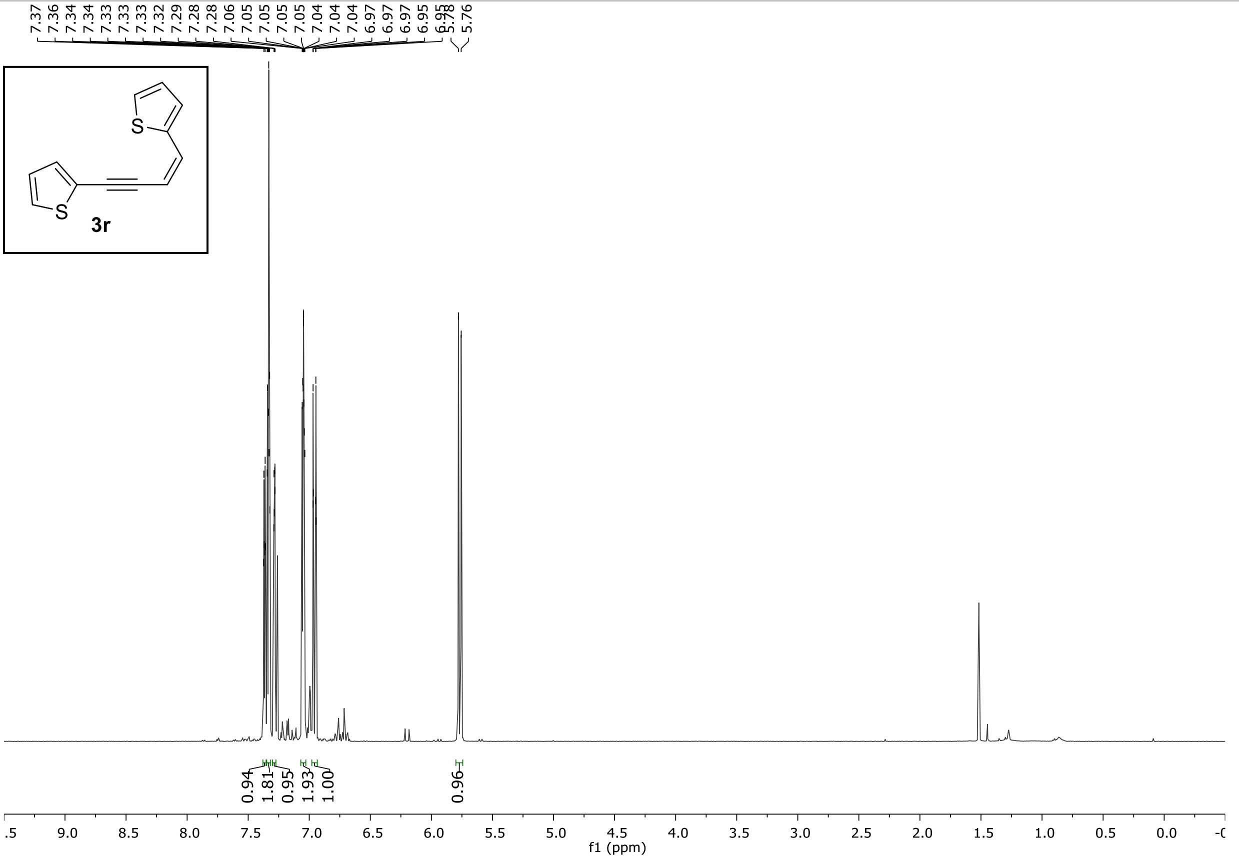
7.37
7.36
7.34
7.34
7.33
7.33
7.33
7.32
7.29
7.28
7.28
7.06
7.05
7.05
7.05
7.05
7.04
7.04
7.04
6.97
6.97
6.95
6.95
5.78
5.76
S
S
3r
0.94
1.81
0.95
1.93
1.00
0.96
f1 (ppm)

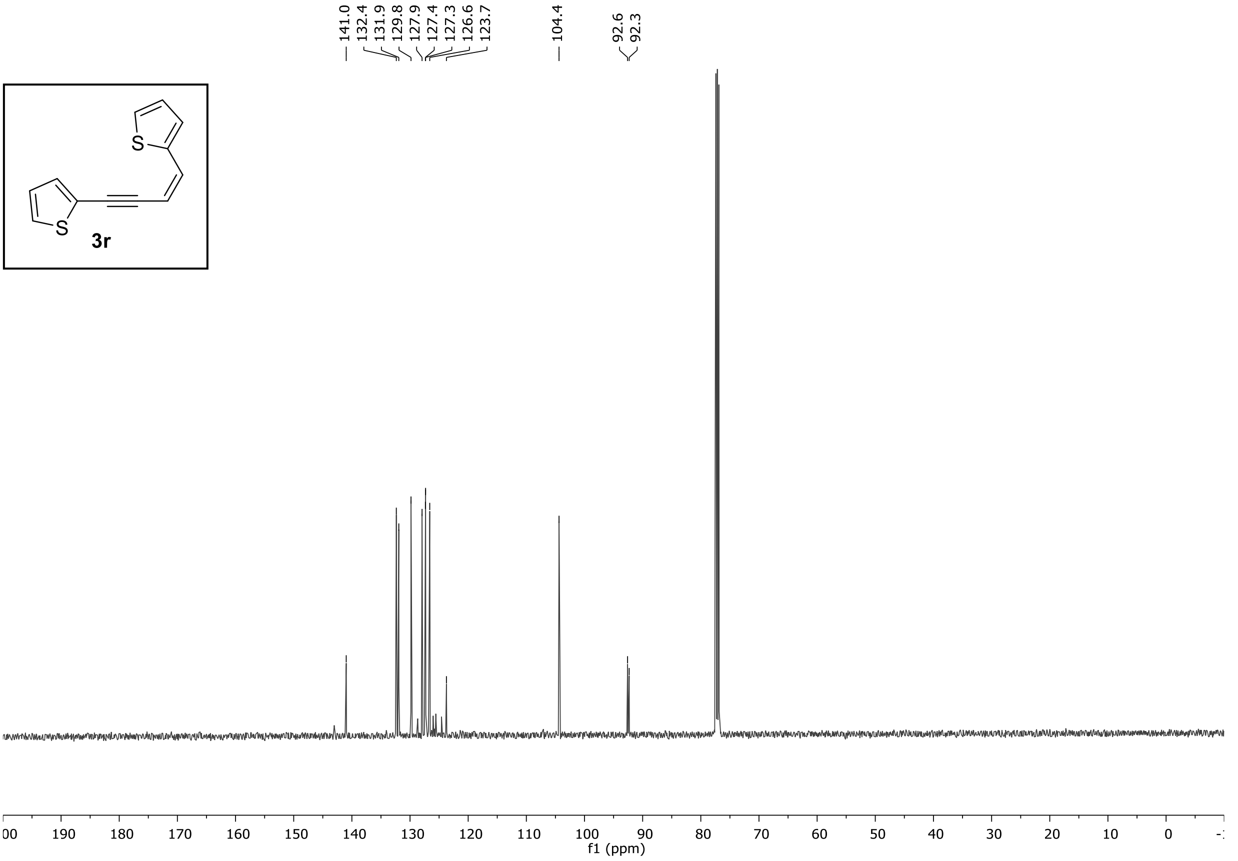
141.0
132.4
131.9
129.8
127.9
127.4
127.3
126.6
123.7
104.4
92.6
92.3
S
S
3r
f1 (ppm)
